# Incomplete lineage sorting shaped mixed traits during a colobine primate radiation

**DOI:** 10.1073/pnas.2524833123

**Published:** 2026-01-23

**Authors:** Yan-Qing Guo, Yiming Wang, Paul A. Garber, Yingchun Li, Ru Zhang, Chi Zhang, Zhipang Huang, Dong-Dong Wu, Bao-Guo Li, Liangwei Cui, Bei Li, Xiao-Guang Qi

**Affiliations:** ^a^College of Life Sciences, Northwest University, Xi’an 710069, China; ^b^School of Stomatology, Air Force Medical University, Xi’an 710032, China; ^c^Department of Anthropology, University of Illinois, Urbana, IL 61820; ^d^International Centre of Biodiversity and Primate Conservation, Dali University, Dali 671003, China; ^e^Gaoligong Mountain Forest Ecosystem Research Station, Kunming Institute of Botany, Kunming 650222, China; ^f^Forestry College, Southwest Forestry University, Kunming 650224, China; ^g^Beijing Genomics Institute, Shenzhen 518083, China; ^h^Kunming Institute of Zoology, Chinese Academy of Sciences, Kunming 650201, China

**Keywords:** incomplete lineage sorting, colobine monkeys, evolutionary radiation, speciation, admixed trait

## Abstract

Lineages undergoing rapid evolutionary diversification may evolve a set of mixed traits as a result of hybrid speciation, gene flow, or incomplete lineage sorting (ILS). However, how best to quantify these alternative processes impacting phenotypic variation remains unclear. Here, we examined trait evolution in two sister genera of endangered Asian primates, *Trachypithecus* and *Semnopithecus*. By assembling a de novo genome of the *Trachypithecus pileatus* group, which is geographically located in a transitional zone between these two genera, our integrated phylogenomic analyses clarified the evolutionary relationship of the *T. pileatus* group with other *Trachypithecus* species. We also identified that morphological similarities shared between this species group and *Semnopithecus* are the result of ILS, rather than gene flow or hybrid speciation. Across all chromosomes of the *T. pileatus* group, ILS contributed 8.9% of whole genome segments. Across these segments, we distinguished 77 genes such as *FGFBP1* and *FOXO1* that are involved in pathways of bone development and osteoblast differentiation. Functional experiments indicate that *FGFBP1* genotypes shared by species of the *T. pileatus* group and *Semnopithecus* appear to enhance osteogenic capability and mineralization, possibly resulting in larger body size and similarities in skull morphology compared with other species of *Trachypithecus*. The study reveals that ILS has shaped the evolution of mixed traits within gene-regulatory networks, driving phenotypic diversity during periods of rapid species divergence in this highly successful primate radiation. ILS appears to be more common than previously thought and represents a critical step in accurately assessing the phylogeny of closely related taxa.

## Significance

Among closely related taxa, both interspecific gene-flow and incomplete lineage sorting (ILS) can result in a mix of traits creating challenges in discerning cladistic relationships. To more precisely examine evolutionary mechanisms driving phenotypic diversity, we used an assembly of high quality de novo genome and phylogenomic analyses to assess polymorphic traits present in two closely related primate genera, *Trachypithecus* and *Semnopithecus*. We identified a set of genotypes and morphological traits in species of the *Trachypithecus pileatus* group and *Semnopithecus* that are the result of ILS, and distinct from other *Trachypithecus* species. Our study provides empirical evidence to distinguish different genomic mechanisms that underlie phenotypic diversity in mammals and offers insights into trait origins and differentiation associated with speciation.

Mixed traits are widely exhibited in extant mammalian lineages, especially in species undergoing rapid periods of evolutionary change and diversification (1–4). This mosaic condition can often confuse traditional morphological boundaries between species or clades, and therefore poses challenges for the accurate delineation of taxonomic relationships (5). Phylogenetic reconstructions employ genetic markers such as microsatellites and mitochondrial DNA to identify the origins and phylogenic relationship underlying polymorphic traits (6, 7). However, these reconstructions often fail, due to limitations in sequence length and insufficient information of functional genetic variation underlying phenotypic changes (8). In addition, the use of different genetic markers in determining phylogenetic relationships may result in alternative topological structures and discordance (9). In contrast, evidence from whole-genome data can provide both a more accurate phylogenetic tree, as well as identify the genetic mechanisms that underpin processes generating mixed traits in the evolution of closely related taxa (10, 11).

There are three primary mechanisms that drive mixed traits during speciation. They include hybrid speciation (12), gene flow between species (13), and incomplete lineage sorting (ILS). ILS involves the random assignment of ancestral allelic variants to different species, resulting in the independent retention of the same genotype in descendant species (14). For example, humans and chimpanzees represent each other’s closest living relatives (15). However, based on their inheritance of shared ancestral genotypes, more than 30% of regions in the human genome show greater similarity with gorillas than with chimpanzees (15). Hybrid speciation, gene flow, and ILS can all lead to trait similarity and phylogeny discordance (9, 14, 16). However, determining which traits are derived from exogenous genetic exchange (hybrid speciation or gene flow) or endogenous polymorphisms (ILS) remains a major challenge.

Primates comprise 527 extant species (17). Compared to many other taxa of large mammals, they exhibit a wide range of diverse phenotypic traits (18). In addition, certain primate clades have undergone recent periods of rapid evolutionary change (19). In the case of Old World monkeys, more than 65% of extant species have undergone speciation events within the last 8 Mya (18), resulting in several closely related species occupying overlapping and neighboring distributions (17, 20). This has resulted in brief and intermittent periods of interspecific gene flow and the transfer of genetic material between conspecific and congeneric species (21). Genetic mechanisms such as gene flow (e.g., baboons, macaques) (4, 22, 23) and hybrid speciation (e.g., snub-nosed monkeys) (3) are reported to underpin widespread phenotypic variation and mixed traits in many primate species. In contrast, although ILS widely exists across entire primate clades (e.g., lemurs, gibbons) (24), and may underlie phenotypic variation, distinguishing ILS from genetic introgression (24) and understanding the set of evolutionary processes driving ILS and trait evolution remain a significant challenge.

**Fig. 1.**
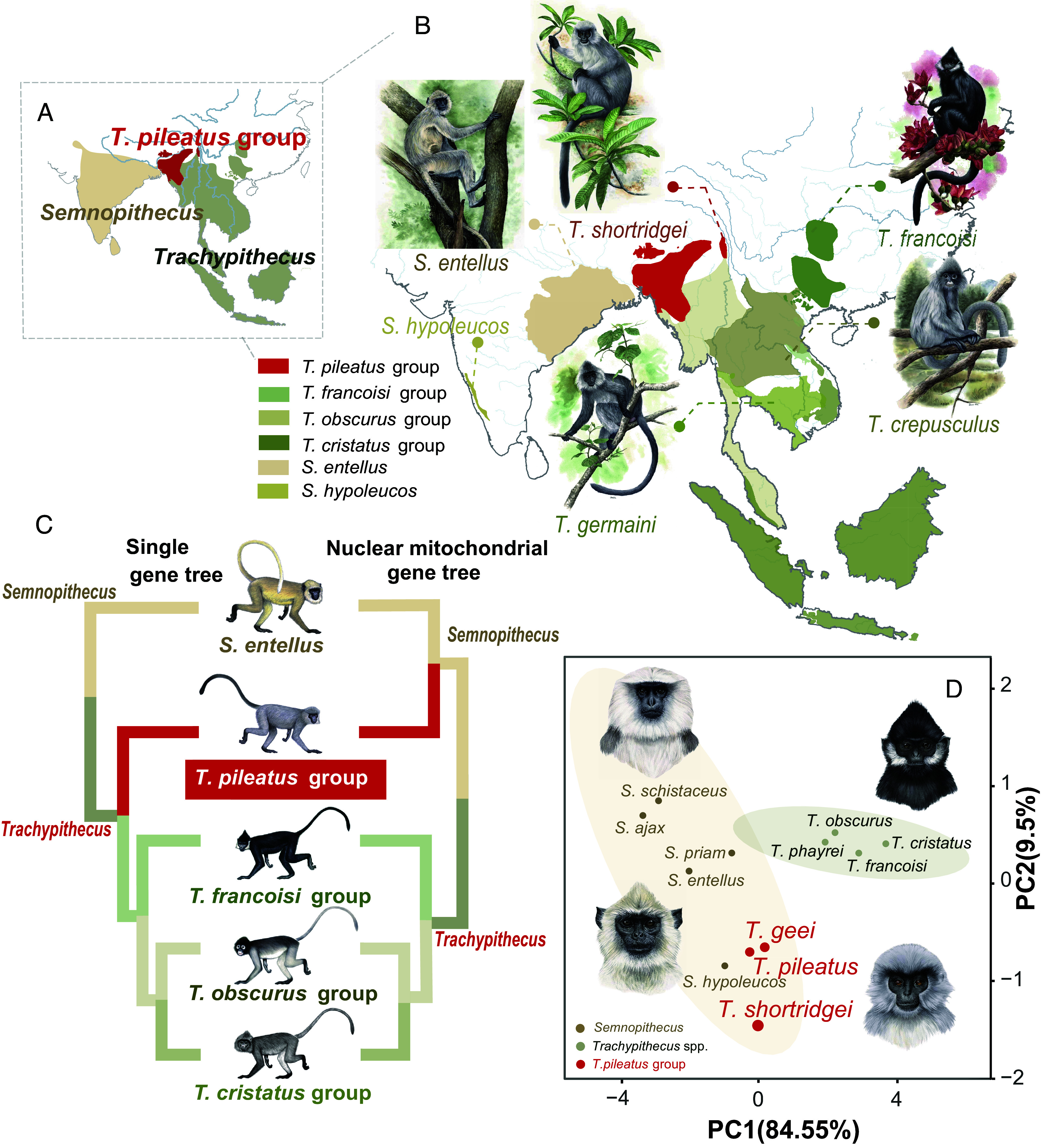
Geographical distribution and taxonomy of the TPG, *Semnopithecus,* and other *Trachypithecus* species. (*A* and *B*) Geographical distribution of the TPG, *Semnopithecus,* and other *Trachypithecus* species (data from IUCN, https://www.iucnredlist.org). (*C*) Phylogenetic relationships among the TPG, *Semnopithecus,* and other *Trachypithecus* species based on single-gene and nuclear mitochondrial gene data. (Monkey illustrations are copyrighted 2014 by Stephen D. Nash/IUCN/SSC Primate Specialist Group and used with permission.) (*D*) Principal components analysis (PCA) of morphological data of representative species from the TPG, *Semnopithecus,* and other *Trachypithecus* species. Other *Trachypithecus* spp. represent species in the genus *Trachypithecus* other than species of the TPG.

Asian colobines represent a highly successful radiation of leaf-eating primates that are distributed across the Indian subcontinent, southwestern China, the Indo-China Peninsula, and the Sundaland (25, 26). This clade, comprising seven genera and 55 species of foregut fermenting primates, exploits a diet characterized by the consumption of difficult to digest cellulose and hemicellulose across a range of diverse habitats (27, 28). The common ancestor of this clade first entered Asia through the Indian subcontinent during the late Miocene (29). They then diverged into two clades (30). The odd-nosed monkeys clade dispersed eastward to the southeastern margin of the Tibetan Plateau, and include snub-nosed monkeys (genus *Rhinopithecus*), doucs (genus *Pygathrix*), simakobus (genus *Simias*), and proboscis monkeys (genus *Nasalis*) (30). The second lineage evolved into the classical langurs. Within this lineage, the genus *Presbytis* separated first and dispersed into Java, Sumatra, and Borneo (26). The genus *Semnopithecus* then split off from their common ancestor and entered the Indian subcontinent, ranging from the southern slopes of the Himalayas to Sri Lanka (Fig. 1*A*). The remaining clade spread eastward through the Indo-China Peninsula into Sundaland, and ultimately diversified into the genus *Trachypithecus* (Fig. 1*A*).

The genus *Trachypithecus* represents the most species and ecologically diverse lineage of Asian colobines (31). It is comprised of four distinct species groups (Fig. 1*B*). The *Trachypithecus francoisi* group, adapted to high-calcium environments and is primarily restricted to karst habitats in Vietnam and southwestern China (27). The *Trachypithecus obscurus* group exploits subtropical to temperate mountainous forest habitats from southwestern China to the Indo-China Peninsula (32). The *Trachypithecus cristatus* group is distributed throughout Sundaland and inhabits forested zones across Southeast Asia (33). Of particular interest, is the *Trachypithecus pileatus* group (TPG), which is composed of three closely related species, the Shortridge’s capped langur (*Trachypithecus shortridgei*), the capped langur (*T. pileatus*), and the golden capped langur (*Trachypithecus geei*) (21). The TPG is primarily distributed across the eastern slopes of the Himalayas, where its distribution overlaps with that of several *Semnopithecus* species, including *Semnopithecus entellus*, *Semnopithecus schistaceus,* and *Semnopithecus hector* in Bhutan, Bangladesh, Myanmar, and northeastern India (Fig. 1*A*). This area represents a transition zone between *Semnopithecus* and *Trachypithecus.* In addition, the three species of the TPG exhibit several intermediate morphological traits, including larger body size and lighter fur coloration that distinguish them from other *Trachypithecu*s species, but are similar to traits present in *Semnopithecus* (34–36). Given these morphological similarities and their overlapping geographical distributions, the phylogeny position of the TPG within *Trachypithecus* remains unclear.

Recent analyses based on multiple nuclear and mitochondrial genetic markers support the clustering of the TPG with other *Trachypithecus* species (35) (Fig. 1*C*). In contrast, studies using partial mitochondrial cytb sequences found the TPG as sister clade to *Semnopithecus* (37, 38) (Fig. 1*C*). Recent phylogenetic analysis revealed that the cytb sequences of the TPG are derived from nuclear mitochondrial DNA (Numt), while the true mitochondrial genome supports a basal placement of TPG within *Trachypithecus* (34). In addition, it has been proposed that the TPG may have originated from hybrid speciation between *Semnopithecus* species and *Trachypithecus* species (34, 38). Thus, conflicting genetic analyses, polymorphic phenotypes, and distributional overlap, raise questions concerning the origin of the TPG, and especially the role of genomic mechanism in shaping phenotypic divergence in this primate radiation.

Based on newly obtained de novo genome data of *T. shortridgei* and our recent phylogenomic analyses of *Semnopithecus* and *Trachypithecus*, we aimed to clarify the phylogenetic position of the TPG and critically evaluate incongruent gene trees that place the TPG closer to *Semnopithecus* than to other *Trachypithecus* species. By integrating multiple methods, we assessed the impacts of hybrid speciation, gene flow, and ILS on the origin of the TPG lineage. We also quantified the proportion of ILS across this radiation and demonstrated a direct link between genomically regulated traits and morphological similarity among these closely related lineages. Our study provides an instructive model for examining trait diversity and phenotypic variation by distinguishing between ILS and multiple genomic introgression events that drive speciation.

## Results and Discussion

### Morphological Variation Among TPG, *Semnopithecus,* and *Trachypithecus*.

In order to measure morphological variation in species of the TPG compared to *Semnopithecus* and other species of *Trachypithecus*, we collected data on five morphological features from 12 representative species of these two genera (*SI Appendix*, Table S1). PCA revealed two distinct morphological clusters were most influenced by zygomatic width, condylobasal length, and body length (PC1 explained 81.73% of variation, Fig. 1*D* and *SI Appendix*, Tables S2–S4). The TPG closely clustered with *Semnopithecus* species and were distinct from other *Trachypithecus* species (Fig. 1*D* and *SI Appendix*, Fig. S2). Both hierarchical clustering and model-based clustering analyses supported these same two morphological clusters (*SI Appendix*, Figs. S3–S5). Moreover, the ANOVA based on PC1 scores (*SI Appendix*, section 1.2) indicated significant differences among the TPG, *Semnopithecus*, and other species of *Trachypithecus* (*P* < 0.05). In particular, species of the TPG were found to differ significantly from other *Trachypithecus* species (*P* < 0.005). These results demonstrated variation of morphological features in this clade, especially those associated with facial characters and body size (Fig. 1*D*).

### De Novo Assembly and Annotation of *T. shortridgei*.

**Fig. 2.**
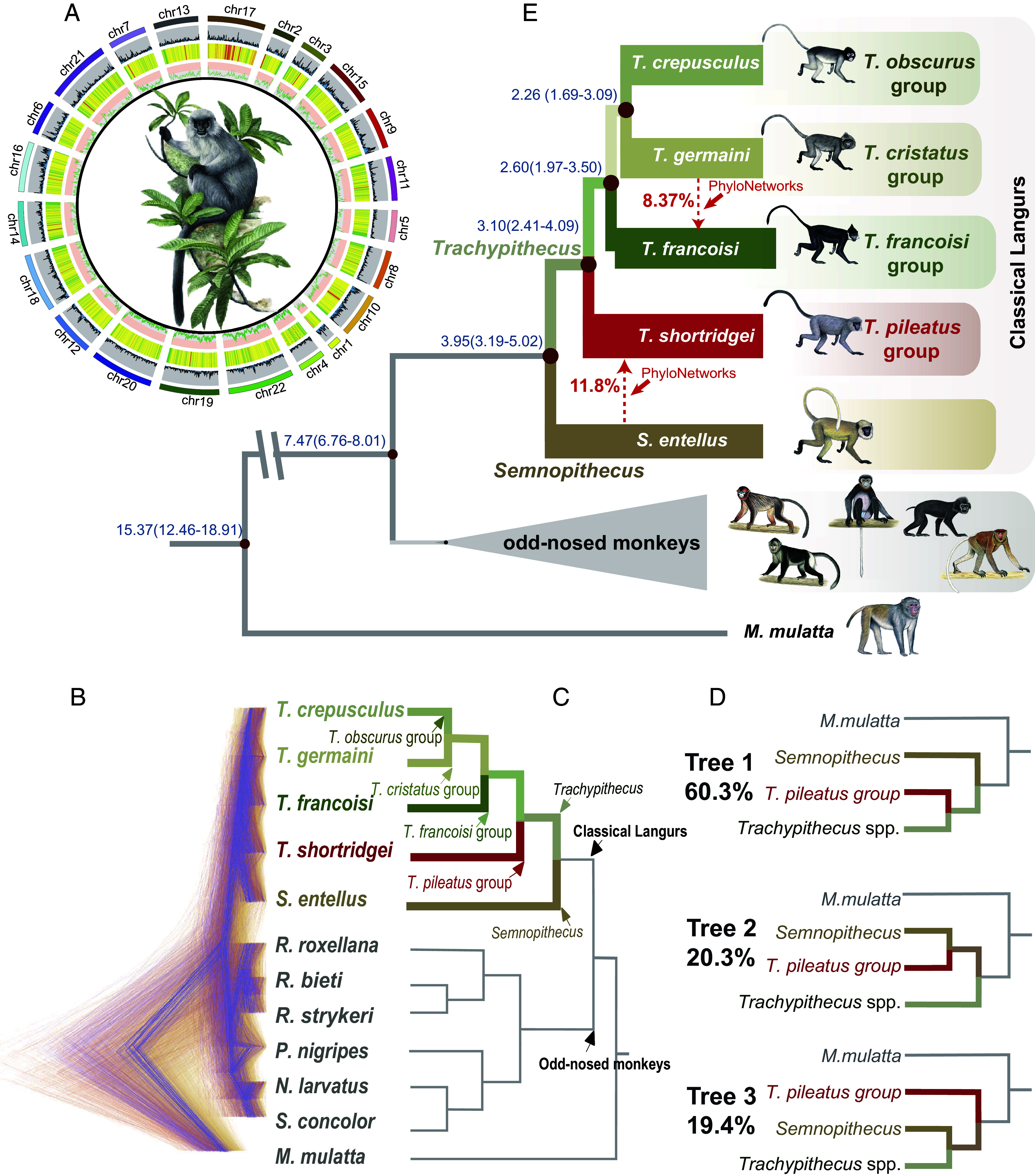
Phylogenetic relationships and discordances among the TPG, *Semnopithecus,* and other *Trachypithecus* species. (*A*) Circos diagram of *T. shortridgei* genome characteristics. chr1 to chr22 refer to the chromosomes sequenced, with circular rings outside to inside referring to the number of coding genes (histogram), repeat elements (heat map), and GC content (line chart) per 500-kb window, respectively. (*B*) DensiTree plot for 1 kb window trees. Gene trees with a clade probability larger than 25% are shown. (*C*) The species tree inferred by ASTRAL and MP-EST from the 18,141 windows. (*D*) The three distinguished topologies based on the 1 kb window trees. *Trachypithecus* spp. represent species in genus *Trachypithecus* other than species of the TPG. (*E*) Robust species tree of the TPG, *Semnopithecus,* and other *Trachypithecus* species. The estimated divergence times (in millions of years) are labeled on each node (branch lengths represent only the topological relationships). The red dashed line denotes gene flow and/or ILS inferred from PhyloNetworks analyses.

To address the current lack of genomic data for species in the TPG, we performed whole-genome sequences in *T. shortridgei* as a representative of this species group (*SI Appendix*, section 1.3). *T. shortridgei* is listed as “Endangered” by the International Union for Conservation of Nature (IUCN 2025), with an estimated remaining wild population of 250 to 370 individuals (39). From Aug. 2020 to Sep. 2023, we followed a wild population of this species in the Dulongjiang region of Gaoligongshan National Nature Reserve, China. We collected a liver tissue sample from an adult male that died of natural causes. Nanopore Long reads (~126.91 × coverage) were used to assemble the raw genome, and Illumina short reads (~77 × coverage) were used to refine the assembly (*SI Appendix*, Tables S5 and S6). Using Hi-C data, we generated 22 scaffolds comprising 21 autosomes and one X chromosome that ranged from 36.12 to 197.27 Mb (Fig. 2*A* and *SI Appendix*, Table S7 and Fig. S7). The final chromosome-level reference size was 2.90 Gb with scaffolds N50 of 134.09Mb (*SI Appendix*, Table S8). The results from BUSCO analyses (40) showed that genome completeness was 96%, indicating reliable quality (*SI Appendix*, Table S9). We annotated the genome using the de novo method and the homology method, which predicted 24,761 protein-coding genes and protein completeness of 97.2% (*SI Appendix*, Tables S10–S13). We next integrated genomic datasets from five species of *Semnopithecus* and *Trachypithecus* and constructed whole-genome datasets for further analyses, with *T. shortridgei* representing the TPG; *T. francoisi* representing the *francoisi* group; *Trachypithecus germaini* representing the *cristatus* group; *Trachypithecus crepusculu*s representing the *T*. *obscurus* group; and *S. entellus* representing species of the genus *Semnopithecus*.

### Phylogeny Reconstruction.

To resolve the long-debated phylogenetic relationships of this clade, we reconstructed a phylogenetic tree with the rhesus macaque (*Macaca mulatta*) as the outgroup. We used a concatenation-based method with 11,193 one-to-one orthologous genes, and fourfold degenerate sites (4DTV) in these orthologous genes (*SI Appendix*, sections 1.4.1 and 1.4.2). The topological structure of the resulting phylogenetic tree indicated that species of the TPG belong to the genus *Trachypithecus* rather than *Semnopithecus*, and represent the *Trachypithecus* basal clade (*SI Appendix*, Figs. S9 and S10).

We further confirmed the topological structure of the tree based on conserved noncoding elements (CNEs) and complete mitochondrial genomes (mtDNA) (*SI Appendix*, sections 1.4.3 and 1.4.4). The results of the CNEs and mtDNA tree were consistent with the orthologous genes tree that identified species of the TPG as a basal lineage of the genus *Trachypithecus* (*SI Appendix*, Figs. S11 and S12). The results from mtDNA showed a distinct position for *Semnopithecus*, which formed an independent branch among Asian colobines (*SI Appendix*, Fig. S12).

Given that the concatenation-based method calculated all orthologous genes and therefore could not detect the possibility that different genomic regions could result in different cladistic trees (9), we extracted 18,141 windows of 1 kb length from whole genomes, and constructed each 1 kb window-based gene tree (WGTs) (*SI Appendix*, section 1.4.5). This resulted in substantial heterogeneities in the gene trees (Fig. 2*B*). We filtered the resulting trees with probabilities of less than 25% to minimize the negative impacts of noise (*SI Appendix*, section 1.4.5). The results show that 60.3% of the trees were consistent with the topology from orthologous genes that assigned species of the TPG as the basal clade among *Trachypithecus* species and *Semnopithecus* (Tree 1, Fig. 2*D*). In addition, 20.3% of trees supported species of the TPG as part of a clade with *Semnopithecus* (Tree 2), and 19.4% of the topologies supported a trichotomy with species of the TPG separate from other species of *Trachypithecus* and *Semnopithecus* (Tree 3, Fig. 2*D*). We also constructed 10 kb and 50 kb WGTs using the same methods to verify the reliability of the results (*SI Appendix*, Fig. S13). This analysis supported Tree 1 as having the highest proportion of all substantial heterogeneities, which was significantly higher than that of Trees 2 and 3 (*P* < 0.00063 and *P* < 0.00061, respectively).

Based on 1 kb WGTs derived from each chromosome, we integrated all topological heterogeneities to reconstruct a single robust species tree using coalescent-based methods with ASTRAL-III (41) and MP-EST (42). This was done to provide a framework for the following analyses. The results demonstrate that the tree topologies obtained from concatenation-based and coalescence-based methods were consistent, placing the TPG as the basal clade within the genus *Trachypithecus* (Fig. 2 *C* and *E* and *SI Appendix*, Fig. S15). In addition, sex chromosomes may contain important heterogeneity information (43). Based on WGTs independently derived from X chromosomes, we constructed the species tree using the same framework. The results continued to support the TPG as the basal clade within the genus *Trachypithecus* (*SI Appendix*, Fig. S16). Therefore, we used the robust Tree 1 as the speciation tree in downstream analyses.

### Rapid Evolutionary Radiation of *T. pileatus* Group.

To further understand the evolutionary history of the TPG, *Semnopithecus,* and other species of *Trachypithecus*, we estimated their divergence times based on the topology of the species tree, 4DTV site alignments, and fossil calibration across time points (*SI Appendix*, section 1.5.1). The results indicate that the genus *Trachypithecus* and *Semnopithecus* diverged approximately 3.95 (3.19 to 5.02) Mya (Fig. 2*E* and *SI Appendix*, Fig. S17), and species of the TPG diverged from the other *Trachypithecus* species groups approximately 3.10 (2.41 to 4.09) Mya (Fig. 2*E* and *SI* *Appendix,* Fig. S17). Then *T. francoisi,* representing the *T. francoisi* group, radiated from other *Trachypithecus* species approximately 2.60 (1.97 to 3.50) Mya, while *T. germaini,* representing the *T. cristatus* group, and *T. crepusculus,* representing the *T. obscurus* group, diverged about 2.26 (1.69 to 3.09) Mya from their common ancestors (Fig. 2*E* and *SI Appendix*, Fig. S17). Pairwise sequentially Markovian coalescent (PSMC) analysis (44) revealed that the common ancestor of *Trachypithecus* and *Semnopithecus* maintained a large effective population size (*SI Appendix*, Fig. S18). This population then experienced a pronounced decline approximately 4 to 6 Mya (*SI Appendix*, Fig. S18), coinciding with the successive divergence time of *Semnopithecus*, the TPG, and the other *Trachypithecus* species. These rapid speciation events provide important evidence for understanding mixed trait and genomic mechanisms that contributed to evolutionary change.

### Genetic Admixture Signals between TPG and *Semnopithecus* Species.

Given the presence of mixed traits and geographic overlap that characterize species of the TPG and *Semnopithecus,* several studies have hypothesized a hybrid speciation event (34, 37, 38). Theoretically, if hybrid speciation has occurred the proportion of species clustered with its maternal species should be expected to be equal to its paternal species (3). However, the WGTs results indicate that the proportion of species in the TPG clustering with *Trachypithecus* (Tree 1) was significantly higher than the proportion of TPG species clustering with *Semnopithecus* (Tree 2). Moreover, if hybrid speciation has occurred, the proportion of species clustered with each of its parental species also is expected to be higher than the proportion of species clustered with its nonparental species (3). However, the proportions of Tree 2 were not statistically higher than other topological heterogeneity (Tree 3). This indicates that these results are inconsistent with a hybrid speciation origin for the TPG. Therefore, we conducted a PhyloNetworks analysis (45) to evaluate the hybridization signal among five species, representing each of the *Trachypithecus* species groups and species of *Semnopithecus* (*SI Appendix*, section 1.6.1). The results indicate that 11.80% of the *S. entellus* genome was transferred to *T. shortridgei*, while 8.37% of the *T. germaini* genome was transferred to *T. francoisi* (Fig. 2*E*), each deviating significantly from 50%. These results fail to support the hybrid speciation hypothesis and indicate that the set of mixed traits present in species of the TPG are best explained by gene flow and/or ILS.

We next used an ABBA-BABA *D*-statistics (13) to test for the presence of gene flow. The results showed no evidence of gene flow between *T. shortridgei* and *S. entellus* (*SI Appendix*, Table S14). However, the absolute *D*-values were greater than 0 in some species pairs, suggesting the presence of interspecies gene flow (*SI Appendix*, Table S14). We further applied the Weighted Block Jackknife method to evaluate the significance of *D*-values. The results validated the existence of gene flow (|*Z*-values| > significant level 3, *SI Appendix*, Table S14). The low level of *D*-values may imply that false positives may arise due to the difference in population size among species, or from ancient gene flow that occurred in the ancestral clade, rather than recent interspecies gene flow (46).

**Fig. 3.**
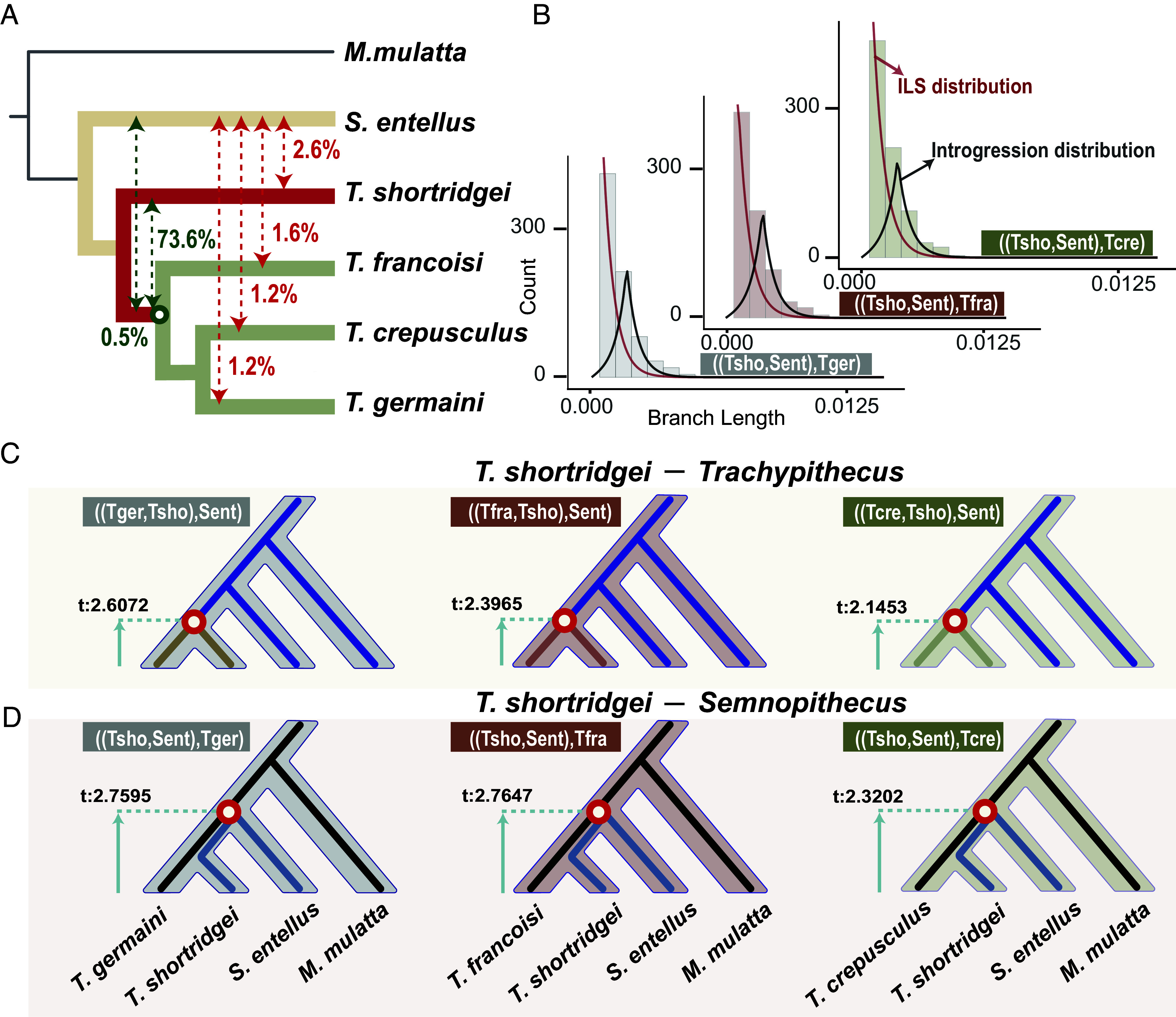
Tests for gene flow and ILS. (*A*) The proportions of gene flow inferred by *D*_FOIL_ analysis based on 100 kb windows. Red numbers represent the calculation of recent gene flow, while green numbers represent the calculation of ancient gene flow. (*B*) The distribution of the internal branch lengths. The histograms represent the distribution of internal branch lengths from the topology that places *S. entellus* and *T. shortridgei* as sister species. The red curves represent the expected distributions under the ILS-only model and black curves represent under ILS and gene flow model. Tger represents *T. germaini*, Tfra represents *T. francoisi*, Tcre represents *T. crepusculus*, Tsho represents *T. shortridgei*, and Sent represents *S. entellus*. (*C* and *D*) Estimated divergence times to distinguish between ILS and gene flow scenarios. The numbers in (*C*) represent divergence times (t) in the robust species tree. The numbers in (*D*) represent the divergence times (t) of the topologies that support *T. shortridgei* clustered with *S. entellus* as sister species.

We used a *D*_FOIL_ analysis (47) to quantify recent gene flow between extant species and ancient gene flow at the ancestral node. We used four representing species to construct an asymmetric five-taxon phylogenetic framework, with *M. mulatta* as the outgroup and then divided the alignment across whole-genomes into 100 kb windows each (*SI Appendix*, section 1.6.2). By comparing patterns of allele sharing between all pairs of extant species, we distinguished the windows showing gene flow signals (*SI Appendix*, section 1.6.2). The results indicated that 75.8% of the windows were detected with signals, with 73.6% indicating ancient gene flow, and 2.2% indicating recent gene flow within the internal branches of the *Trachypithecus* genus (Fig. 3*A* and *SI Appendix*, Table S18). Because this method can only provide a maximum of a five-taxon comparison, whereas we need to examine six species (including one species in *Semnopithecus* and four species groups in *Trachypithecus*, with *M. mulatta* as the outgroup), we conducted iterations by switching out species (except *S. entellus* and *T. shortridgei*) and reperformed the analysis (*SI Appendix*, section 1.6.2). The results remained consistent (*SI Appendix*, Table S18), indicating a low level of recent gene flow within these species.

We next quantified recent gene flow between *T. shortridgei* and *S. entellus* within a four-taxon phylogenetic framework (*SI Appendix*, section 1.6.2). The results also showed a low level of recent gene flow of 2.6% (Fig. 3*A* and *SI Appendix*, Table S19). Although population genomic analyses based on a larger number of species could help to validate cases of additional hybrid speciation and recent gene flow, given the endangered status and broad distribution of many *Trachypithecus* and *Semnopithecus* species, a more comprehensive evaluation is unlikely in the near future. Therefore, we used the length of segments to confirm the low level of recent gene flow across an independent method (*SI Appendix*, section 1.6.2). As systematic genomic recombination is expected to occur over evolutionary time, it will split the entire region into separated fragments (46). Therefore, if recent gene flow occurred, the introgression segments from gene flow should be expected to exhibit consecutive segments in the genome. We examined the distribution of windows with a gene flow signal. The results found that the windows were discrete and noncontiguous (*SI Appendix*, Fig. S19 and Table S17), indicating a limited amount of recent gene flow and consistent with the *D*-statistic results.

### ILS Causes Heterogeneity in the TPG Phylogenetic Position.

We next distinguished between ancient gene flow and ILS as the primary explanations for the observed phylogenetic discordances among the TPG species. In theory, gene flow can reduce the genetic difference between two species, making their calculated genetic distance appear closer than their actual genetic distance (48). When clustering these species together a gene flow event would be expected to result in an elongated internal branch length compared with the branch length in a robust species tree (49). In contrast, ILS originated from the random retention of ancestral polymorphic allelic variation and result in a relatively shorter internal branch length (49). We applied a QuIBL analysis (49) to discriminate these two processes based on a comparison of internal branch lengths in multiple topological trees (*SI Appendix*, section 1.6.3). The internal branch lengths from discordant topology that placed *S. entellus* and *T. shortridgei* as sister species is consistent with the expectations under the ILS-only model (ΔBIC > 10, Fig. 3*B* and *SI Appendix*, Table S20). This suggests that instead of ancient gene flow, ILS is the main factor behind phylogenetic discordance between the TPG and *Semnopithecus*. Moreover, the phylogenetic discordance between the TPG and other *Trachypithecus* species is primarily due to both ILS and ancient gene flow (*SI Appendix*, Table S20), thus helping to explain the complex evolutionary history of this lineage.

We used an independent framework based on estimated divergence times to validate these results. Given that ILS regions represent the retention of ancestral variation that occurred prior to their divergence, the calculated divergence times should be earlier than the actual time of the speciation event (50). In contrast, gene flow via introgression is expected to occur after speciation (50). To distinguish between ILS and gene flow, we used the MCMCTree (51) to estimate and compare divergence times for discordant topological trees in each window and the robust species tree (the discordant topological trees were selected by supporting the TPG clustering with *Semnopithecus,* *SI Appendix*, section 1.4.5). The results show that the estimated divergence time between the TPG and *Semnopithecus* is earlier in the discordant topology than in the robust species tree (Fig. 3*C* and *SI Appendix*, Fig. S20). The divergence time between the TPG and other *Trachypithecus* species is later in the discordant topology than in the robust species tree (Fig. 3*C* and *SI Appendix*, Fig. S20). These results are consistent with the finding from QuIBL analysis, suggesting that ILS is the main factor shaping phylogenetic discordance between the TPG and *Semnopithecus*, but that ancient gene flow existed between the TPG and other *Trachypithecus* species.

### Distinguish ILS Region in TPG.

**Fig. 4.**
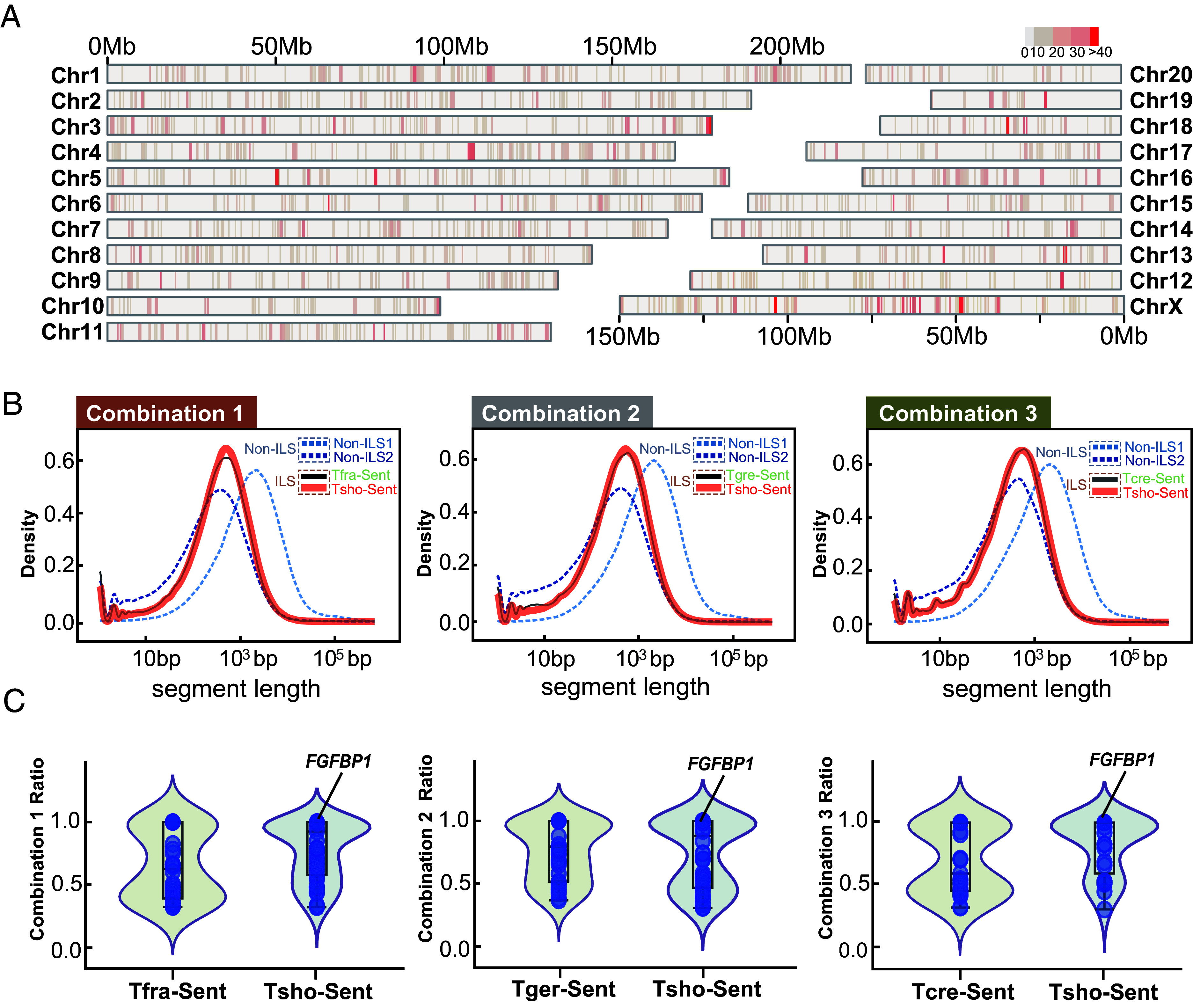
The distributions and length of ILS segment. (*A*) The distributions of ILS segments in *T. shortridgei* using the *M. mulatta* (rhesus macaque) genome coordinates as a reference. Redder colors indicate a higher aggregated level of ILS segments for that region. (*B*) The comparison of lengths and its distributions between ILS and non-ILS segments. Combinations 1, 2, and 3 represent three possible topologies. The X-axis shows the length of ILS segments, and the Y-axis shows the density of the different ILS segment lengths. Tger, Tfra, Tcre, and Tsho represents the *T. germaini*, *T. francoisi*, *T. crepusculus*, *T. shortridgei*, respectively. Non-ILS1, Non-ILS2, Tsho-Sent, Tfra/Tgre/Tcre-Sent represents four scenarios (type0, type1, type2, type3) in CoalHMM analysis, respectively. (*C*) The proportion of ILS segments covering coding regions for each gene. The *FGFBP1* gene used in our cellular experiments is labeled.

To understand the mechanisms by which ILS has shaped the mixed traits in TPG, we applied the coalescent hidden Markov model (CoalHMM) approach (52) to identify the ILS signal among the whole-genome (*SI Appendix*, section 1.6.4). Restricted by the maximum of species number in this approach, we used three representative species to construct four-taxon alignments (Combination 1), with *M. mulatta* as the outgroup. The alignments were divided into 1 Mb windows, and then we identified ILS signal based on the calculation of posterior probabilities (*SI Appendix*, section 1.5.4). We evaluated the ILS signal of each site based on the positional information within the identified windows. We next defined the ILS segment under the condition of several sites showing ILS signals were consecutive, and could have merged into an entire segment (52) (*SI Appendix*, section 1.6.4). Genomic recombination over time is expected to continuously fragment the entire region into short segments (50). The results showed the ILS segments averaged 742 bp in length (Fig. 4*B*), and significantly shorter than those segments unaffected by ILS (Welch’s *t* test, *P* < 0.001) (Combination 1, Fig. 4*B*).

Furthermore, the results showed that the ILS segments occupied 8.9% of whole genome in *T. shortridgei* across all chromosomes, but only occupied 0.7% of coding regions (*SI Appendix*, Table S21). We performed the same analyses by changing the representative species pairs (Combination 2 and Combination 3, *SI Appendix*, section 1.6.4). These results showed the same pattern (*SI Appendix*, Table S21 and Figs. S21–S25), with the proportion of ILS sequences in coding regions significantly lower than in whole genomes in all three combinations (Welch’s *t* test, *P* < 0.001). This supports that coding regions under stronger purifying selection tend to have fewer ILS.

To investigate the distribution of ILS segments in *T. shortridgei*, we mapped these ILS segments onto a reference genome of *M. mulatta* across different chromosomes (Fig. 4*A*). The results revealed different aggregated levels of ILS in different genomic regions, implying repeated selective sweeps or strong background selection (53) and balancing selection (24). Interestingly, a lower aggregated level should be expected on X chromosomes, because males only carry a single copy of the X chromosome, which could reduce the calculations of effective population size (*Ne*) (24). However, our results found an opposite pattern, the X chromosome showed a higher aggregated level than did autosomes (Fig. 4*A*). Although the low levels of ILS in X chromosome were widely observed in multiple primate taxon, this higher level of ILS signal and the higher effective population size based on results from the X chromosome have also been estimated in the entire radiation of Asian colobines (24). Like those of Asian colobines, *T. shortridgei* exhibit a polygynous mating system (54). Under conditions, in which males exhibit pronounced reproductive skew, the X chromosome is expected to have a higher *Ne* than autosomes (55, 56). This helps to explain the higher ILS signal in the X chromosome of *T. shortridgei*.

### Functional Genes Distinguished from ILS.

To identify the functional genes from the ILS segments in orthologous coding regions (*SI Appendix*, section 1.7), we applied standard in which the ILS segment covered more than 30% of the coding regions of a gene (50) (Fig. 4*C*). We obtained 77 ILS genes and then annotated these genes to the Gene Ontology (GO) terms and the Kyoto Encyclopedia of Genes and Genomes (KEGG) pathways using KOBAS (57). The results indicated most of the high-ranking terms and pathways were involved in cellular basic metabolic processes and immune responses (*SI Appendix*, Fig. S26 and Tables S22 and S23). This is consistent with previous explanations that immune response regulation is known to have evolved under balancing selection in primates (58). These ILS regions, under balancing selection, can exhibit high functional diversity and were retained in the population in response to selective advantage.

Focusing on the sequence of 77 ILS genes, we examined shared specific amino acid sites between the TPG, *Semnopithecus*, and the other species of *Trachypithecus*. We detected 25 ILS genes in *T. shortridgei* and *S. entellus* that shared the same amino acid sequence and were distinct from other *Trachypithecus* group species at orthologous sites (*SI Appendix*, Table S26). In particular, the IL17REL, TRBV12-3, and OR5A1 present 8, 7, and 7 shared amino acid mutations, respectively (*SI Appendix*, Table S26). The *IL17REL*, *TRBV12-3* genes are involved in immune responses (59, 60), whereas *OR5A1* was associated with olfactory receptors (61). Species of the TPG and *S. entellus* inhabit similar and overlapping environments, exploit similar ecological niches, and appear to exhibit the same genotype for these genes, which has been maintained under similar selective pressures (62).

The enrichment analyses from 25 candidate genes further distinguished terms involved in bone development, including positive regulation of fibroblast growth factor receptor signaling pathways, fibroblast growth factor binding and growth factor binding (*SI Appendix*, Table S24). These biological processes can result in larger body size and changes in skull characters, which may offer crucial clues in understanding mixed traits in zygomatic width and condylobasal length in species of the TPG, which we identified during morphological analyses (*SI Appendix*, Tables S3 and S4).

### ILS Genes Associated with Morphology.

To understand the impact of ILS genes on the morphological variation observed in the TPG, we examined the expression of 25 ILS candidate genes by using data from the Gene Expression Database (63). We focused on skeletal development. We distinguished *FGFBP1*, *BORA*, *FOXO1*, *ENTPD3,* and *UNC5CL* that are engaged in skeletal and skeletal muscular development (*SI Appendix*, Table S26). In particular, *FGFBP1* is reported to be involved in the premature closure of cranial sutures (64) and *FOXO1* is associated with osteoblast number (65).

Notably the *FGFBP1* in the TPG showed the highest proportion of covering rate within the ILS segment, covering 100% of its sequence among all candidate genes, indicating a stronger influence by ILS (Fig. 4*C*). This gene is known as a binding partner of the fibroblast growth factor family (FGF) (e.g., *FGF1*, *FGF2*, *FGF7*, *FGF10*), which could enhance the activity of this gene family on osteogenic cells (66). Among these factors, FGF2 plays an essential role in the anabolic actions of the parathyroid hormone (PTH), as well as in bone morphogenetic protein activity (BMP-2), thereby affecting bone development (67, 68).

**Fig. 5.**
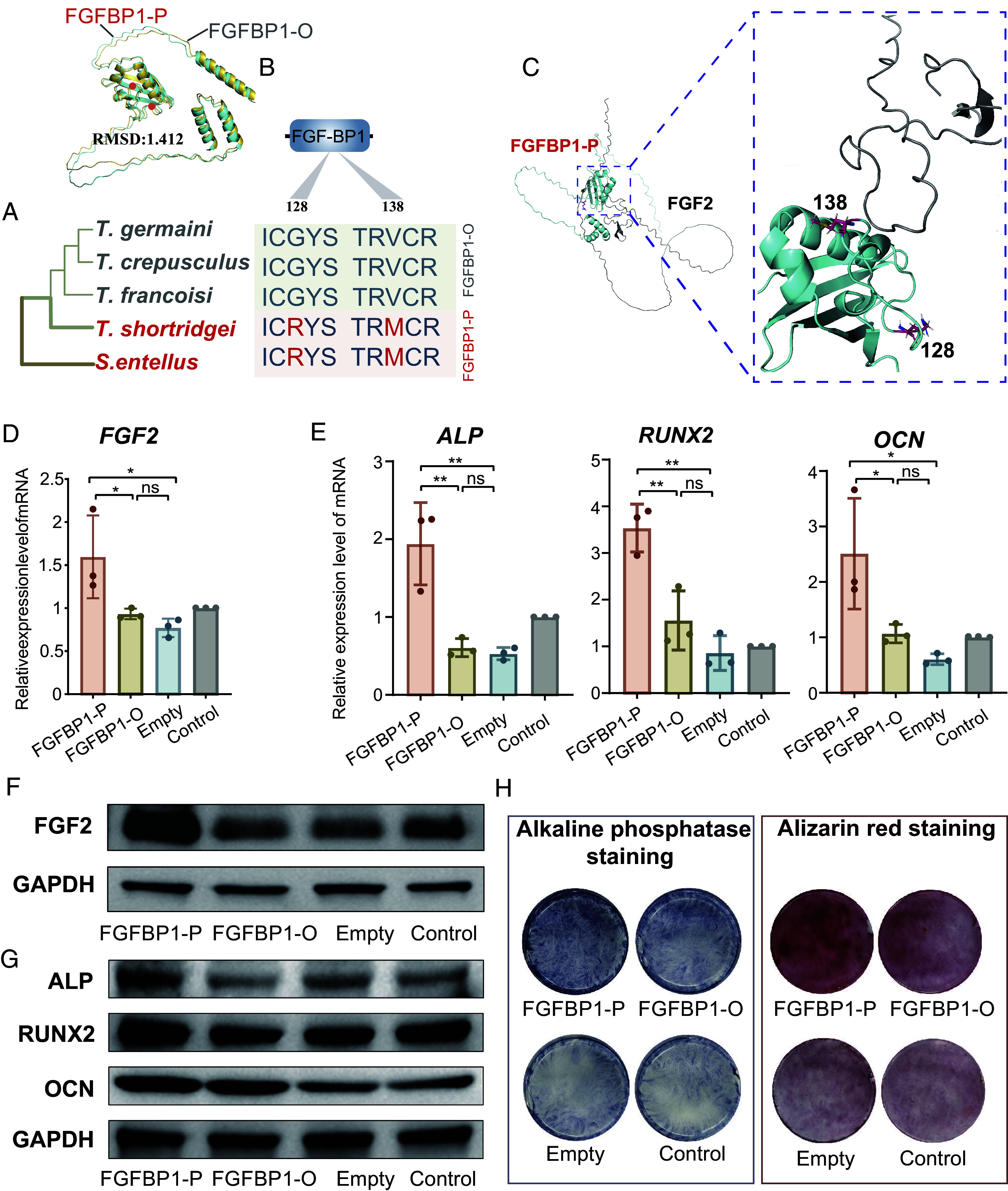
Experimental evidence for ILS genes affecting bone development. (*A*) The amino acid sequence differences of FGFBP1 in *Semnopithecus* and *Trachypithecus* species. FGFBP1-P represents the shared genotype between the TPG and *Semnopithecus*, whereas FGFBP1-O represents the genotype in other *Trachypithecus* species. (*B*) The predicted 3D protein structure of FGFBP1. FGFBP1-P is shown in blue, and FGFBP1-O is shown in yellow. (*C*) Spatial structure of the interaction between FGFBP1 and FGF2 proteins. A close-up of the interaction interface is shown in the image to the right. (*D* and *E*) Comparisons of mRNA expression levels of *FGF2*, *ALP*, *RUNX2,* and *OCN* between FGFBP1-P and FGFBP1-O group. **P < 0.01, *P < 0.05, ns: not significant. The empty plasmid was used for normalization. (*F* and *G*) Comparisons of the levels of protein expression in FGF2, ALP, RUNX2, and OCN between the FGFBP1-P and FGFBP1-O groups in HJBMMSCs. (*H*) Osteogenic differentiation staining between the FGFBP1-P and FGFBP1-O groups in HJBMMSCs.

Moreover, FGFBP1 in *T. shortridgei* and *S. entellus* share specific mutations of arginine (R) to glycine (G) in amino acid positions 128, and methionine (M) to valine (V) in position 138 (Fig. 5*A* and *SI Appendix*, Figs. S27–S29). By constructing three-dimensional structures modeling and nonoverlapping conformations, the results predicted that the spatial structure of the FGFBP1 changed after mutation (Fig. 5*B*). The mutation sites located in the interaction region of the functional domain, may affect FGFBP1 binding to FGF2 (Fig. 5*C*). For the FOXO1 protein, *T. shortridgei* and *S. entellus* share the mutations of alanine (A) to threonine (T) at amino acid position 353, and the nonoverlapping conformations also predict that the spatial structure of FOXO1 has changed after these mutations (*SI Appendix*, Fig. S27). Therefore, we were able to validate the functional change on *FGFBP1* and *FOXO1* using in vitro experiments.

### Functional Experiments of *FGFBP1* in Bone Development.

To validate the functional change in the expression of *FGFBP1* and *FOXO1*, we devised two group plasmids, FGFBP1-P and FOXO1-P, representing the genotypes of *T. shortridgei* and *S. entellus*, respectively. In contrast, the FGFBP1-O and FOXO1-O represented the other *Trachypithecus* species groups (*SI Appendix*, section 1.9.1). These plasmids were subsequently transfected into Human Jaw Bone Marrow Mesenchymal Stem Cells (HJBMMSCs) (*SI Appendix*, section 1.9.1).

The binding of FGFBP1 to FGF2 protects FGF2 from degradation and promotes the biological function of FGF2 (69). FGF2 can function to up-regulate the expression of osteogenic marker genes (*ALP*, *RUNX2*, *OCN*), which reflect the efficiency of osteoblast cell differentiation (70). We assessed differences in the expression of the gene *FGF2* between the two groups. The results showed that the mRNA expression levels of *FGF2* in the FGFBP1-P group were significantly up-regulated compared to the FGFBP1-O group (*P* < 0.05, Fig. 5*D*), indicating that the genotype of *FGFBP1* in the TPG and *Semnopithecus* may play an enhanced role in binding efficiency relative to other *Trachypithecus* species. Furthermore, we observed significantly higher expression levels of *ALP*, *RUNX2*, *OCN* of HJBMMSCs after osteogenic induction in the FGFBP1-P group compared to the FGFBP1-O group (*P* < 0.05, Fig. 5*E*). This implies an enhanced capacity for osteogenic differentiation in the TPG and *Semnopithecus*.

We then performed Western blot analyses to validate these findings at the protein level. There was significantly higher protein expression of FGF2 in the FGFBP1-P group compared to FGFBP1-O group (*P* < 0.05, Fig. 5*F*), which suggests the *FGFBP1* genotype in the TPG and *Semnopithecus* better protects FGF2 from degradation and promotes the expression of FGF2. The protein expressions of ALP, RUNX2, OCN in FGFBP1-P are consistent with the expression pattern from mRNA (Fig. 5*G* and *SI Appendix*, Fig. S32). In addition, alkaline phosphatase staining and alizarin red staining analyses further demonstrated that amino acid site variation in FGFBP1-P can enhance osteogenic capacity and mineralization (Fig. 5*H*).

*FOXO1*, which is reported to function in osteoblast cell differentiation (65), the mRNA and protein expression levels of ALP, RUNX2, OCN of HJBMMSCs after osteogenic induction in FOXO1-P were not significantly higher than FOXO1-O (*SI Appendix*, Figs. S31, S33, and S34). Moreover, alkaline phosphatase staining and alizarin red staining analyses also showed no significant differences in the FOXO1-P and FOXO1-O groups (*SI Appendix*, Fig. S35).

Overall, these results suggest that the FGFBP1 genotype in the TPG and *Semnopithecus* may enhance binding to FGF2, thereby promoting FGF2 expression, and up-regulating the efficiency of osteogenic differentiation and mineralization. This is likely a mechanism that contributed to the observed larger body size and changes in skull morphology in the TPG and *Semnopithecus* species compared to other *Trachypithecus* species.

## Conclusion

In this study, we observed that the TPG, which is geographically distributed in the transitional zone between other *Trachypithecus* species and *Semnopithecus*, exhibits certain morphological features that are more similar to *Semnopithecus* than other *Trachypithecus* species. Based on phylogenomic reconstructions, combined with de novo genomic assembly, we demonstrated that the TPG represents the basal clade of *Trachypithecus*. During speciation, ILS leading to shared ancestral genotypes between the TPG and *Semnopithecu*s species, contributed to phylogenetic inconsistencies in their evolutionary relationships. We found that ILS affected a set of genes with amino acid substitutions among functional domains, including the *FGFBP1*. In vitro experiments validated that these genotypes upregulate osteoblast marker genes, supporting the enhanced function of osteogenic differentiation and mineralization in the TPG, likely contributed to the similarities in skull morphology and body length between species of the TPG and *Semnopithecus*. Our study clarifies the causes of phylogenetic discordance by providing a methodological framework and quantitative evidence for the relative contributions of ILS and ancestral gene flow in shaping speciation in primates, and offers insights into the mechanisms underlying speciation and phenotypic variation in mammals.

## Materials and Methods

Additional details are in *SI Appendix*, *Materials and Methods*.

### PCA Analysis and Construction of Genomic Dataset.

We used PCA (71) to extract the main components from five morphological variables. The liver tissue sample of *T. shortridgei* from an adult male that died of natural causes. Sample collection was performed in accordance with the methods approved by the Institutional Animal Care and Use Committee at the Northwest University (NWU-AWC-20230921H). We sequenced the *T. shortridgei* genome using the MGISEQ-2000 platform, the PromethION platform, and high-throughput chromosome conformation capture (Hi-C). The de novo assembly of the *T. shortridgei* genome was performed using NextDenovo v.2.31 (72), with the Hi-C reads scaffolded to the chromosome based on the Juicer v.1.5 (73) and 3D-DNA v.180922 (74). The *T. shortridgei* genome was annotated using de novo and homology-based approaches. Whole-genome alignments were constructed with LAST v.1.04.15 (75) and MULTIZ v.11.2 (76), using *M. mulatta* as the reference. The mitochondrial genomes of five langurs were assembled using NOVOPlasty v.4.2 (77).

### Phylogeny and Demographic History Reconstruction.

One-to-one orthologs were generated from whole-genome alignment. These orthologous genes were used to generate two distinct datasets: a concatenated coding sequence alignment and fourfold degenerate sites. Conserved elements were identified using PHAST v.1.5 (78). After a false discovery rate (FDR) correction and the removal of coding region elements, CNEs were generated. For each dataset, maximum likelihood phylogenetic trees were constructed using RAxML v.8.2.9 (79). Coalescent species tree estimation was performed using ASTRAL III v.5.1.1 (41) and MP-EST v.2.0 (42) based on 1 kb windowed genes. DiscoVista v1.0 (80) was employed to quantify gene tree quartet frequencies for each major branch. Divergence times were estimated using the MCMCTREE program in PAML v.4.5 (51). Historical population dynamics were reconstructed using PSMC v.0.6.5 (44).

### Gene Flow and ILS Analyses.

Gene flow between *Trachypithecus* and *Semnopithecus* was performed using PhyloNetworks v.0.9.0, AdmixTools v.1.0.1, and *D*_FOIL_ (45, 47, 81). QuIBL (49) was used to differentiate ILS and introgression. CoalHMM (52) was utilized to build a hidden Markov model for identifying ILS regions across the whole genome. Based on the annotation file of *M. mulatta* (Mmul_10.104.gff3), we identified orthologous genes for three combinations and extracted the posterior probabilities of the coding regions from the corresponding whole genome-level CoalHMM results. GO and the KEGG pathways enrichment were conducted using KOBAS v.3.0 (57). The resulting *P*-values were corrected using the Benjamini–Hochberg method (82). Gene expression at the organ level was based on data from the Gene Expression Database (63). The functional domains of amino acid sequences were predicted using the Pfam v.1.6 database (83). Three-dimensional structural models of candidate genes were predicted using AlphaFold v2.3.1 (84).

### In Vitro Expression Assay.

The two group plasmids (FGFBP1-P and FOXO1-P representing the TPG, FGFBP1-O and FOXO1-O representing the other *Trachypithecus* species groups) were constructed by General Biol (Anhui, China). All plasmids were transfected into Human Jaw Bone Marrow Mesenchymal Stem Cells (HJBMMSCs) separately. The use of HJBMMSCs was approved by the Ethics Committee of the School of Stomatology at the Air Force Medical University (No. KQ-YJ-2025-108). Written informed consent was obtained from all participants. After 7 d of osteogenic induction, RNA extraction was carried out for subsequent qPCR analysis. On the 14th day, Western blotting was performed. On the 21st day, Alkaline phosphatase (Alp) and Alizarin Red staining procedures were implemented. Results were analyzed using GraphPad Prism. Statistical significance was set at <0.05, mean ± SD.

## Supplementary Material

Appendix 01 (PDF)

## Data Availability

The reference genome assembly of *Trachypithecus shortridgei* has been deposited (accession number: JBQWTD000000000) in the NCBI database (85). All other data are included in the manuscript and/or *SI Appendix*.

## References

[1] J. Mallet, Hybrid speciation. Nature **446**, 279–283 (2007). 17361174 10.1038/nature05706

[2] T. Zou , Uncovering the enigmatic evolution of bears in greater depth: The hybrid origin of the Asiatic black bear. Proc. Natl. Acad. Sci. U.S.A. **119**, e2120307119 (2022). 35858381 10.1073/pnas.2120307119 PMC9351369

[3] H. Wu , Hybrid origin of a primate, the gray snub-nosed monkey. Science **380**, eabl4997 (2023). 37262139 10.1126/science.abl4997

[4] B. L. Zhang , Comparative genomics reveals the hybrid origin of a macaque group. Sci. Adv. **9**, eadd3580 (2023). 37262187 10.1126/sciadv.add3580 PMC10413639

[5] S. Gavrilets, A. Vose, Dynamic patterns of adaptive radiation. Proc. Natl. Acad. Sci. U.S.A. **102**, 18040–18045 (2005). 16330783 10.1073/pnas.0506330102 PMC1312382

[6] J. C. Beehner, J. E. Phillips-Conroy, P. L. Whitten, Female testosterone, dominance rank, and aggression in an Ethiopian population of hybrid baboons. Am. J. Primatol. **67**, 101–119 (2005). 16163721 10.1002/ajp.20172

[7] J. Pastorini, A. Zaramody, D. J. Curtis, C. M. Nievergelt, N. I. Mundy, Genetic analysis of hybridization and introgression between wild mongoose and brown lemurs. BMC Evol. Biol. **9**, 32 (2009). 19196458 10.1186/1471-2148-9-32 PMC2657121

[8] W. P. Maddison, Gene trees in species trees. Syst. Biol. **46**, 3 (1997).

[9] S. Song, L. Liu, S. V. Edwards, S. Wu, Resolving conflict in eutherian mammal phylogeny using phylogenomics and the multispecies coalescent model. Proc. Natl. Acad. Sci. U.S.A. **109**, 14942–14947 (2012). 22930817 10.1073/pnas.1211733109 PMC3443116

[10] D. Zinner, M. L. Arnold, C. Roos, The strange blood: Natural hybridization in primates. Evol. Anthropol. Issues News Rev. **20**, 96–103 (2011). 10.1002/evan.20301 22034167

[11] J. Mallet, Hybridization as an invasion of the genome. Trends Ecol. Evol. **20**, 229–237 (2005). 16701374 10.1016/j.tree.2005.02.010

[12] S. A. Taylor, E. L. Larson, Insights from genomes into the evolutionary importance and prevalence of hybridization in nature. Nat. Ecol. Evol. **3**, 170–177 (2019). 30697003 10.1038/s41559-018-0777-y

[13] R. E. Green , A draft sequence of the Neandertal genome. Science **328**, 710–722 (2010). 20448178 10.1126/science.1188021 PMC5100745

[14] J. H. Degnan, N. A. Rosenberg, Gene tree discordance, phylogenetic inference and the multispecies coalescent. Trends Ecol. Evol. **24**, 332–340 (2009). 19307040 10.1016/j.tree.2009.01.009

[15] A. Scally , Insights into hominid evolution from the gorilla genome sequence. Nature **483**, 169–175 (2012). 22398555 10.1038/nature10842 PMC3303130

[16] H. Song , Scaphopoda is the sister taxon to Bivalvia: Evidence of ancient incomplete lineage sorting. Proc. Natl. Acad. Sci. U.S.A. **120**, e2302361120 (2023). 37738291 10.1073/pnas.2302361120 PMC10556646

[17] IUCN red list of threatened species. https://www.iucnredlist.org/. Deposited 10 June 2025.

[18] J. Avaria-Llautureo , The radiation and geographic expansion of primates through diverse climates. Proc. Natl. Acad. Sci. U.S.A. **122**, e2423833122 (2025). 40763018 10.1073/pnas.2423833122 PMC12358913

[19] Y. Shao , Phylogenomic analyses provide insights into primate evolution. Science **380**, 913–924 (2023). 37262173 10.1126/science.abn6919

[20] A. Estrada , Global importance of Indigenous Peoples, their lands, and knowledge systems for saving the world’s primates from extinction. Sci. Adv. **8**, eabn2927 (2022). 35947670 10.1126/sciadv.abn2927 PMC9365284

[21] C. Groves, Primate Taxonomy (Smithsonian Books, 2001).

[22] J. Rogers , The comparative genomics and complex population history of *Papio* baboons. Sci. Adv. **5**, eaau6947 (2019). 30854422 10.1126/sciadv.aau6947 PMC6401983

[23] E. F. Sorensen , Genome-wide coancestry reveals details of ancient and recent male-driven reticulation in baboons. Science **380**, eabn8153 (2023). 10.1126/science.abn8153 37262153

[24] I. Rivas-González , Pervasive incomplete lineage sorting illuminates speciation and selection in primates. Science **380**, eabn4409 (2023). 37262154 10.1126/science.abn4409

[25] R. Liedigk , Evolutionary history of the odd-nosed monkeys and the phylogenetic position of the newly described Myanmar snub-nosed monkey *Rhinopithecus strykeri*. PLoS ONE **7**, e37418 (2012). 22616004 10.1371/journal.pone.0037418 PMC3353941

[26] C. Roos , Nuclear versus mitochondrial DNA: Evidence for hybridization in colobine monkeys. BMC Evol. Biol. **11**, 77 (2011). 21435245 10.1186/1471-2148-11-77 PMC3068967

[27] Z. Liu , Genomic mechanisms of physiological and morphological adaptations of limestone langurs to karst habitats. Mol. Biol. Evol. **37**, 952–968 (2020). 31846031 10.1093/molbev/msz301

[28] X. G. Qi , Adaptations to a cold climate promoted social evolution in Asian colobine primates. Science **380**, eabl8621 (2023). 37262163 10.1126/science.abl8621

[29] N. G. Jablonski , *Mesopithecus pentelicus* from Zhaotong, China, the easternmost representative of a widespread Miocene cercopithecoid species. J. Hum. Evol. **146**, 102851 (2020). 32771770 10.1016/j.jhevol.2020.102851

[30] J. C. Mitani, J. Call, P. M. Kappeler, R. A. Palombit, J. B. Silk, Eds., The Evolution of Primate Societies (University of Chicago Press, 2012).

[31] D. Brandon-Jones , Asian primate classification. Int. J. Primatol. **25**, 97–164 (2004).

[32] C. Roos, T. Nadler, L. Walter, Mitochondrial phylogeny, taxonomy and biogeography of the silvered langur species group (*Trachypithecus cristatus*). Mol. Phylogenet. Evol. **47**, 629–636 (2008). 18406631 10.1016/j.ympev.2008.03.006

[33] J. W. Wu , First reference genome assembly of the Indochinese silvered langur (*Trachypithecus germaini*). Zool. Res. **43**, 604–607 (2022). 35726587 10.24272/j.issn.2095-8137.2022.136 PMC9336458

[34] B. Wang , Full-length numt analysis provides evidence for hybridization between the Asian colobine genera *Trachypithecus* and *Semnopithecus*: A numt clarifies a colobine hybridization event. Am. J. Primatol. **77**, 901–910 (2015). 25903086 10.1002/ajp.22419

[35] K. Arekar, A. Parigi, K. Karanth, Understanding the convoluted evolutionary history of the capped-golden langur lineage (Cercopithecidae: Colobinae). J. Genet. **100**, 79 (2021). 34787114

[36] N. Rowe, M. Myers, All the Worlds Primates (Pogonias Press, United Kingdom, 2016).

[37] K. P. Karanth, L. Singh, R. V. Collura, C.-B. Stewart, Molecular phylogeny and biogeography of langurs and leaf monkeys of South Asia (Primates: Colobinae). Mol. Phylogenet. Evol. **46**, 683–694 (2008). 18191589 10.1016/j.ympev.2007.11.026

[38] M. Osterholz, L. Walter, C. Roos, Phylogenetic position of the langur genera *Semnopithecus* and *Trachypithecus* among Asian colobines, and genus affiliations of their species groups. BMC Evol. Biol. **8**, 58 (2008). 18298809 10.1186/1471-2148-8-58 PMC2268674

[39] L. W. Cui , Distribution and conservation status of Shortridge’s capped langurs *Trachypithecus shortridgei* in China. Oryx **50**, 732–741 (2016).

[40] F. A. Simao, R. M. Waterhouse, P. Ioannidis, E. V. Kriventseva, E. M. Zdobnov, BUSCO: Assessing genome assembly and annotation completeness with single-copy orthologs. Bioinformatics **31**, 3210–3212 (2015). 26059717 10.1093/bioinformatics/btv351

[41] C. Zhang, M. Rabiee, E. Sayyari, S. Mirarab, ASTRAL-III: Polynomial time species tree reconstruction from partially resolved gene trees. BMC Bioinf. **19**, 153 (2018). 10.1186/s12859-018-2129-y PMC5998893 29745866

[42] L. Liu, L. Yu, S. V. Edwards, A maximum pseudo-likelihood approach for estimating species trees under the coalescent model. BMC Evol. Biol. **10**, 302 (2010). 20937096 10.1186/1471-2148-10-302 PMC2976751

[43] I. Miura, F. Shams, T. Ezaz, M. Ogata, One-step leaping evolution from an autosomal pair to the heteromorphic sex chromosomes. Sex. Dev. **18**, 61–69 (2024). 39522502 10.1159/000542537

[44] H. Li, R. Durbin, Inference of human population history from individual whole-genome sequences. Nature **475**, 493–496 (2011). 21753753 10.1038/nature10231 PMC3154645

[45] C. Solis-Lemus, P. Bastide, C. Ane, Phylonetworks: A package for phylogenetic networks. Mol. Biol. Evol. **34**, 3292–3298 (2017). 28961984 10.1093/molbev/msx235

[46] Y. Moodley , Interspecific gene flow and the evolution of specialization in black and white rhinoceros. Mol. Biol. Evol. **37**, 3105–3117 (2020). 32585004 10.1093/molbev/msaa148

[47] J. B. Pease, M. W. Hahn, Detection and polarization of introgression in a five-taxon phylogeny. Syst. Biol. **64**, 651–662 (2015). 25888025 10.1093/sysbio/syv023

[48] B. Pfeifer, D. D. Kapan, Estimates of introgression as a function of pairwise distances. BMC Bioinf. **20**, 207 (2019). 10.1186/s12859-019-2747-z PMC6480520 31014244

[49] N. B. Edelman , Genomic architecture and introgression shape a butterfly radiation. Science **366**, 594–599 (2019). 31672890 10.1126/science.aaw2090 PMC7197882

[50] S. Feng , Incomplete lineage sorting and phenotypic evolution in marsupials. Cell **185**, 1646–1660.e18 (2022). 35447073 10.1016/j.cell.2022.03.034 PMC9200472

[51] Z. Yang, PAML: A program package for phylogenetic analysis by maximum likelihood. Bioinformatics **13**, 555–556 (1997). 10.1093/bioinformatics/13.5.555 9367129

[52] A. Hobolth, J. Y. Dutheil, J. Hawks, M. H. Schierup, T. Mailund, Incomplete lineage sorting patterns among human, chimpanzee, and orangutan suggest recent orangutan speciation and widespread selection. Genome Res. **21**, 349–356 (2011). 21270173 10.1101/gr.114751.110 PMC3044849

[53] K. Munch, K. Nam, M. H. Schierup, T. Mailund, Selective sweeps across twenty millions years of primate evolution. Mol. Biol. Evol. **33**, 3065–3074 (2016). 27660295 10.1093/molbev/msw199

[54] Y. T. Guo , Evolution of sexual dimorphism in nonhuman primates. Sci. China Life Sci. **68**, 2472–2474 (2025). 40106186 10.1007/s11427-024-2796-3

[55] T. H. Webster, M. A. Wilson Sayres, Genomic signatures of sex-biased demography: Progress and prospects. Curr. Opin. Genet. Dev. **41**, 62–71 (2016). 27599147 10.1016/j.gde.2016.08.002

[56] F. L. Mendez, Differences in the effective population sizes of males and females do not require differences in their distribution of offspring number. Theor. Popul. Biol. **114**, 19–28 (2017). 27915040 10.1016/j.tpb.2016.11.002

[57] C. Xie , KOBAS 2.0: A web server for annotation and identification of enriched pathways and diseases. Nucleic Acids Res. **39**, W316–W322 (2011). 21715386 10.1093/nar/gkr483 PMC3125809

[58] A. Cagan , Natural selection in the great apes. Mol. Biol. Evol. **33**, 3268–3283 (2016). 27795229 10.1093/molbev/msw215 PMC5100057

[59] L. Yanan, L. Hui, C. Zhuo, D. Longqing, S. Ran, Comprehensive analysis of mitophagy in HPV-related head and neck squamous cell carcinoma. Sci. Rep. **13**, 7480 (2023). 37161060 10.1038/s41598-023-34698-4 PMC10170109

[60] K. W. Wucherpfennig, E. Gagnon, M. J. Call, E. S. Huseby, M. E. Call, Structural biology of the t-cell receptor: Insights into receptor assembly, ligand recognition, and initiation of signaling. Cold Spring Harb. Perspect. Biol. **2**, a005140 (2010). 20452950 10.1101/cshperspect.a005140 PMC2845206

[61] B. Malnic, P. A. Godfrey, L. B. Buck, The human olfactory receptor gene family. Proc. Natl. Acad. Sci. U.S.A. **101**, 2584–2589 (2004). 14983052 10.1073/pnas.0307882100 PMC356993

[62] L. Zhao , Dynamic foraging strategy adaptation to heterogeneous environments contributes to social aggregation in snub-nosed monkeys. Zool. Res. **45**, 39–54 (2024). 38114432 10.24272/j.issn.2095-8137.2023.047 PMC10839657

[63] C. M. Smith , The mouse Gene Expression Database (GXD): 2019 update. Nucleic Acids Res. **47**, D427–D432 (2019). 30335138 10.1093/nar/gky922 PMC6324054

[64] J. R. Gilbert , Genetic associations and phenotypic heterogeneity in the craniosynostotic rabbit. PLoS ONE **13**, e0204086 (2018). 30235265 10.1371/journal.pone.0204086 PMC6147457

[65] P. Chen , Scara3 regulates bone marrow mesenchymal stem cell fate switch between osteoblasts and adipocytes by promoting Foxo1. Cell Prolif. **54**, e13095 (2021). 34254370 10.1111/cpr.13095 PMC8349663

[66] E. Tassi , Enhancement of fibroblast growth factor (FGF) activity by an FGF-binding protein. J. Biol. Chem. **276**, 40247–40253 (2001). 11509569 10.1074/jbc.M104933200

[67] M. G. Sabbieti , Endogenous FGF-2 is critically important in PTH anabolic effects on bone. J. Cell. Physiol. **219**, 143–151 (2009). 19107841 10.1002/jcp.21661 PMC2763338

[68] T. Naganawa , Reduced expression and function of bone morphogenetic protein-2 in bones of *Fgf2* null mice. J. Cell. Biochem. **103**, 1975–1988 (2008). 17955502 10.1002/jcb.21589

[69] W. Zhang , The expression and prognostic value of FGF2, FGFR3, and FGFBP1 in esophageal squamous cell carcinoma. Anal. Cell. Pathol. **2020**, 1–17 (2020). 10.1155/2020/2872479 PMC7748917 33381388

[70] J. S. Park, D. Kim, H. S. Hong, Priming with a combination of FGF2 and HGF restores the impaired osteogenic differentiation of adipose-derived stem cells. Cells **11**, 2042 (2022). 35805126 10.3390/cells11132042 PMC9265418

[71] J. Lever, M. Krzywinski, N. Altman, Principal component analysis. Nat. Methods **14**, 641–642 (2017).

[72] J. Hu , Nextdenovo: An efficient error correction and accurate assembly tool for noisy long reads. Genome Biol. **25**, 107 (2024). 38671502 10.1186/s13059-024-03252-4 PMC11046930

[73] N. C. Durand , Juicer provides a one-click system for analyzing loop-resolution Hi-C experiments. Cell Syst. **3**, 95–98 (2016). 27467249 10.1016/j.cels.2016.07.002 PMC5846465

[74] O. Dudchenko , De novo assembly of the *Aedes aegypti* genome using Hi-C yields chromosome-length scaffolds. Science **356**, 92–95 (2017). 28336562 10.1126/science.aal3327 PMC5635820

[75] S. M. Kiełbasa, R. Wan, K. Sato, P. Horton, M. C. Frith, Adaptive seeds tame genomic sequence comparison. Genome Res. **21**, 487–493 (2011). 21209072 10.1101/gr.113985.110 PMC3044862

[76] M. Blanchette , Aligning multiple genomic sequences with the threaded blockset aligner. Genome Res. **14**, 708–715 (2004). 15060014 10.1101/gr.1933104 PMC383317

[77] N. Dierckxsens, P. Mardulyn, G. Smits, NOVOPlasty: De novo assembly of organelle genomes from whole genome data. Nucleic Acids Res. **45**, e18 (2016). 10.1093/nar/gkw955 PMC5389512 28204566

[78] M. J. Hubisz, K. S. Pollard, A. Siepel, PHAST and RPHAST: Phylogenetic analysis with space/time models. Brief. Bioinf. **12**, 41–51 (2011). 10.1093/bib/bbq072 PMC3030812 21278375

[79] A. Stamatakis, RAxML version 8: A tool for phylogenetic analysis and post-analysis of large phylogenies. Bioinformatics **30**, 1312–1313 (2014). 24451623 10.1093/bioinformatics/btu033 PMC3998144

[80] E. Sayyari, J. B. Whitfield, S. Mirarab, Discovista: Interpretable visualizations of gene tree discordance. Mol. Phylogenet. Evol. **122**, 110–115 (2018). 29421312 10.1016/j.ympev.2018.01.019

[81] N. Patterson , Ancient admixture in human history. Genetics **192**, 1065–1093 (2012). 22960212 10.1534/genetics.112.145037 PMC3522152

[82] Y. Benjamini, Y. Hochberg, Controlling the false discovery rate: A practical and powerful approach to multiple testing. J. R. Stat. Soc. Ser. B. Methodol. **57**, 289–300 (1995).

[83] S. El-Gebali , The Pfam protein families database in 2019. Nucleic Acids Res. **47**, D427–D432 (2019). 30357350 10.1093/nar/gky995 PMC6324024

[84] J. Jumper , Highly accurate protein structure prediction with AlphaFold. Nature **596**, 583–589 (2021). 34265844 10.1038/s41586-021-03819-2 PMC8371605

[85] X. G. Qi , Incomplete lineage sorting shaped mixed traits during a colobine primate radiation. National Center for Biotechnology Information. https://www.ncbi.nlm.nih.gov/nuccore/JBQWTD000000000. Deposited 1 December 2025. 10.1073/pnas.2524833123 PMC12867756 41576102

