# Supplementary material for: Incomplete lineage sorting shaped mixed traits during a colobine primate radiation
Source: Proc Natl Acad Sci U S A. 2026 Jan 23;123(5):e2524833123. doi: 10.1073/pnas.2524833123 (PMC12867756; doi:10.1073/pnas.2524833123)
Supplement: Supplementary file 1 — Appendix 01 (PDF) [file pnas.2524833123.sapp.pdf]

Main Manuscript for

Incomplete lineage sorting shaped mixed traits during a Colobine primate radiation

Yan-Qing Guo[a,1], Yiming Wang[b,1], Paul A. Garber[c,d], Yingchun Li[e,f], Ru Zhang[a], Chi Zhang[g], Zhipang Huang[d], Dong-Dong Wu[h], Bao-Guo Li[a], Liangwei Cui[f,*], Bei Li[b,*], Xiao-Guang Qi[a,*]

[a] College of Life Sciences, Northwest University, Xi'an, 710069, China.

[b] School of Stomatology, Air Force Medical University, Xi'an, 710032, China.

[c] Department of Anthropology, University of Illinois, Urbana, 61820, USA.

[d] International Centre of Biodiversity and Primate Conservation, Dali University, Dali, 671003, China.

[e] Gaoligong Mountain Forest Ecosystem Research Station, Kunming Institute of Botany, Kunming, 650222, China.

[f] Forestry college, Southwest Forestry University, Kunming, 650224, China.

[g] BGI, Shenzhen, 518083, China.

[h] Kunming Institute of Zoology, Chinese Academy of Sciences, Kunming, 650201, China.

[1]Yan-Qing Guo and Yiming Wang equally to this work.

***Correspondence:** Xiao-Guang Qi, Bei Li, Liang-Wei Cui

Email: qixg@nwu.edu.cn, libei2021@fmmu.edu.cn, cuilw@eastern-himalaya.cn

This PDF file includes:

Supplementary Methods

Figures S1 to S35

Tables S1 to S27

SI References

1 Materials and Methods

1.1 Geographical Distribution Information

The distribution of species of *Semnopithecus* and *Trachypithecus* were obtained from the IUCN (www.iucnredlist.org, 2025).

1.2 Statistical Analysis of Morphological Characters

We collected measurements including total skull length, condylobasal length, zygomatic width, body length, and tail length from adult male specimens across 12 primate species (1–4) (Table S1). All measurements were standardized and tested for a normal distribution prior to analysis (Fig. S1). We then performed the Kaiser-Meyer-Olkin (KMO) test and Bartlett's test to assess the suitability of the data for dimensionality reduction. The coefficient of the KMO test was 0.71 (>0.6), and Bartlett's test showed a P value of 1.611045e-170 (<0.05), indicating the correlation matrix was appropriate for PCA analysis. Subsequently, we used Principal Components Analysis (PCA) (5) to extract components from all 5 morphological variables, utilizing the "principal" function in the R package *psych* (6) to extract principal components, and to assess the contribution of each measurement to these components. Given the high correlations among morphological features, we performed factor rotation and subsequently conducted a new PCA to compare the results with the initial unrotated solution. To account for potential instability arising from limited sample sizes, a hierarchical clustering analysis (7) (Euclidean distance metric with Ward's linkage method) was performed in R (v 4.0.5), and the results were visualized as a dendrogram. Additionally, we used the first two principal component axes to perform the model-based clustering analysis using the R package *mclust* (8), with the Bayesian Information Criterion (BIC) identifying two clusters as the optimal solution. If the clustering validation score exceeded 0.5, we assumed that the observed group structure was robust. Based on the PCA results, an ANOVA was conducted among the three predefined groups (*Trachypithecus, Semnopithecus* and *T. pileatus* group) to assess differences in PC1 scores.

1.3 Sample Information, Genome Sequencing, Assembly, and Annotation

We included the genomes of 12 primate species in this study, of which one was newly sequenced. The rhesus macaque (*Macaca mulatta*) was used as an overall outgroup. These remaining 10 species were Asian colobines, including four species of classical langurs and six species of odd-nosed monkeys. Reference genomes of *Trachypithecus francoisi* (GenBank: GCA_009764315.1), *Rhinopithecus bieti*

(GenBank: GCA_001698545.2), *R. strykeri* (GenBank: GCA_023764705.1), and *M. mulatta* (GenBank: GCA_003339765.3) were downloaded from NCBI. *Nasalis larvatus*, *Pygathrix nigripes, Simias concolor*, *R. roxellana*, *T. germaini*, *T. crepusculus*, and *S. entellus* genome sequences were derived from the Primates Genome Project (9).

Fresh liver samples of a male *T. shortridgei* from the Gaoligongshan National Nature Reserve, Yunnan Province, China, which died of natural causes, were analyzed. Sample collection was performed in accordance with the methods approved by the Institutional Animal Care and Use Committee at the Northwest University (NWU-AWC-20230921H). Genomic DNA for *T. shortridgei* were extracted from liver tissue using a DNeasy Blood &Tissue Kit (QIAGEN). A short-insert-size library (350 bp) was constructed and sequenced using the MGISEQ-2000 platform with 150 bp paired-end sequencing. For each Nanopore library, gDNA was size-selected (>20 kb) with a Blue Pippin (Sage Science, Beverly, MA) and sequenced using the PromethION platform (Oxford Nanopore, Oxford, UK) according to the manufacturer's instructions. Before starting the assembly, we used a *k-mer* analysis (10) to estimate the genome size and heterozygosity of *T. shortridgei*. Then, we used NextDenovo (11) software for genome *de novo* assembly. We used Nextpolish software (12) to combine the nanopore reads with all short reads. To improve genome assembly quality, two rounds of polishing were executed by Plion (13) using the short reads with the "fix-all" mode to achieve the final genome. To generate a chromosome-scale genome, the contigs were anchored into chromosomes by Hi-C sequencing reads using the Juicer (14) and 3D-DNA (15) software. Finally, short clean reads and Benchmarking Universal Single-Copy Orthologs (BUSCO) (16) were used to evaluate assembly genome completeness, referring to the mammalia_odb10 BUSCO set. All of the short clean reads (350 bp) were mapped onto the assembled genome using BWA (17) with default settings. The mapping rate was counted using Samtools software (18).

Repetitive sequences were identified using *de novo* and homology-based approaches. For *de novo* prediction, we used RepeatModeler (19) to predict repetitive sequences and classified them using Repbase. For homology-based annotation, we used RepeatMasker (19) with Repbase TE library to identify DNA-level TEs, and RepeatProteinMask (19) with parameters (-no LowSimple -pvalue 1e-04) to identify protein-level TEs. Additionally, the Tandem Repeat Finder (TRF) program (20) was used to determine tandem repeats. A combination of *de novo* and homology-based approach was applied to predict the structures of protein-coding genes. For *de novo* gene prediction, we utilized SNAP (version 2006-07-28) (21), GlimmerHMM

(version 3.0.3) (22), and AUGUSTUS (version 2.5.5) (23) to analyze the *T. shortridgei* genome. For homology-based prediction, the protein sequences of *Homo sapiens* (GenBank: GCA_000001405.29), *R. roxellana* (GenBank: GCA_007565055.1) and *T. francoisi* (GenBank: GCA_009764315.1) were mapped to *T. shortridgei* genomes using TBLASTN (24) with an E-value cut-off of 1e-5. Then, a non-redundant consensus gene set was generated based on the predictions of the EVidenceModeler software (25). Short-length (< 50 amino acids) and prematurely terminating genes were removed from the consensus gene set. Finally, to assess gene completeness and structural features, BUSCO was used to evaluate the quality of gene annotation and to compare the gene structure features of closely related published species to determine the reliability of the gene annotation. These gene structure features include average mRNA length, average CDS length, average exon length, average intron length, and the number of exons.

1.4 Phylogenetic tree construction

1.4.1 Whole Genome Alignment

We obtained pairwise whole-genome alignments (WGAs) between genomes of the *M. mulatta* and 11 species of Asian colobines by using LAST software (26) with parameters: lastal -P 20 -m 50 -E 0.05. We used the "maf-swap" command to reorder and filter the sequences to obtain the optimal alignment blocks. We used MULTIZ (27) to merge the pairwise alignments into multiple genome alignments using the *M. mulatta* genome as the reference. Sequences with low mapping quality (< 100bp) were excluded using MafFilter (28).

1.4.2 Orthologous gene trees

The multiple genome alignments of the 11 species of Asian colobines were used to identify the orthologous genes of each species. The consensus coding sequences of each species were extracted based on the annotation file of *M. mulatta* (*Macaca_mulatta*.Mmul_10.104.gff3). The one-to-one orthologous genes were concatenated to generate a supergene sequence, which was used for phylogenetic tree construction. Orthologous gene trees were constructed using RAxML (29) with 200 bootstrap replicates under the GTR+gamma model. The four-fold degenerate (4d) sites of one-to-one orthologous genes were extracted using inhouse Perl scripts and concatenated for constructing an ML phylogenetic tree using RAxML with 200 bootstrap replicates under the GTR+gamma model.

1.4.3 Conserved Non-coding Element (CNE) trees

In addition, we chose *M. mulatta* as an outgroup to identify conservative elements from the 11 Asian colobine species. We used the phyloFit program in the PHAST package (30) with fourfold degenerate sites (4dTV) and the topology to generate non-conservative models. The non-conservative models for each chromosome were integrated using phyloBoot to produce an average non-conservative model. Subsequently, the MAF file was divided into 5 kb fragments using the msa_split tool. Conservative models for each fragment were generated using PhastCons (31). All conservative models were integrated using the phyloBoot software to create an average conservative model. Next, the PhastCons (with the parameter phastCons --estimate-rho) was used to generate conserved elements corresponding to each chromosome based on the obtained non-conservative and conservative models, along with conservative scores. Conserved elements were filtered based on a *p*-value threshold less than 0.05 and were identified using a threshold of $P < 0.05$ (corrected *P* value). This was applied to a False Discovery Rate (FDR) correction in R. Conserved elements located in coding regions were filtered out according to the genome annotation file of *M. mulatta*. Finally, conserved non-coding element trees were constructed using RAxML with 200 bootstrap replicates based on the GTR+gamma model.

1.4.4 Mitochondrial genome trees

The mitochondrial genomes of five langurs (*T. shortridgei, S. entellus, T. germaini, Nasalis larvatus*, *Rhinopithecus roxellana)* were assembled using NOVOPlasty (32) with default settings. We utilized and integrated all previously published langur genomes (n=7) from Genebank (NC_018059.1, NC_008215.1, NC_020667.1, NC_008216.1, NC_008218.1, NC_023970.1, NC_015486.1). Mitochondrial coding sequence alignments were aligned with MUSCLE (v3.7) (33) using default settings. Mitochondrial genome trees were constructed using RAxML with 200 bootstrap replicates under the GTR+gamma model.

1.4.5 Species trees

To further assess discordance among gene trees, we extracted 200bp, 1 Kb, 10 Kb, 50 Kb window gene sequences, at least 50 Kb distant from each other in genome, using a sliding window approach. We constructed 200bp, 1 Kb, 10 Kb, 50 Kb window gene trees by RAxML under the GTR+gamma model separately and plotted 1Kb window gene trees using Densitree V2.2.7 (34), filtered the resulting trees with probabilities of less than 25% to minimize the negative impacts of noise. Then, we applied ASTRAL-III (35) to reconstruct the species tree from 1 Kb window gene trees

using the default parameters. We also used another coalescent-based phylogenetic method, MP-EST (36), to infer the species tree. In addition, we also extracted 200bp, 1Kb, 10Kb, 50Kb window gene trees from the X chromosome, and reconstructed the species tree from 1 Kb window gene tree using ASTRAL-III and MP-EST. Moreover, we evaluated the quartet frequencies of each major and controversial branch, based on all chromosomes and the X chromosome respectively, using DiscoVista (37) with the parameters: "-k 1 txt -m 5".

1.5 Populations Demographic History

1.5.1 Divergence Time

Divergence times were estimated using the MCMCTree program in PAML v4.5 (38) with the topology of the species tree, 4dTV site alignments, and fossil calibration time points. Fossil data from published studies (39–42) were utilized for divergence time calibration: 1) between the Cercopithecinae and the Colobinae: 12.5-19 Mya. 2) between the classical langurs and the odd-nosed monkeys: 6.7-8.0 Mya. 3) between *Trachypithecus* and *Semnopithecus*: 3.2-5.7 Mya. 4) Origin time of *Rhinopithecus*: 5.7-7.0 Mya. This process was repeated twice to assess stability. In addition, we estimated divergence times for two datasets: 1) Extract 10 Kb window gene sequences supporting the species tree topology. 2) Extract 10 Kb window gene sequences supporting *T. shortridgei* and *S. entellus* as sister clades.

1.5.2 Demographic History Reconstruction

The population dynamics history of *T. shortridgei*, *T. francoisi*, *T. germaini*, *T. crepusculus*, and *S. entellus* was simulated using PSMC (Pairwise Sequentially Markovian Coalescent) (43). Initially, we used BWA software (44) to generate bam files for paired end reads. Subsequently, SAMtools software (45) was employed (with parameters: mpileup -q 20 -Q 20) to identify SNP sites. Next, SNP genotypes were filtered based on alignment coverage being less than half or more than double the average depth. Lastly, a PSMC analysis was conducted to infer the history of population changes. Generation time was considered as 12 years, and we employed a neutral mutation rate of (μ) = $1.0\text{x}10^{-8}$. To ensure reliability, each PSMC test was examined with 100 bootstrap replicates.

1.6 Gene Flow and ILS Analyses

1.6.1 PhyloNetworks Analyses

We utilized the snaq program in the PhyloNetworks software (46) to estimate potential hybridization events, gene flow direction, and the contribution proportion of hybridization nodes. The maximum number of hybridization events in the simulated network was set to six (ranging from hmax=0 to hmax=6). To ensure accuracy, each case was simulated 100 times. The simulation results were evaluated using the -log score, where lower scores indicate better fit.

1.6.2 *D*-statistics analysis

We used the "qpDstat" command in AdmixTools (47) for *D*-statistics analysis, *M. mulatta* was used as an outgroup, and all possible four-taxon topologies of *S. entellus*, *T. shortridgei*, *T. francoisi*, *T. germaini*, *T. crepusculus* were analyzed. *D*-statistics determine whether gene flow exists between taxon P3 and P2 or P1 by calculating the difference between the number of ABBA and BABA pattern sites and the sum of pattern sites for both patterns (48). The significance of the *D*-statistic was accessed by performing Weighted Block Jack-knife methods. If the absolute value of Z was greater than 3, significant gene flow between taxa is indicated. We calculated *D*-values for species pairs using P2 and P3 as a pair if *D*-values for the triplet was significant and positive, and P3 and P1 as a pair if *D*-values for the triplet was significant and negative (48).

We used *M. mulatta* as the outgroup and tested four and five taxa: *S. entellus*, *T. shortridgei*, *T. francoisi*, *T. germaini*, *T. crepusculus*. The alignment sequences were subsequently divided into 10 kb and 100 kb window genes respectively, and filtered by the ratio (50%) of species that had an effective length ratio (80%). We primarily based our conclusions on the 100 kb window size, as it has been suggested to be sufficiently large to minimize the proportion of false positives (49). A complementary analysis using a 10 kb window was conducted to assess the consistency of the results across different windows sizes. After filtering, the windows were formatted according to the requirements of the D_{FOIL} software and subjected to statistical analysis. We filtered the windows based on the minimum total number of sites (>1,000) and minimum number of site counts for any of the D_{FOIL} components (>100) per window. We used a v2 goodness-of-fit test with a cutoff of $P<0.001$ to determine the significance of the inferred introgression signal (49).

Assuming recent gene flow between *T. shortridge* and *S. entellus*, we expected to observe relatively longer shared genomic segments than those resulting from ancestral gene flow and/or ILS, as recombination continuously fragments these segments over time (50). We then estimated the number of consecutive windows showing a significant signal of gene flow for *S. entellus*, *T. shortridgei.* Furthermore, these gene

flow windows between *T. shortridgei* and *S. entellus* were detected in discrete, non-contiguous genomic segments, based on 100 kb. The majority (88.51-87.51%) of gene flow windows between *T. shortridgei* and *S. entellus* occurred as discrete, non-contiguous segments, with only 11.49-12.49% organized in blocks of two to five consecutive windows based on 10 kb windows (*SI Appendix*, Fig. S19 and Table S17). The results indicated that introgressed genomic regions were predominantly short in length.

1.6.3 QuIBL analysis

We employed QuIBL (51) to discern whether the observed phylogenetic discordances were better explained by ancient gene flow or ILS. The QuIBL method using a likelihood-based framework to differentiate two processes from gene flow and ILS. For each process, the method calculates the likelihood of observing the internal branch length in multiple topological trees by the Bayesian Information Criterion (BIC). If only ILS is present, the distribution of internal branch lengths will be more in line with the expected ILS distribution rather than the expected gene flow distribution. In this study, three triplets were tested: 1) *T. shortridgei-T. francoisi-S. entellus* (Tsho-Tfra-Sent); 2) *T. shortridgei-T. germaini-S. entellus* (Tsho-Tger-Sent); 3) *T. shortridgei-T. crepusculus-S. entellus* (Tsho-Tcre-Sent); 4) *T. shortridgei-T. francoisi-T. crepusculus* (Tsho-Tfra-Tcre); 5) *T. shortridgei-T. francoisi- T. germaini* (Tsho-Tfra-Tger); 6) *T. shortridgei-T. crepusculus-T. germaini* (Tsho-Tcre-Tger). The *R* internal script was used to extract the corresponding species from the 10 kb window gene trees, which were separated by 200 kb windows. These randomly selected gene trees were then taken as input files for the QuIBL analysis, and this process was repeated 100 times. The BIC values were computed for situations where only ILS (Scenario 1) or a mixture of ILS and gene flow (Scenario 2) were present. If the difference between the BIC value of Scenario 2 and Scenario 1 was greater than 10, it indicated the presence of only ILS. Conversely, if the difference was less than 10, it suggested a combination of ILS and gene flow as the most likely scenario for the internal branch mixture.

1.6.4 CoalHMM analysis

We utilized CoalHMM software (52) to build a hidden Markov model to identify an ILS signal across the whole genome. The alignments of species were divided into 1Mb windows. Randomly selected window sequences were used for parameter testing. The CoalHMM program was run using the unlock model, setting values for tau1, tau2, theta1, and theta2, in order to compute the optimal model parameter values. Based on

the simulated results, these values were considered as the best parameters, and CoalHMM analysis was performed on all window sequences. Finally, using posterior probabilities, the most likely lineage situation was determined for each site. For each site, four scenarios were assumed: 1) type 0, no ILS, consistent with the species tree topology; 2) type 1, no ILS, but with longer branches consistent with the species tree topology; 3) type 2, ILS, the other three species of *Trachypithecus* were more closer to *S. entellus* than to *T. shortridgei* (Tgre-Sent, Tfra-Sent, Tcre-Sent); 4) type 3, ILS, *T. shortridgei* was closer to *S. entellus* than any of the other three species of *Trachypithecus* (Tsho-Sent). The lineage situation for each site was determined based on the highest posterior probability. To identify ILS segments, we defined the ILS signal of each site based on the positional information within the identified windows. We then exacted contiguous ILS segments by merging consecutive sites exhibiting ILS signals. Finally, we annotated the genomic coordinates of all retained ILS segments relative to the reference genome of *M. mulatta* for subsequent functional analysis. This analysis was carried out for three possible quartet combinations:
Combination 1: *T. germaini-T. shortridgei-S. entellus-M. mulatta*;
Combination 2: *T. francoisi-T. shortridgei-S. entellus-M. mulatta*;
Combination 3: *T. crepusculus-T. shortridgei-S. entellus-M. mulatta*.

1.7 ILS Candidate Gene Identification

Based on the *M. mulatta* annotation file, we identify orthologous genes for three combinations and extracted the posterior probabilities of the coding regions from the corresponding whole genome-level CoalHMM results. For each orthologous gene, if the site numbers of type 3 were highest among the sites counted, the gene was considered to be an ILS gene (type 3). Furthermore, only those orthologous genes with a total number of extracted sites exceeding 30% of the coding region length were retained for further analysis. Then, RAxML software was used to calculate the likelihood values for each alignment. The topology with the highest likelihood value was considered as the best tree. A gene was classified as an ILS candidate orthologous gene only if the best topology assigned by RAxML was the same as the ILS type assigned by CoalHMM. Gene Ontology (GO) enrichment and Kyoto Encyclopedia of Genes and Genomes (KEGG) pathway enrichment analysis using KOBAS (53). The obtained *p*-values were corrected using the Benjamini-Hochberg method (54), where pathways or GO terms with a false discovery rate (FDR) less than 0.1 were considered significantly enriched. Finally, we used Python scripts to perform specific site mutation analysis on key genes and retrieved amino acid sites shared between *T. shortridgei* and *S. entellus,* but differing from other *Trachypithecus*

species. These sites were also compared with those of other primate species, the genomic data of *S. hypoleucos* (GenBank: GCA_963573665.1), *S. priam* (GenBank: GCA_963574295.1), *S. schistaceus* (GenBank: GCA_963574385.1), *T. pileatus* (GenBank: GCA_963573805.1), and *T. geei* (GenBank: GCA_963573545.1) obtained from the NCBI were excluded from the whole-genome analysis due to low genome completeness (BUSCO evaluation results all below 50%). Only the amino acid sequences of key genes were aligned for comparison. The gene sequence data for the remaining primate species were all sourced from previous studies under the Primate Genome Project (9). We assessed the gene expression at the organ level based on data from the Gene Expression Database (55). We predicted the functional domains of amino acid sequences using the Pfam database (56) to determine whether these specific amino acid changes were located within important functional domains.

1.8 Protein 3D structure simulation

The sequence of *FGFBP1* and *FOXO1* genes were extracted in *T. shortridgei*, and named FGFBP1-P and FOXO1-P. Subsequently, we generated the sequence of FGFBP1-O and FOXO1-O by artificially replacing the ILS signal site mutations found in FGFBP1-P and FOXO1-P sequence with those present in the other *Trachypithecus* species. Tree-dimensional structural models of FGFBP1-P, FGFBP1-O, FOXO1-P and FOXO1-O protein were predicted using AlphaFold2 (57), then visualized using Pymol (https://pymol.org/). Root Mean Square Deviation (RMSD) of the predicted 3D structures were calculated using PyMOL to analyze the similarity between two proteins.

1.9 In vitro expression assay

1.9.1 Cell cultures and Plasmids transfection

Human Jaw Bone Marrow Mesenchymal Stem Cells (HJBMMSCs) obtained from three donors was approved by the Ethics Committee of the School of Stomatology at the Air Force Medical University (Approval No. KQ-YJ-2025-108). All personal identifiers were removed and replaced with coded identifiers to protect participant privacy. The study adhered to the ethical principles outlined in the 1975 Declaration of Helsinki. All procedures adhered to the ethical principles of the Declaration of Helsinki. Informed written consent was obtained from all participants after they were fully advised of the study's purpose and potential risks.

HJBMMSCs were cultured in alpha Minimum essential medium (α-MEM) (Gibco, USA) supplemented with 10% fetal bovine serum (FBS) (Gibco, USA), 2 mM L-glutamine, 100 U/mL penicillin, and 100 g/mL streptomycin (all from

Invitrogen, USA) at 37°C with 5% CO_2. Every 3 days, the cultured medium was replenished. Passage 3 to 6 cells were used for all experiments. FGFBP1-P, FOXO1-P, FGFBP1-O and FOXO1-O sequence were cloned into PCDNA3.1-EGFP vector separately by the company General Biol located in Anhui Province, China. The plasmids were divided into two groups: FGFBP1-P and FOXO1-P representing the TPG group, FGFBP1-O and FOXO1-O representing the other *Trachypithecus* species groups. Following amplification, the plasmids were extracted using the endotoxin-free plasmid extraction kit (TIANGEN, DP117, China). The plasmid DNA was quantified before use. Plasmid transfection was carried out using the X-tremeGENE HP DNA Transfection Reagent (Roche, 06366546001, Switzerland). When the cell confluence reached 80% in the culture dish, transfection was performed at a ratio of 3 μg plasmid DNA: 1μl transfection reagent. The transfection procedures were strictly in accordance with the instruction manual and the transfection result was detected by a Confocal Microscope.

1.9.2 Osteogenic Induction, Quantitative Real Time PCR and Western Blotting

After plasmid transfection, the original medium was removed, washed with pre-warmed PBS, and the osteogenic induction medium (α-MEM with 10% FBS, 10 mM β-glycerophosphate sodium, 50 μg/ml ascorbic acid and 100 nM dexamethasone) was added. The sample was incubated for 21 days and replaced every 2 - 3 days. On the 7th day of induction, RNA extraction was carried out for subsequent qPCR analysis. On the 14th day, Western blotting was performed. On the 21st day Alkaline phosphatase (Alp) and Alizarin Red staining procedures were implemented.

Total RNA was extracted with a TRIzol reagent (15–596-026, Invitrogen, USA) and converted to cDNA using PrimeScript RT Master Mix Kit (RR036A, Takara, Japan). Then, a quantitative reverse transcription-polymerase chain reaction (qRTPCR) was conducted with TB Green Premix Ex Taq II (RB820A, Takara, Japan) using the qRT-PCR System (Bio-Rad, USA). The relative expression levels of fibroblast growth factor 2 (FGF2), Alkaline phosphatase (ALP), Runt-related transcription factor 2 (RUNX2), and Osteocalcin (OCN) were calculated using the $2^{-\Delta\Delta Ct}$ method for statistical analysis. The expression was normalized to the average expression of all control individuals. The housekeeping gene used was GAPDH. Information on primers is indicated in Table S19.

Proteins were extracted with a RIPA buffer containing protease inhibitors (Beyotime, China). All proteins were loaded onto sodium dodecyl sulfate-polyacrylamide (SDS) gels and were transferred to polyvinylidene fluoride (PVDF) membranes following BCA quantification (Milipoll, USA). The membranes were

blocked with 5% bovine serum albumin for 2 h at room temperature, treated overnight at 4°C with primary antibodies, and then incubated for 2 h at room temperature with peroxidase-conjugated secondary antibodies (CWBio, China). Protein bands were detected using an imaging system (Tanon, China) and quantified with Image J software. The main antibodies included GAPDH (CWBio, CW0100, China), ALP (Abcam, ab229126, UK), RUNX2 (Abcam, ab236639, UK), Collagen I (Abcam, ab260043, UK), and FGF2 (Abcam, ab208687, UK).

1.9.3 ALP and Alizarin Red Staining

Alizarin Red and ALP powder was dissolved in distilled water at a concentration of 0.01g/ml respectively. Each solution was filtered through a 0.22 μm filter membrane and stored at room temperature for later use. After 21 days of osteogenic induction, the HJBMMSCs were fixed with 4% paraformaldehyde and then stained by Alizarin Red and an ALP staining solution. The stained cells were photographed after being washed. The percentage of the positively stained area was analyzed with Image J software.

Figures and Tables

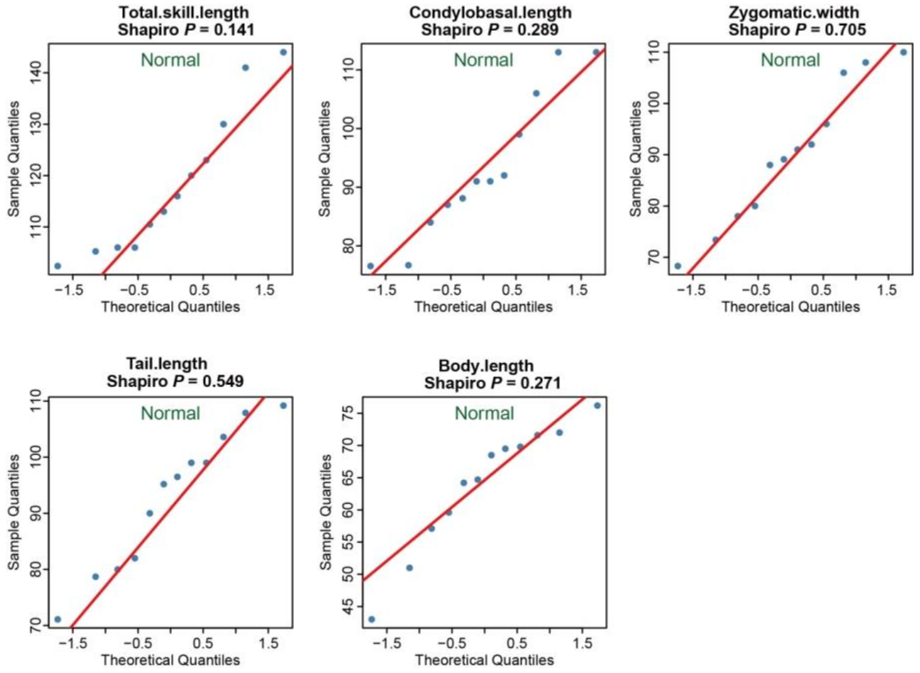

Fig.S1. Quantile-Quantile (Q-Q) plots for normality assessment of 5 morphological traits across 12 species. Each panel represents a trait, with points closely aligning to the reference line (red), indicating adherence to a normal distribution.

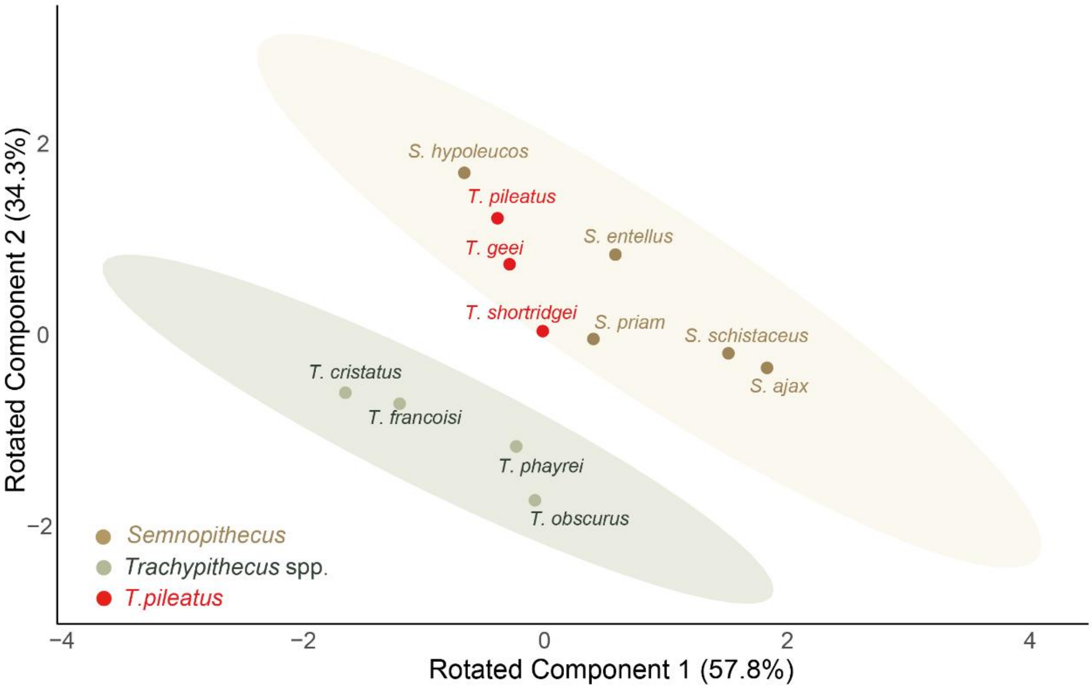

Fig.S2. Principal Component Analysis of Morphological Data with Varimax Rotation in *Semnopithecus* and *Trachypithecus* species. *Trachypithecus* spp. representing other *Trachypithecus* species.

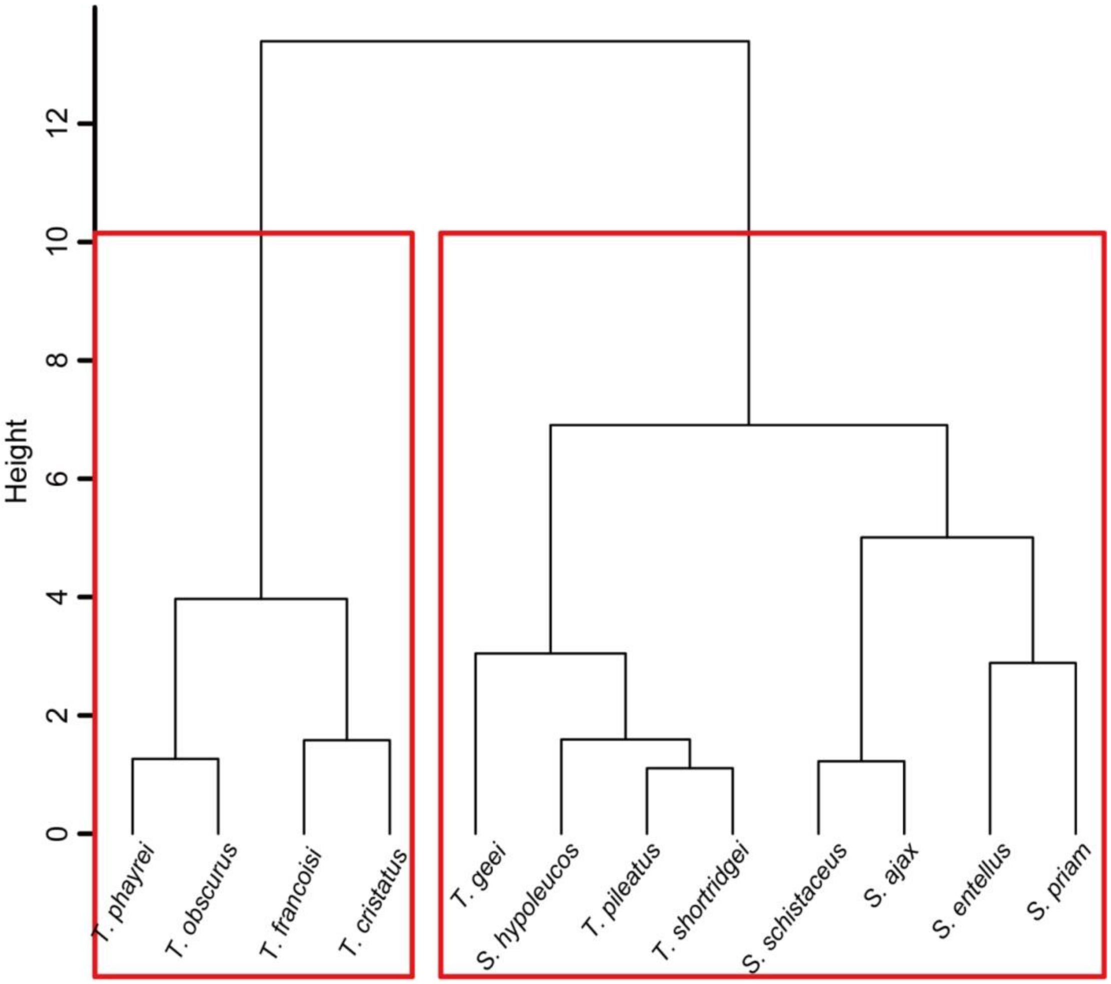

Fig.S3. Dendrogram from hierarchical clustering of 12 species based on 5 morphological traits using Manhattan distance and complete linkage. The tree was cut into 2 clusters (highlighted in red).

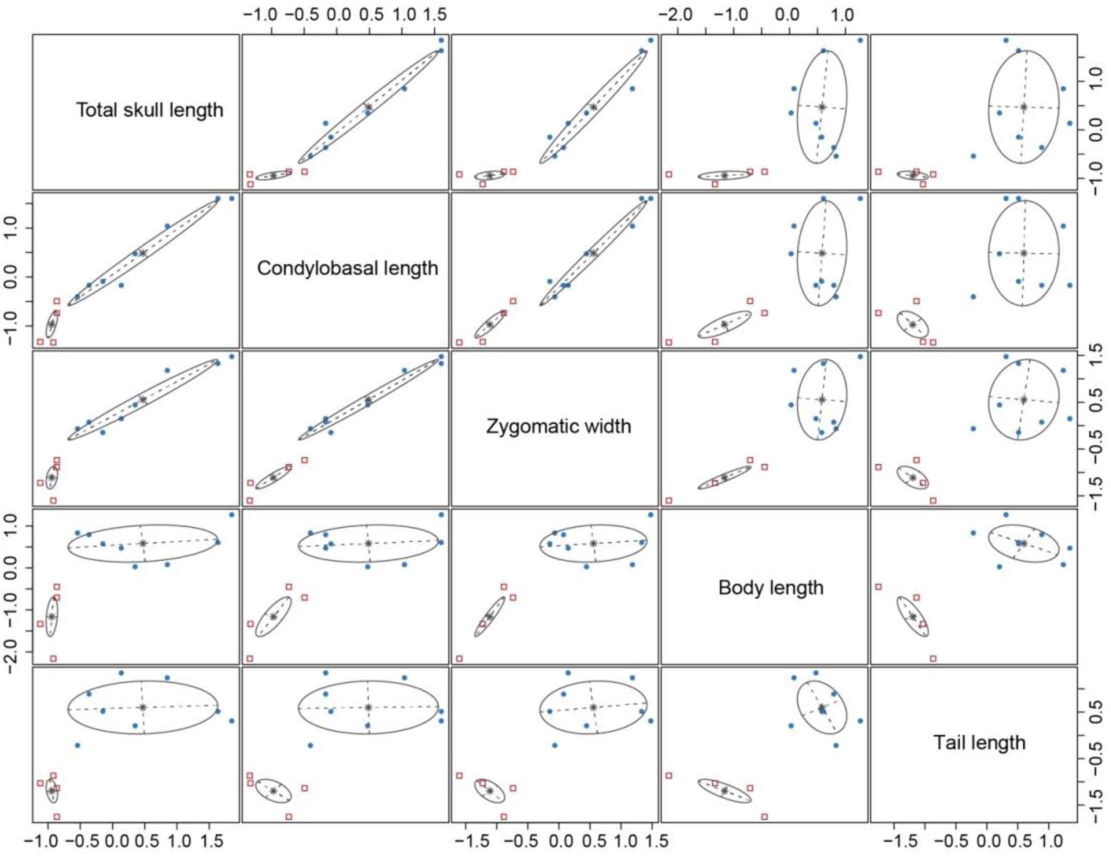

Fig.S4. Gaussian mixture model clustering results with forced 2-component solution. All features were standardized prior to analysis.

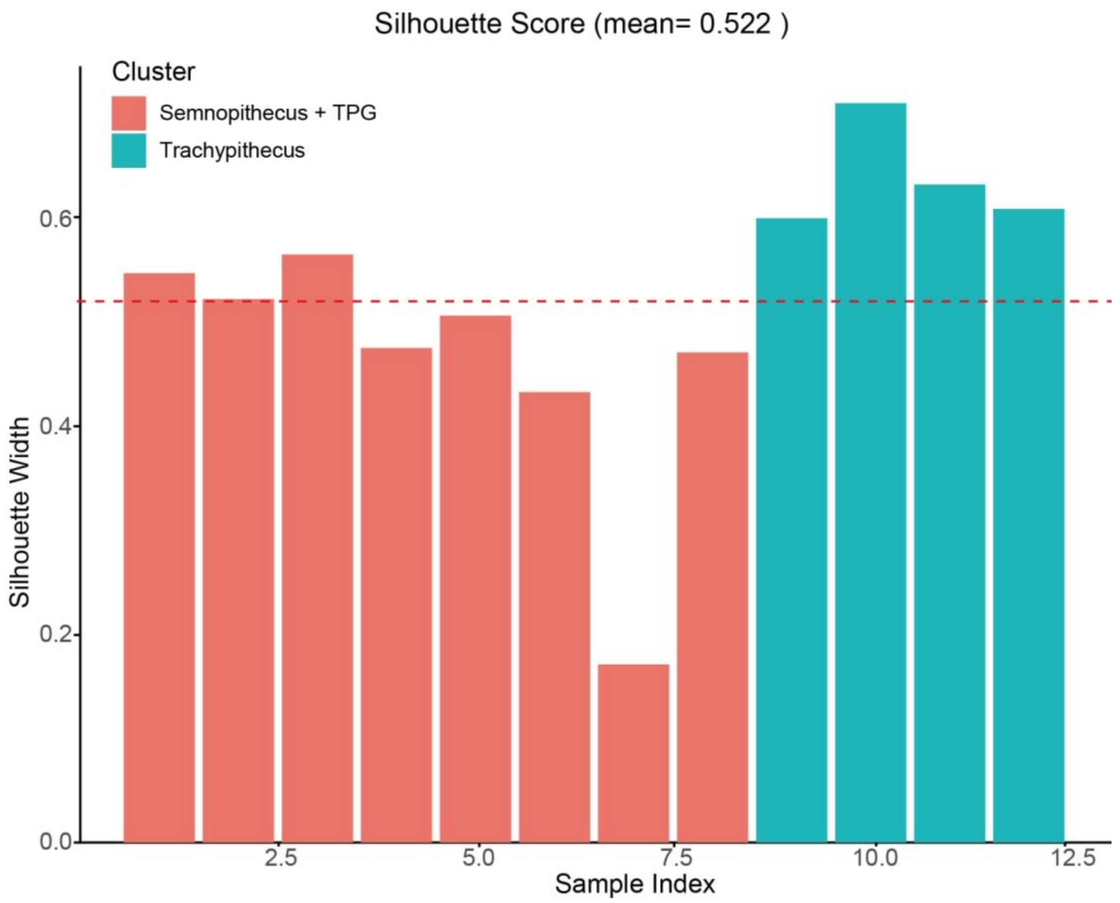

Fig.S5. Silhouette plot (GMM, k=2). Bars show per-sample cohesion/separation (mean=0.522). Cluster 2 shows better internal consistency.

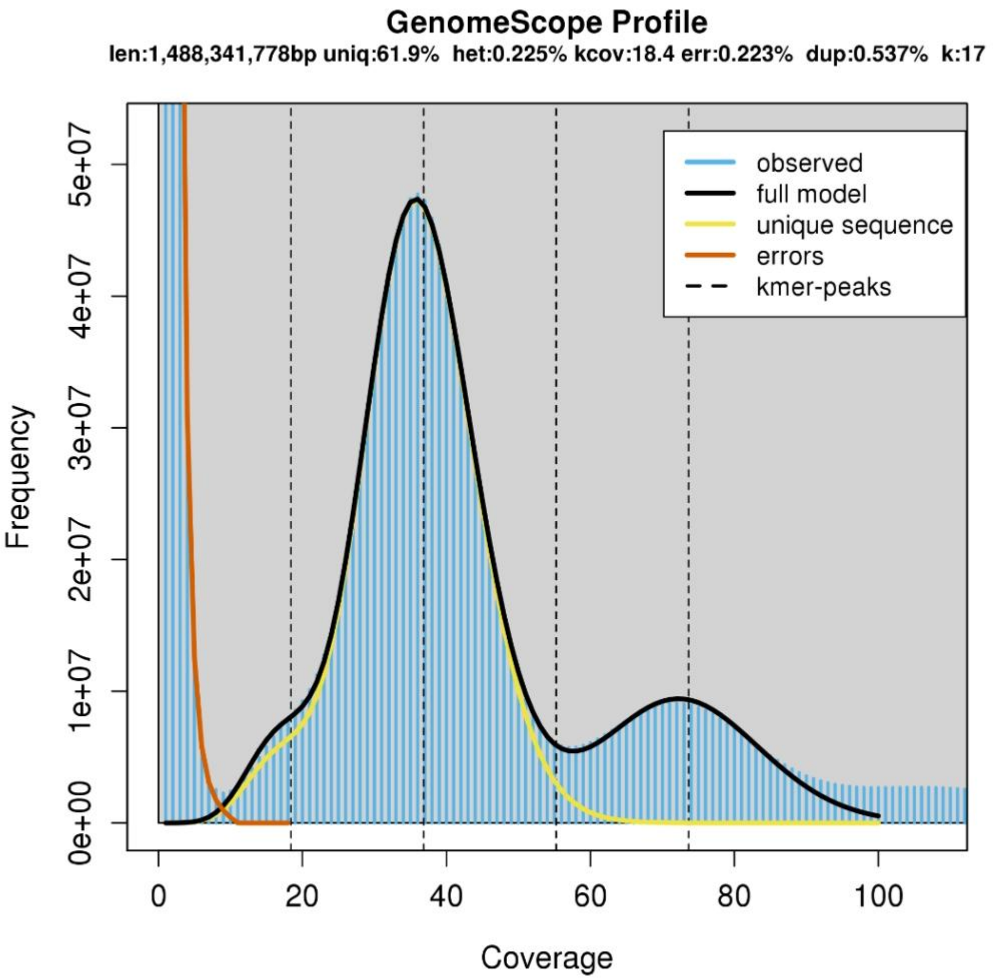

Fig.S6. K-mer frequency distribution of the genomic data for *T. shortridgei.*

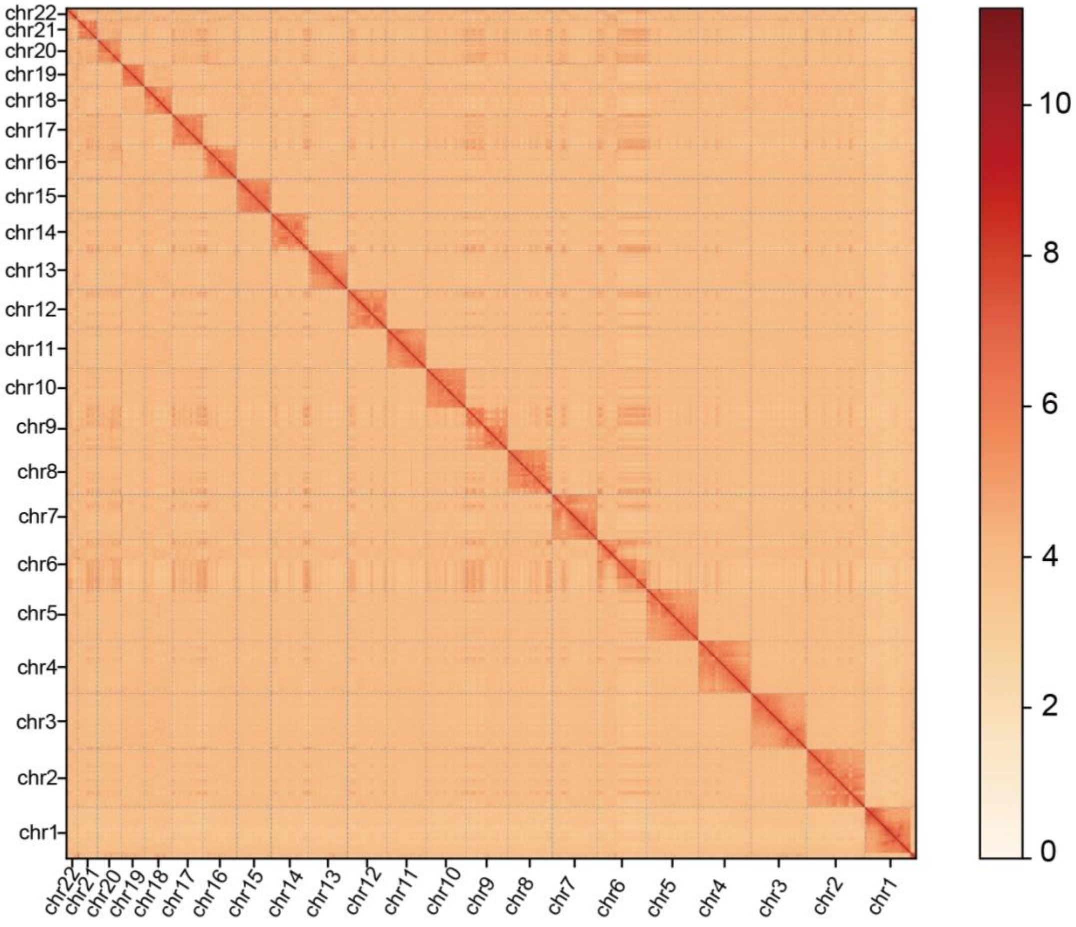

Fig.S7. Hi-C heatmap of *T. shortridgei* genome. The heatmap illustrates the interaction patterns among the 22 pairs of chromosomes in the *T. shortridgei* genome, based on the frequency of chromatin interactions within 100kb genomic regions. The darker the color, the higher the interaction frequency.

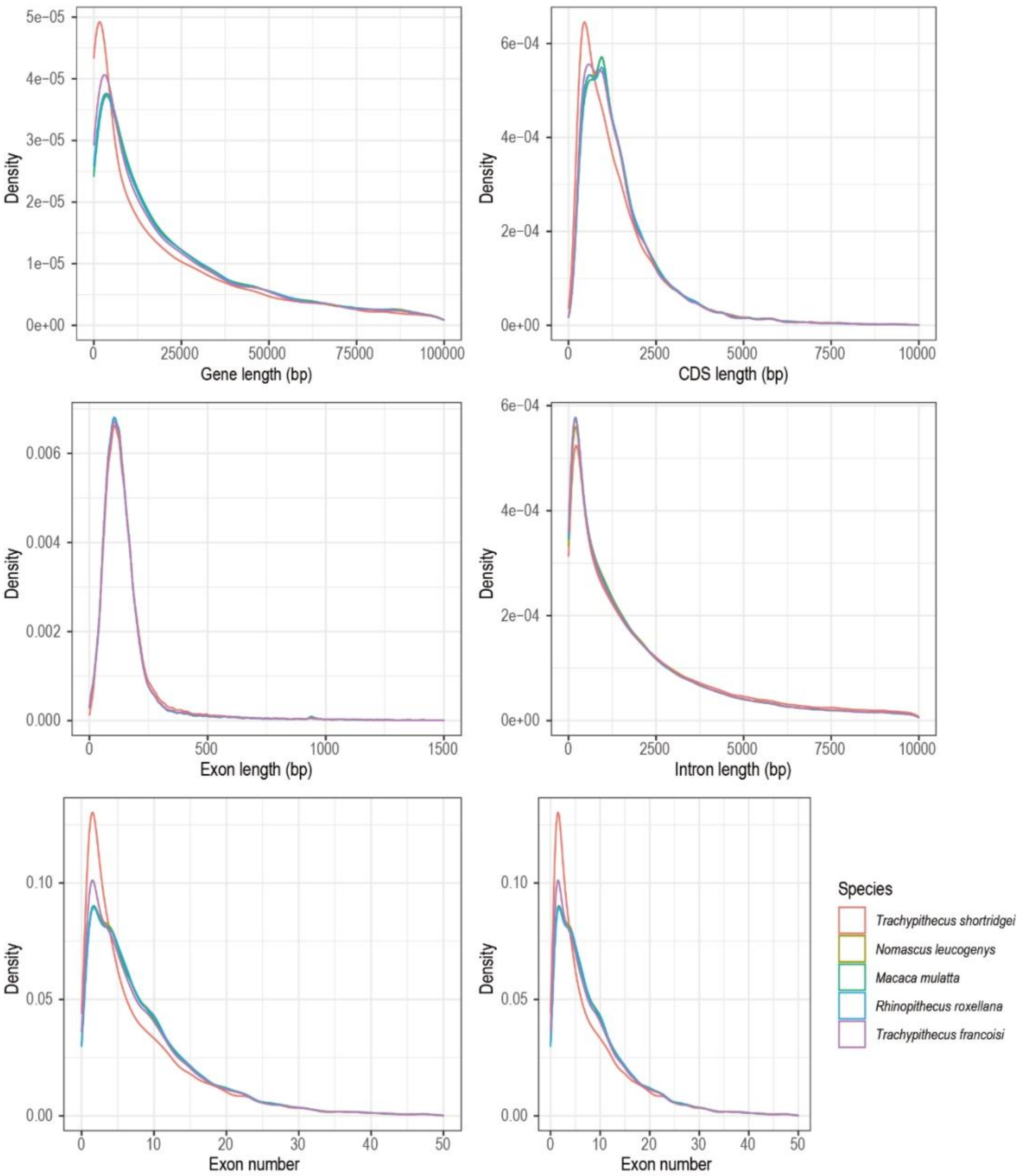

Fig.S8. Comparison of genomic elements from homologous species.

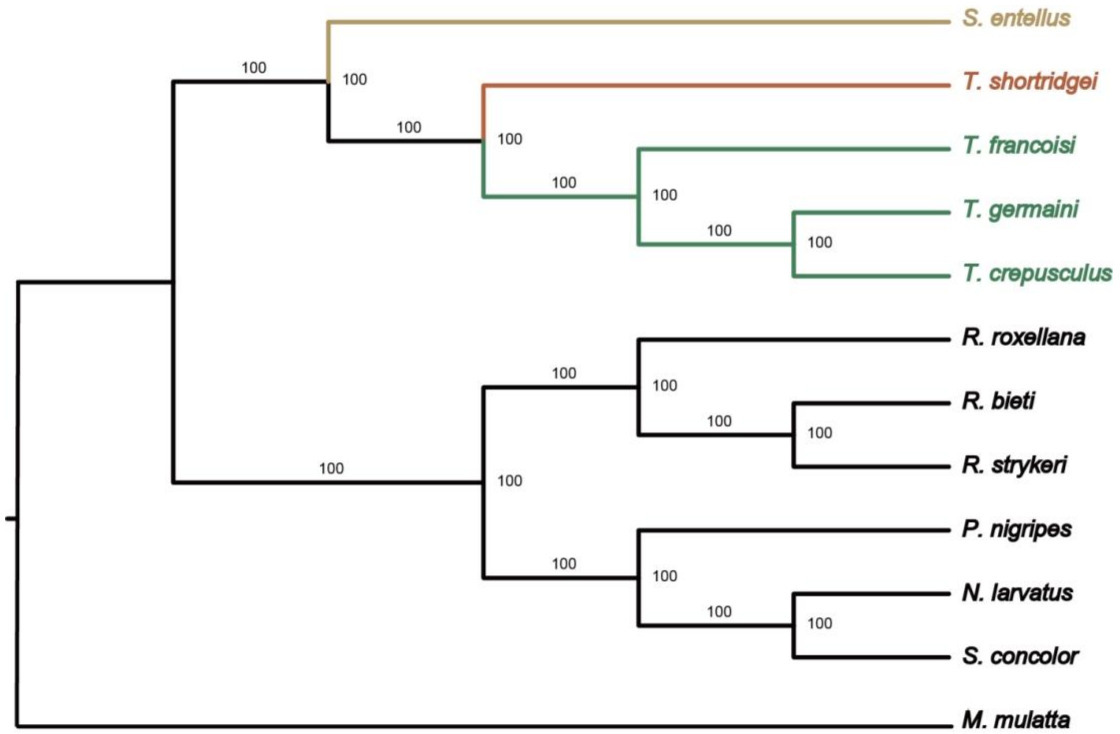

Fig.S9. ML phylogenetic tree of Asia colobines inferred from 11193 orthologous Genes.

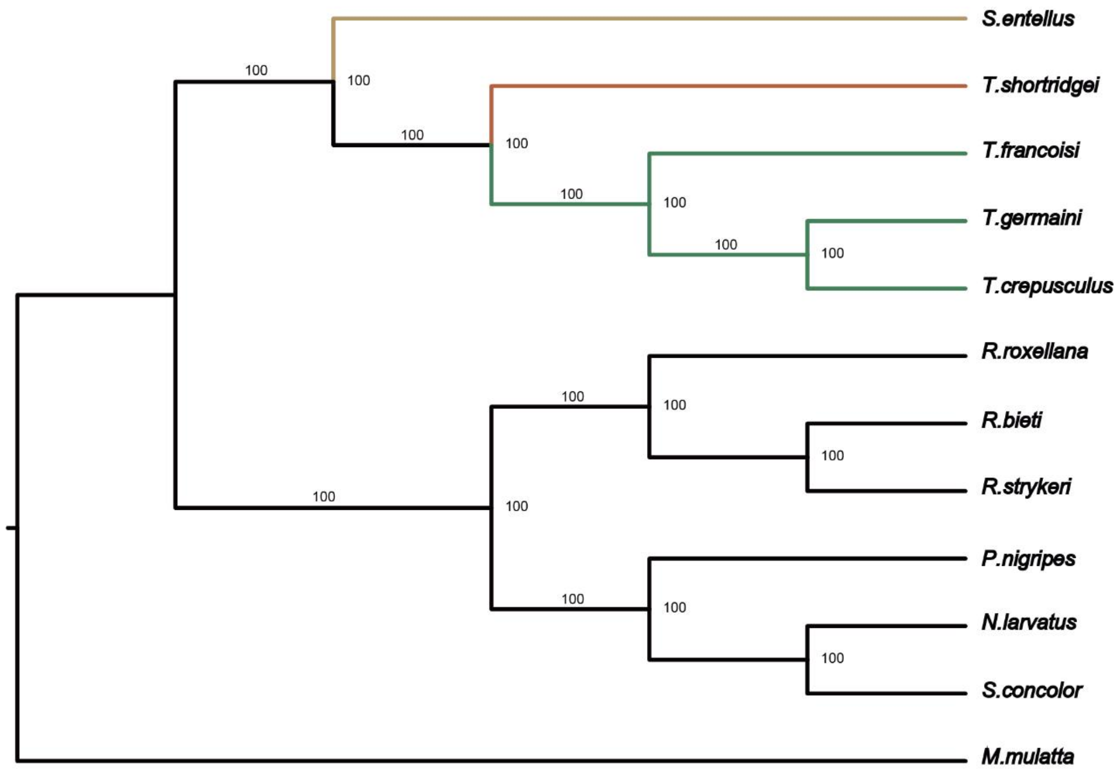

Fig.S10. ML phylogenetic tree of Asia colobines inferred from 4d sites.

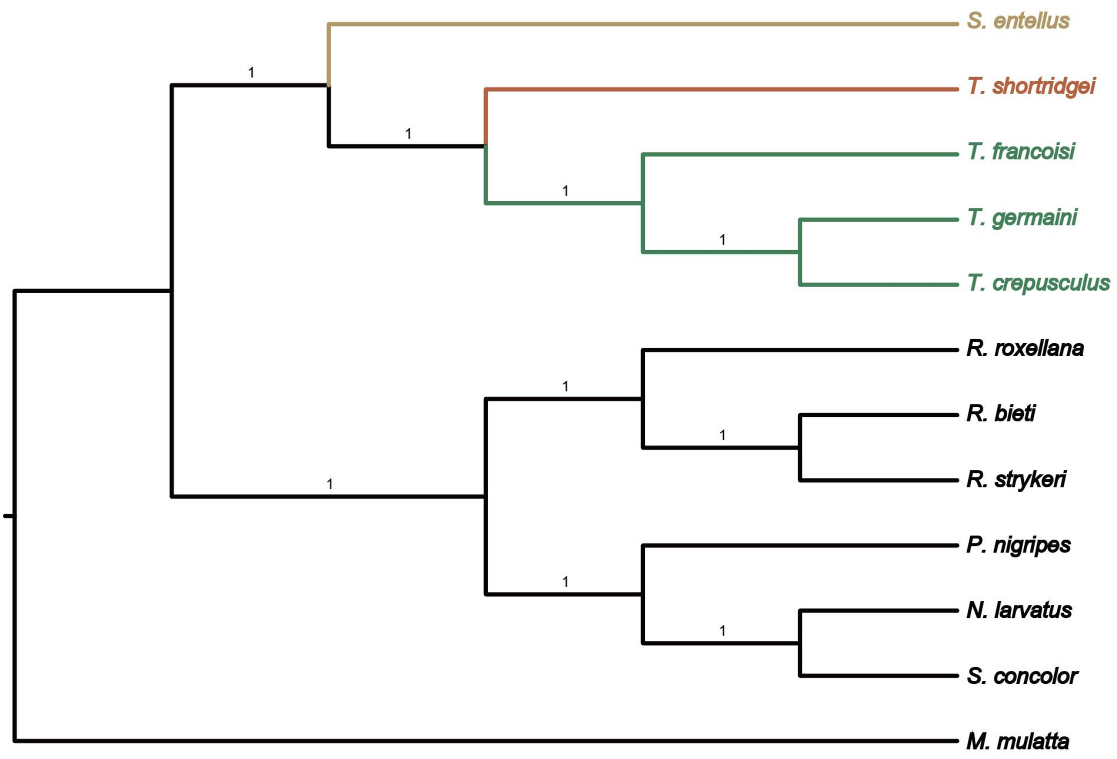

Fig.S11. ML phylogenetic tree of Asia colobines inferred from conserved non-exonic elements.

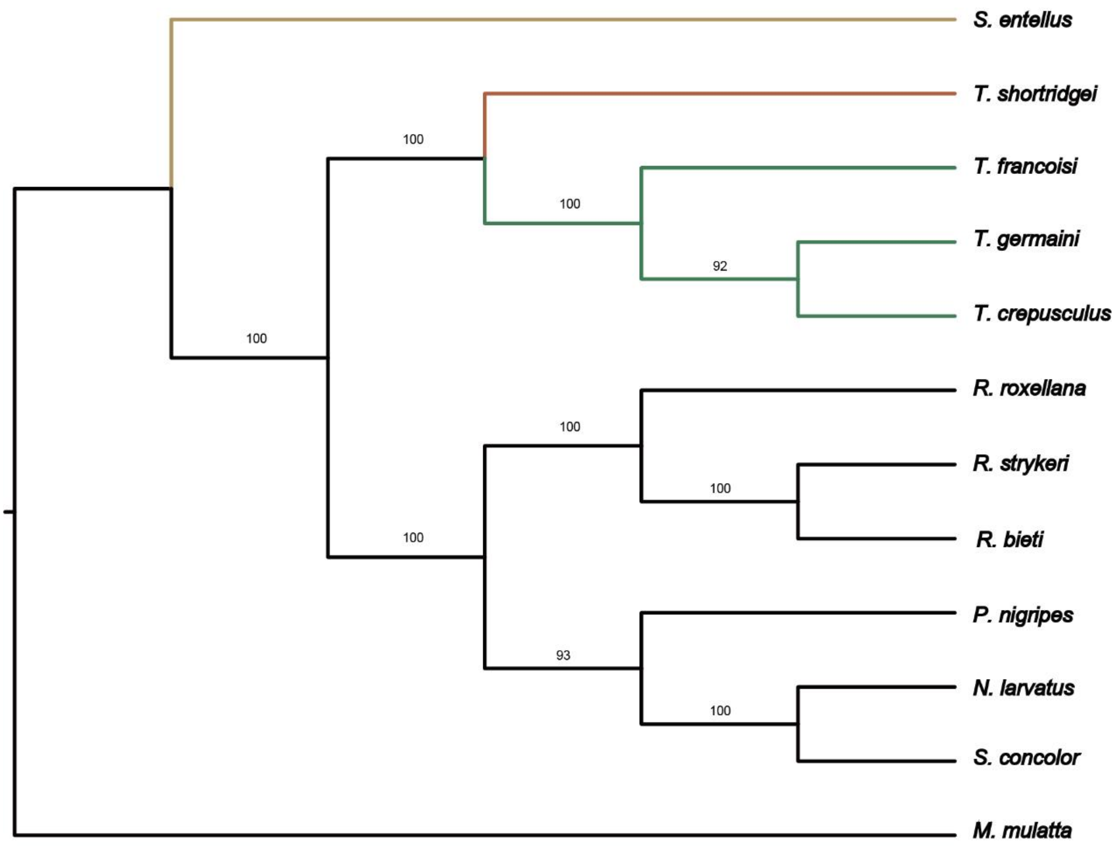

Fig.S12. ML phylogenetic tree of Asia colobines inferred from mitochondrial genomic DNA.

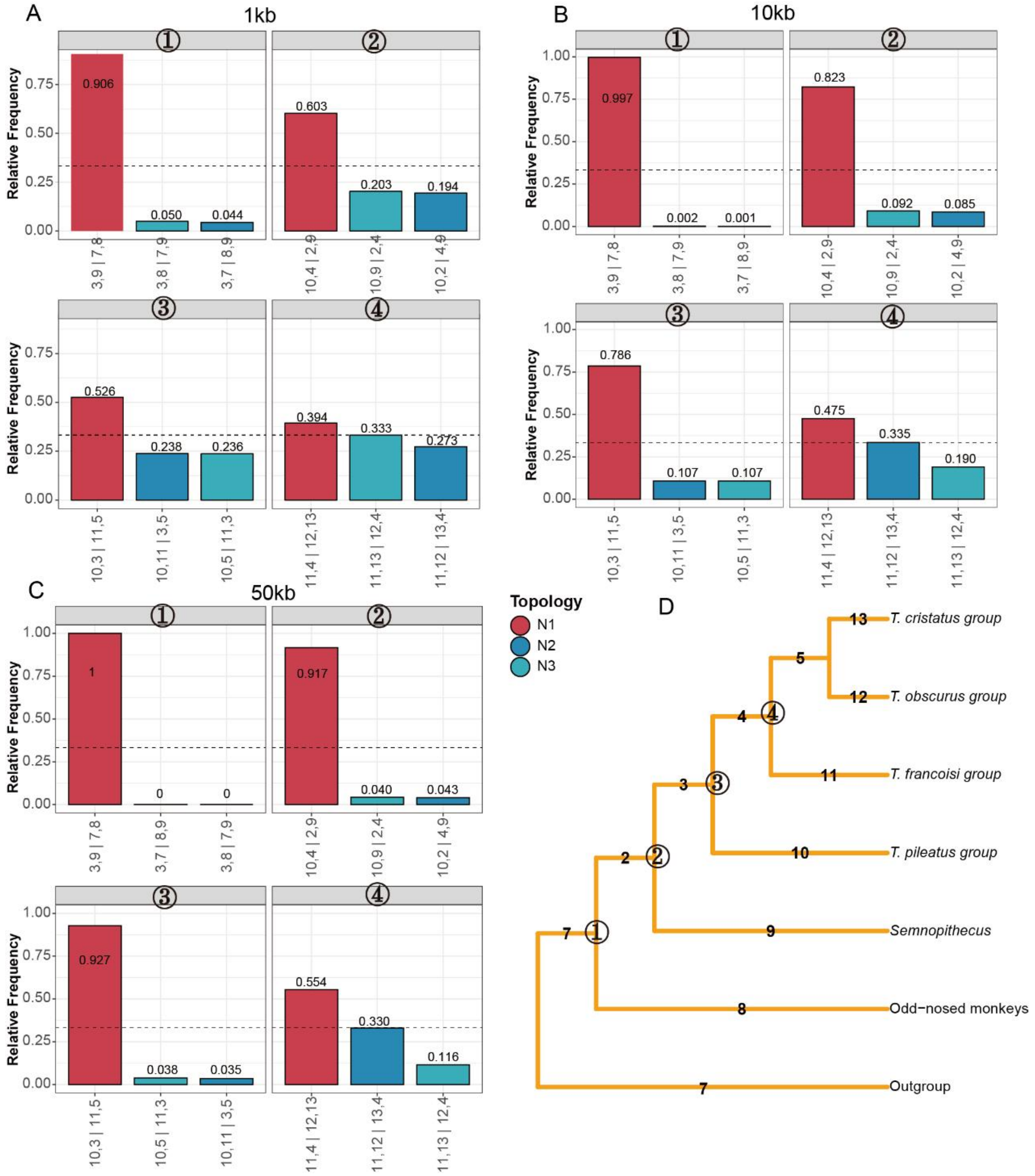

Fig.S13. The frequency of three topology presented on the branches for four groups of *Trachypithecus* and *Semnopithecus*. A: The frequency of three topology from 1 kb window size across all chromosomes; B: The frequency of three topology from 10 kb window size in genome; C: The frequency of three topology from 50 kb window size in genome. The red bar represents the frequency of the main topology (based on the ASTRAL species tree); the other two topologies are shown with varying shades of blue. The exact frequency of each topology is marked at the top of the bar. The dotted line indicates the threshold of 1/3 expected at random. The number at the top of each graph corresponds to the label of the corresponding internal branch in Figure D. The x-axis shows the type of topology, corresponding to the branch numbers in Figure D, with adjacent branches separated by " | " (left); Each internal branch has four neighboring branches (right).

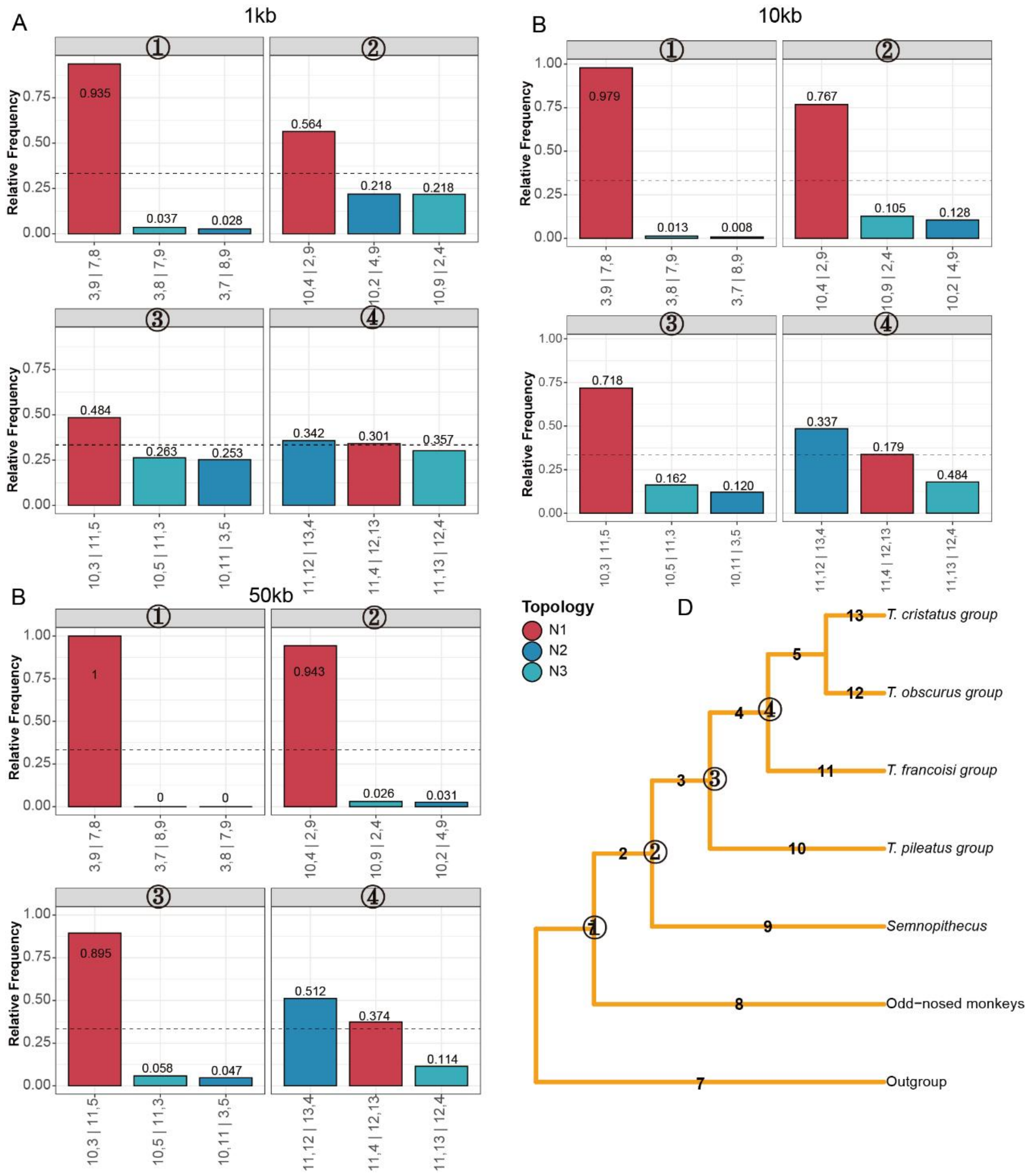

Fig.S14. The frequency of three topology presented on the branches for four groups of *Trachypithecus* and *Semnopithecus*. A: The frequency of three topology from 1 kb window size in X chromosome; B: The frequency of three topology from 10 kb window size in genome; C: The frequency of three topology from 50 kb window size in genome. The red bar represents the frequency of the main topology (based on the ASTRAL species tree); the other two topologies are shown with varying shades of blue. The exact frequency of each topology is marked at the top of the bar. The dotted line indicates the threshold of 1/3 expected at random. The number at the top of each graph corresponds to the label of the corresponding internal branch in Figure D. The x-axis shows the type of topology, corresponding to the branch numbers in Figure D, with adjacent branches separated by " | " (left); Each internal branch has four neighboring branches (right).

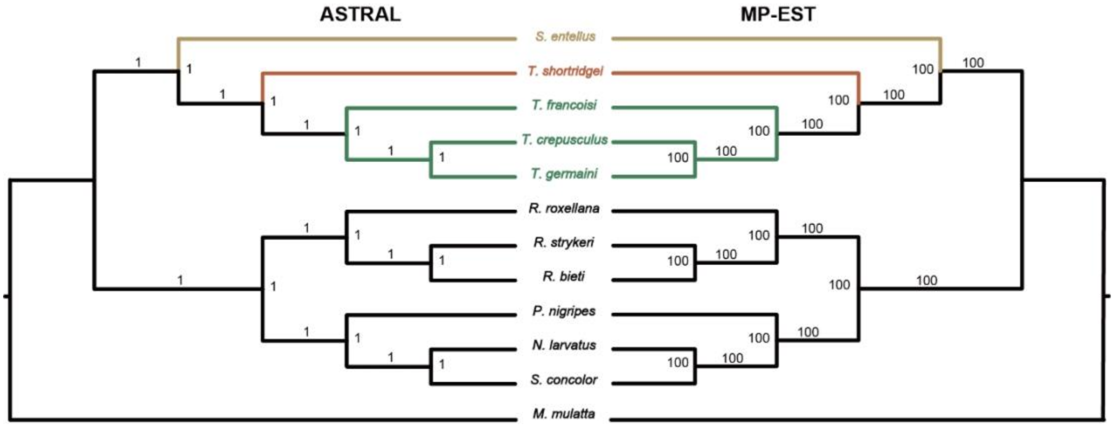

Fig.S15. The species tree inferred by ASTRAL and MP-EST based on all chromosomes.

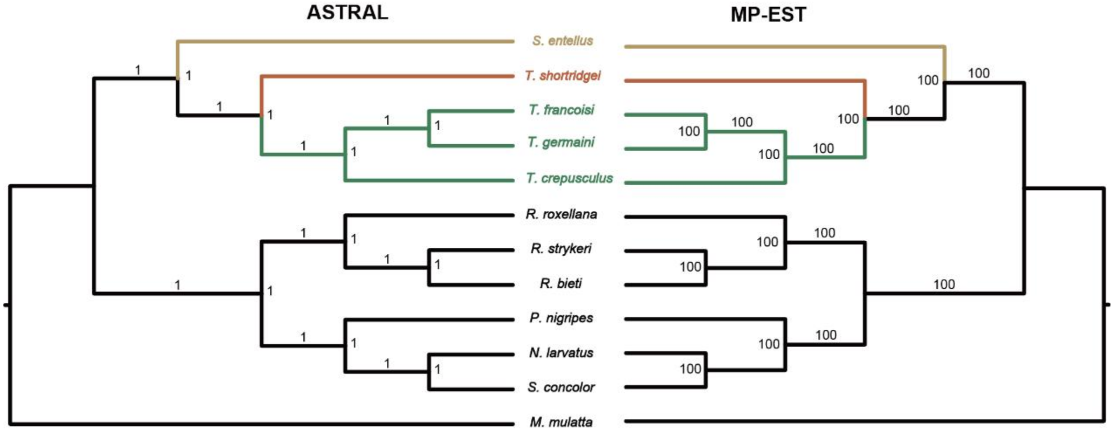

Fig.S16. The species tree inferred by ASTRAL and MP-EST based on X chromosome.

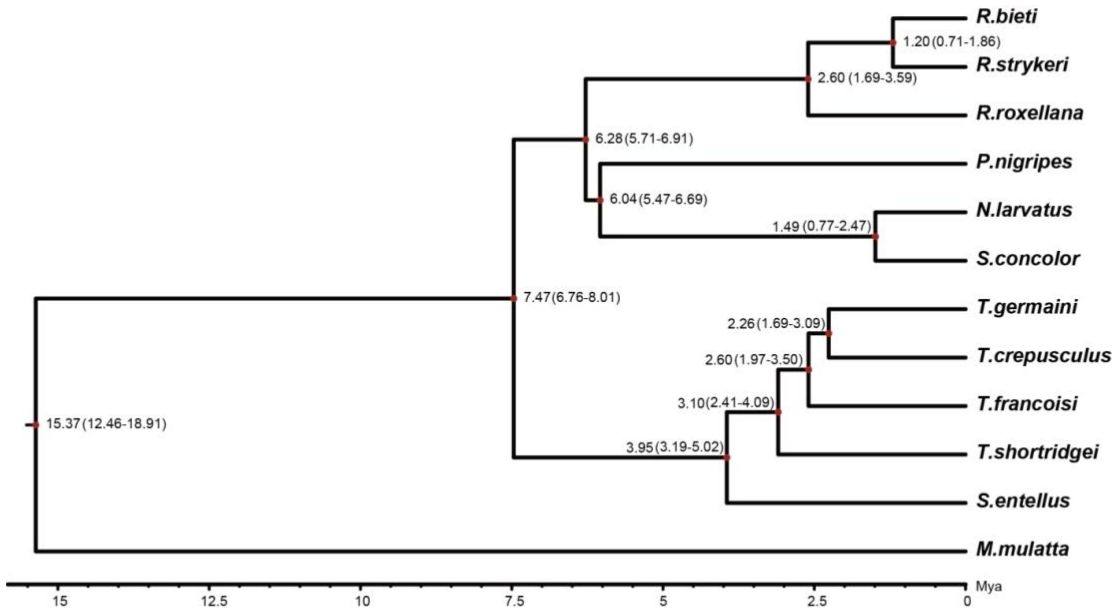

Fig.S17. Estimation of divergence times. The divergence time is indicated by the black number near each node. Five soft bound calibration time points have been applied. It should be noted that the divergence time estimated here could be affected by the selection of calibration time.

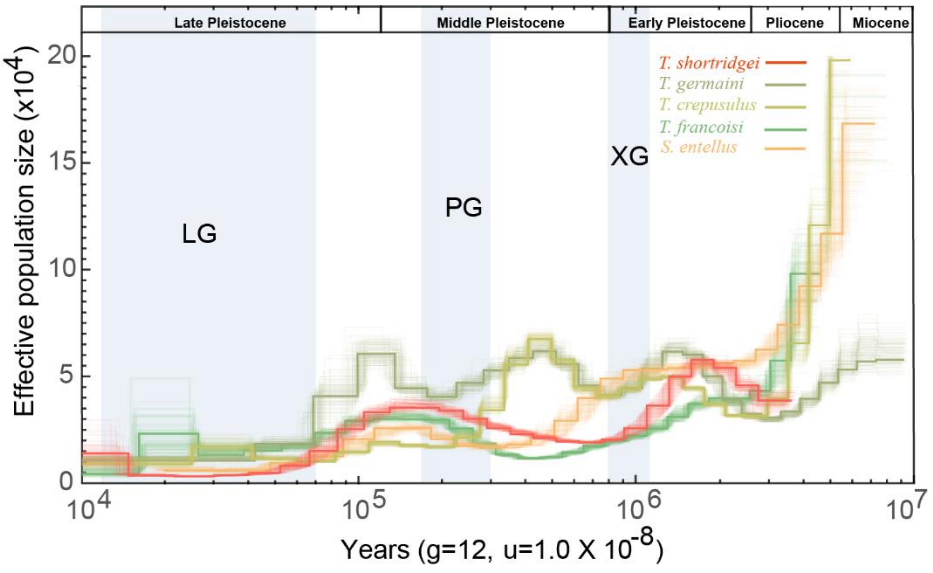

Fig.S18. PSMC analyses across five representative species of *Semnopithecus* and *Trachypithecus*. The population histories for the various species are distinguished by different colors. LG: Last Glaciation; PG: Penultimate Glaciation; XG: Xixibangma Glaciation.

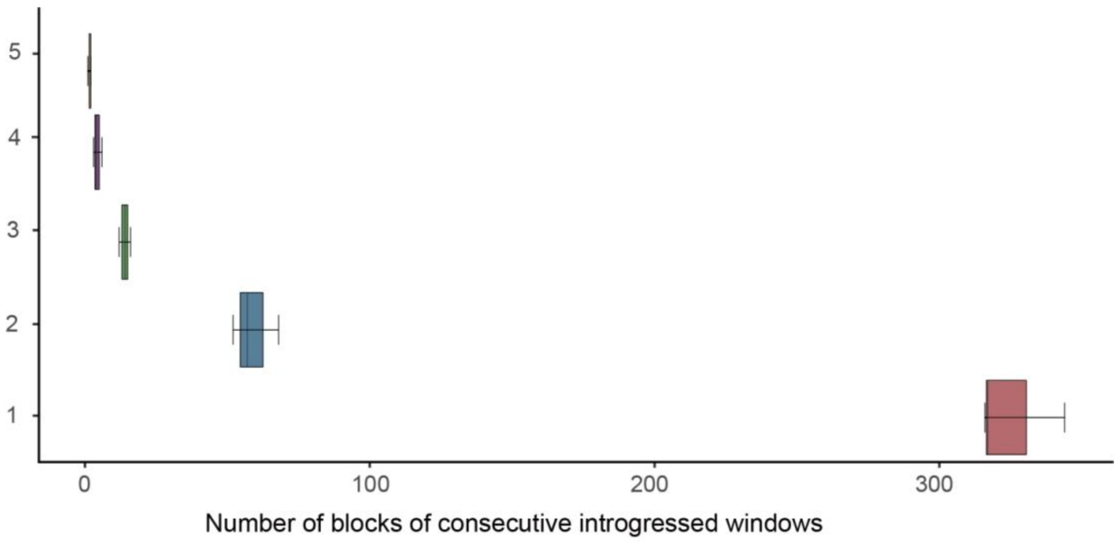

Fig.S19. Number of blocks of consecutive windows for introgression between *T. shortridgei* and *S. entellus* based on 10 kb windows. In all boxplots, the central line is the median, and lower and upper hinges represent the first and third quartiles.

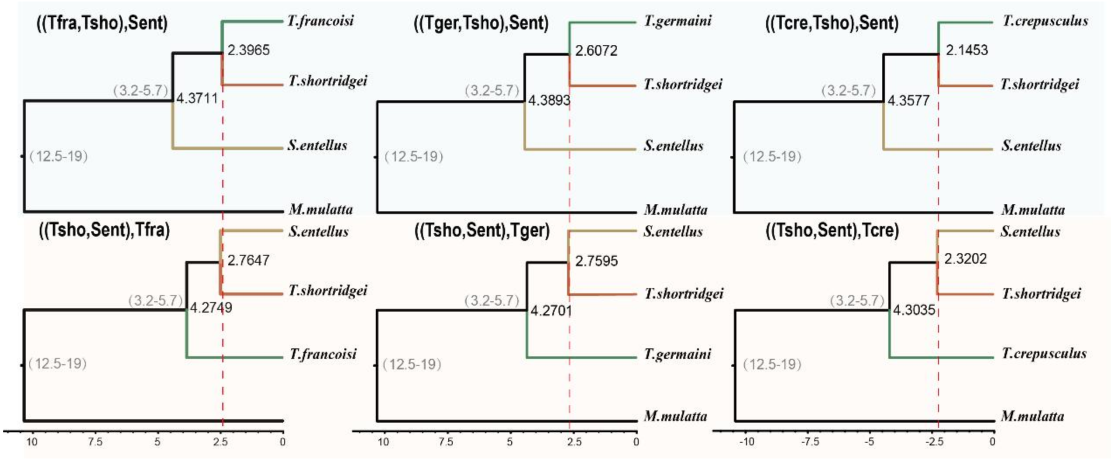

Fig.S20. Using the estimated divergence times to distinguish between ILS and hybridization scenarios. The phylogenetic trees illustrate the estimated divergence times among six species.

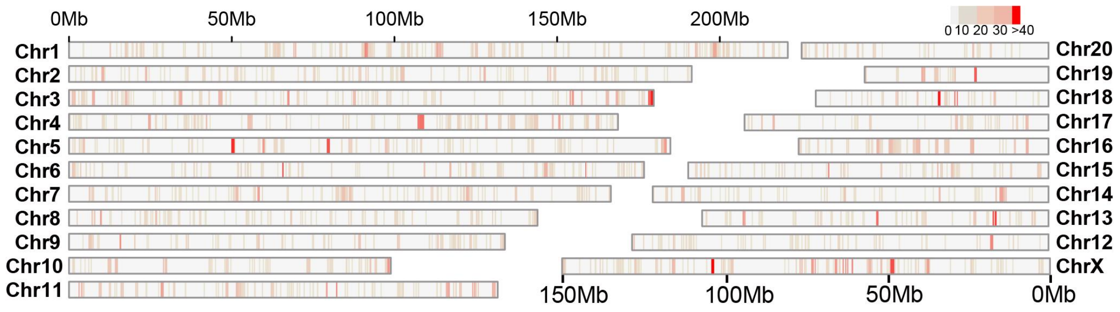

Fig.S21. The distributions of ILS segments between *T. francoisi* and *S. entellus* in Combination 1 using the *Macaca mulatta* (rhesus macaque) genome coordinates as a reference. Redder colors indicate a higher aggregated level of ILS segments in the region.

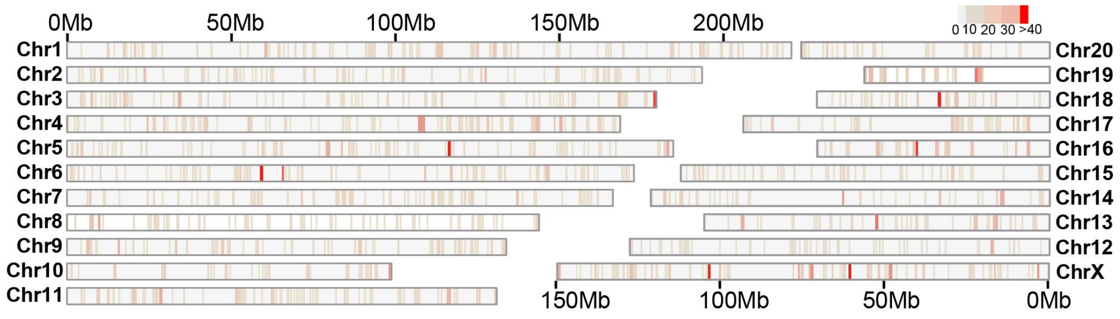

Fig.S22. The distributions of ILS segments between *T. shortridgei* and *S. entellus* in Combination 2 using the *Macaca mulatta* (rhesus macaque) genome coordinates as a reference. Redder colors indicate a higher aggregated level of ILS segments in the region.

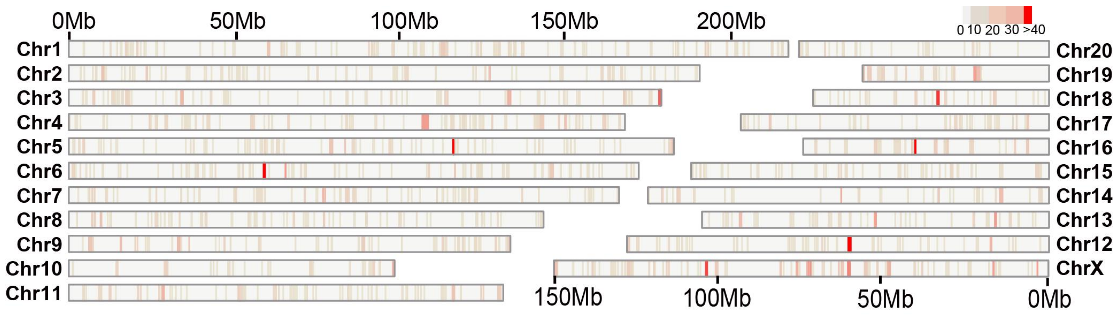

Fig.S23. The distributions of ILS segments between *T. germaini* and *S. entellus* in Combination 2 using the *Macaca mulatta* (rhesus macaque) genome coordinates as a reference. Redder colors indicate a higher aggregated level of ILS segments in the region.

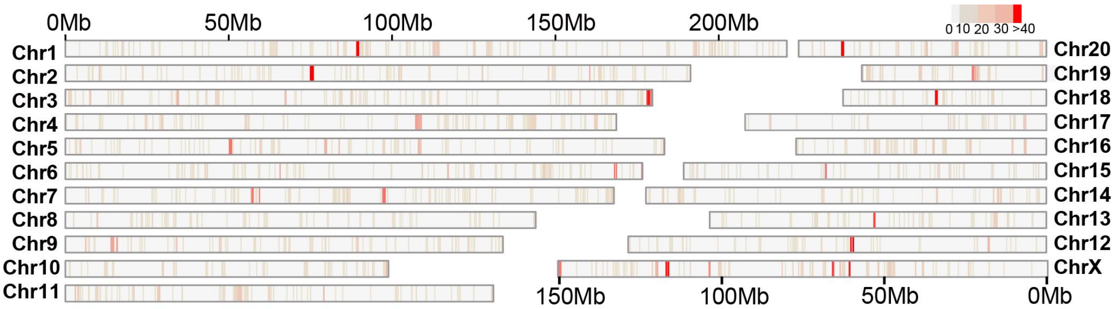

Fig.S24. The distributions of ILS segments between *T. crepusculus* and *S. entellus* in Combination 3 using the *Macaca mulatta* (rhesus macaque) genome coordinates as a reference. Redder colors indicate a higher aggregated level of ILS segments in the region.

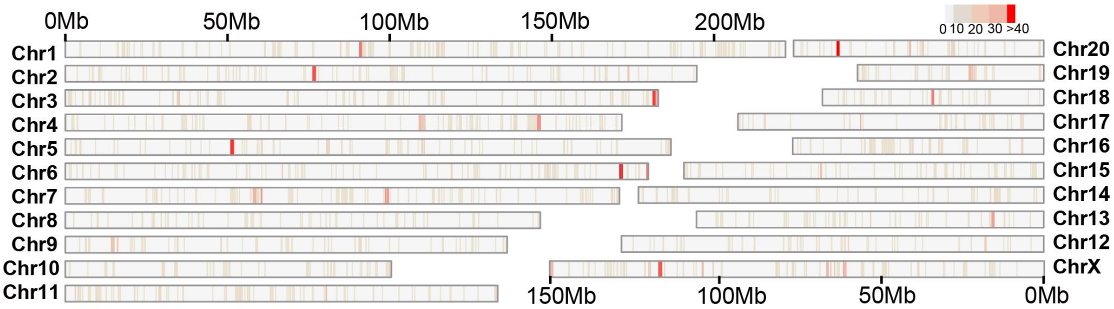

Fig.S25. The distributions of ILS segments between *T. shortridgei* and *S. entellus* in Combination 3 using the *Macaca mulatta* (rhesus macaque) genome coordinates as a reference. Redder colors indicate a higher aggregated level of ILS segments in the region.

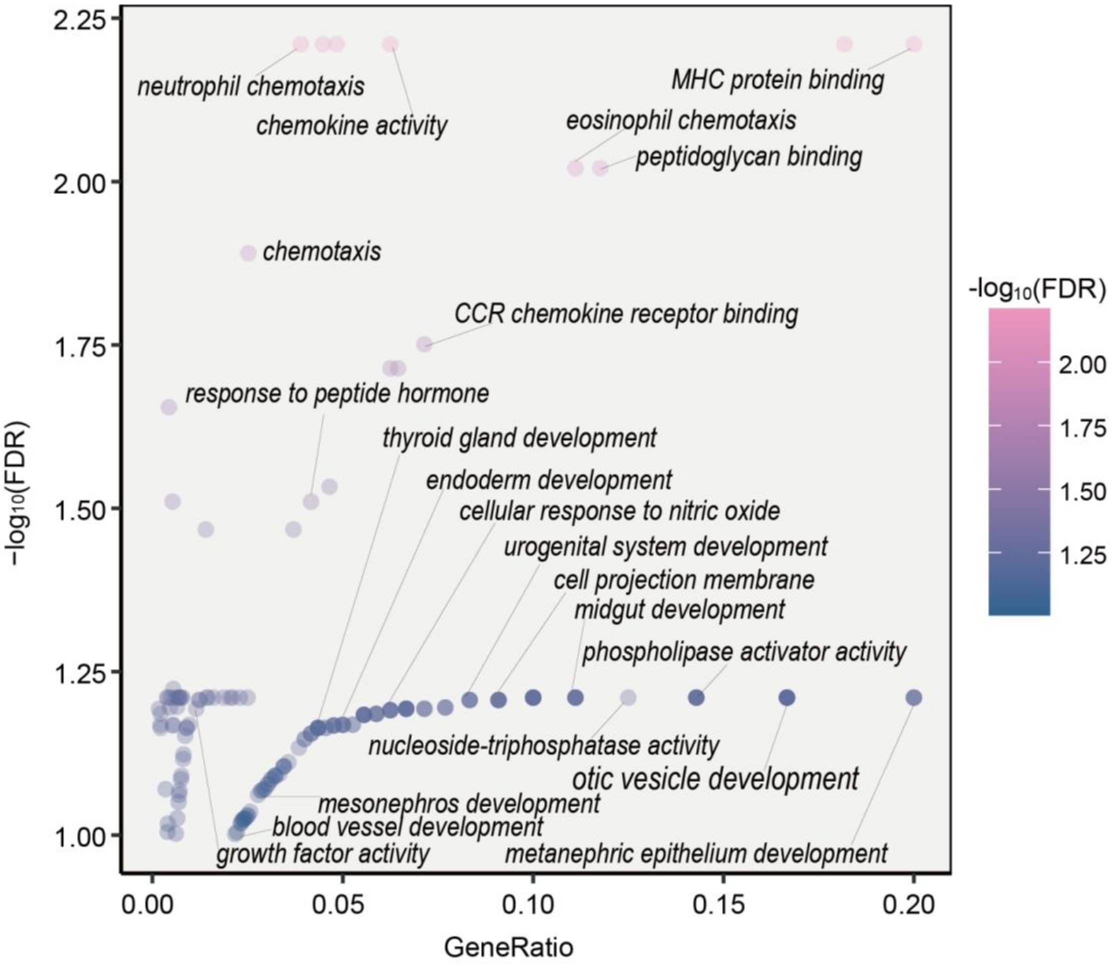

Fig.S26. GO enrichment analysis of 77 ILS genes in *T.shortridgei* and *S.entellus*. GeneRatio represents the ratio of the number of genes enriched in a given GO entry to the total number of genes; The FDR value represents the value corrected for the *P*-value; The size of the dot represents the number of genes; The color of the dots represents $-\log_{10}$(FDR), and the higher the value of $-\log_{10}$(FDR), the redder the color.

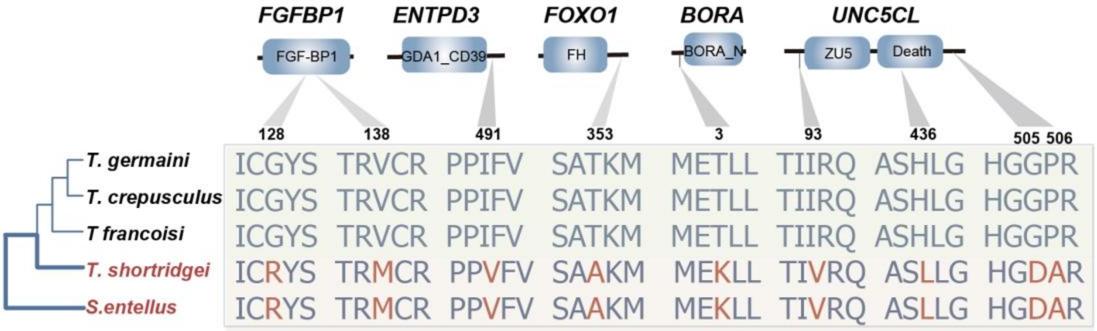

Fig.S27. Five genes related to bone development with ILS signal sites in *T. shortridgei* and *S. entellus*. The alignments of amino acid sequences are plotted for five *Semnopithecus* and *Trachypithecus* species, showing the variation at the target *T. shortridgei* and *S. entellus* ILS signal sites marked in red.

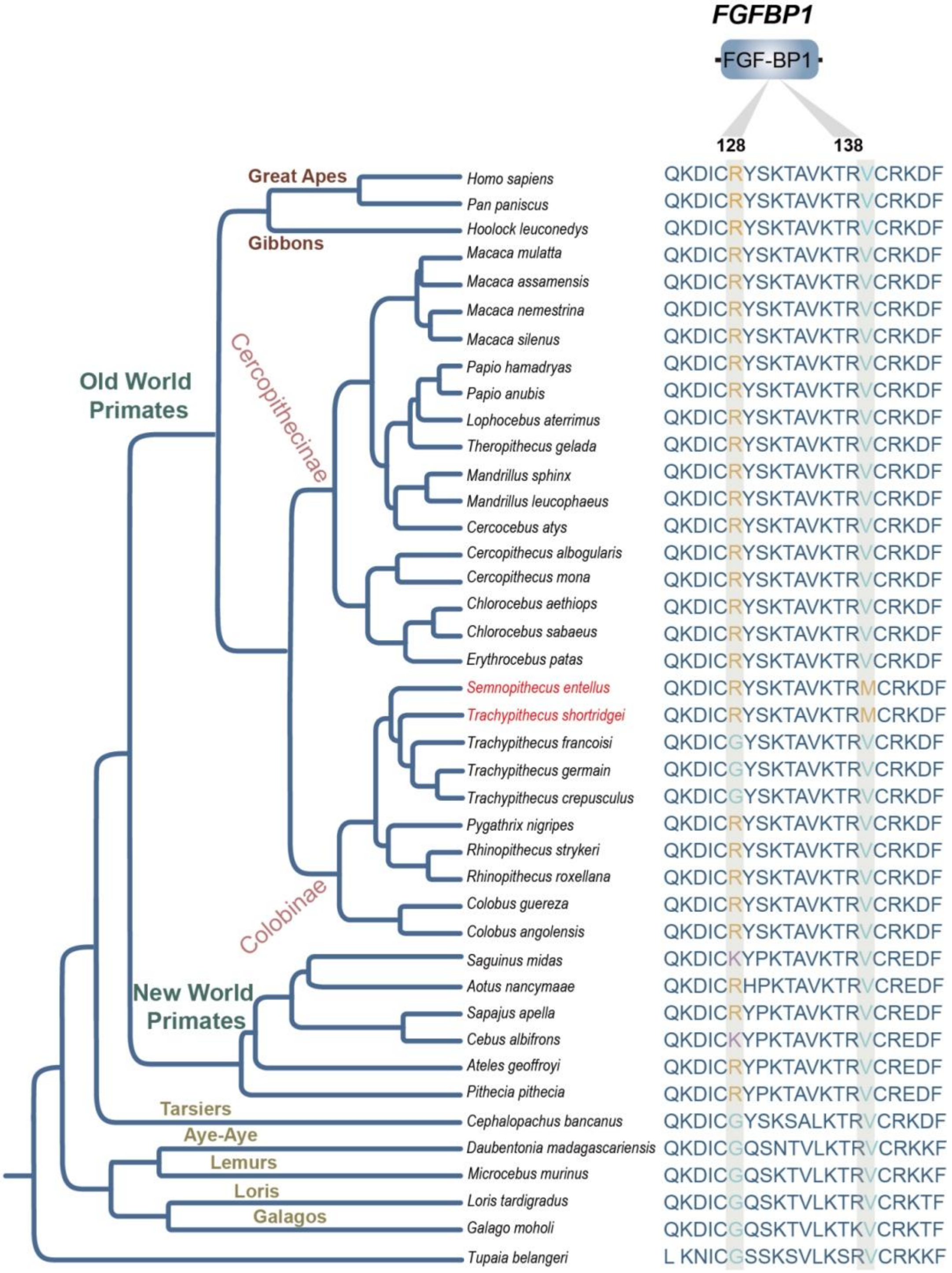

Fig.S28. Status of the ILS site of *FGFBP1* gene in primate. This alignment included *T. shortridgei* in this study and forty primates and used *Tupaia belangeri* as an outgroup. All sequences were obtained from Primates Genome Project (9), except *T. shortridgei.* In this alignment, G and R are two ancestral alleles at the 128 site; M in the TPG and *Semnopithecus* species is a specific mutation at the 138 site.

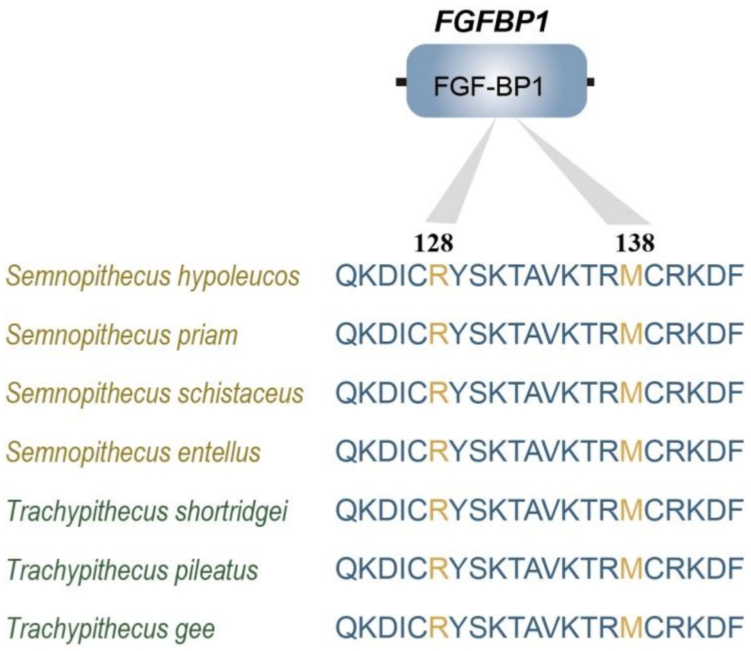

Fig.S29. Status of the ILS site of *FGFBP1* gene in the TPG and *Semnopithecus* species.

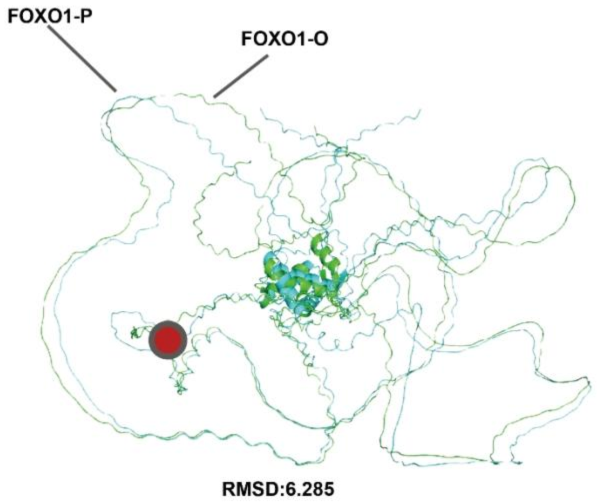

Fig.S30. The predicted 3D structure of FOXO1. Blue represents the same mutation type at amino acid position 353 of the TPG and *Semnopithecus* species, and green represents the mutation type at amino acid position 353 of the other *Trachypithecus* species. The red circles represent the missense mutations site of the TPG and *Semnopithecus* species.

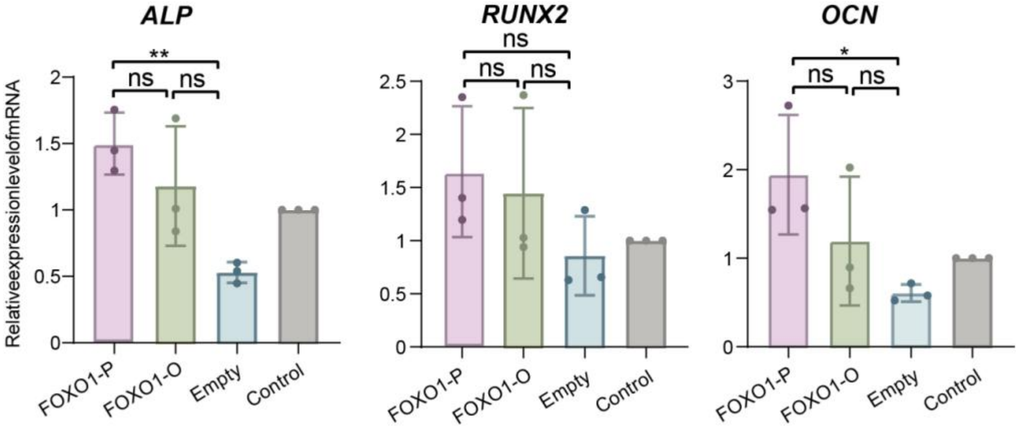

Fig.S31. The statistical comparison of relative expression levels of mRNA. The *ALP*, *RUNX2*, *OCN* osteogenic genes expression no significantly higher in FOXO1-P compared to FOXO1-O. $**P < 0.01$, $*P < 0.05$, ns: not significant. The empty plasmid was used for normalization.

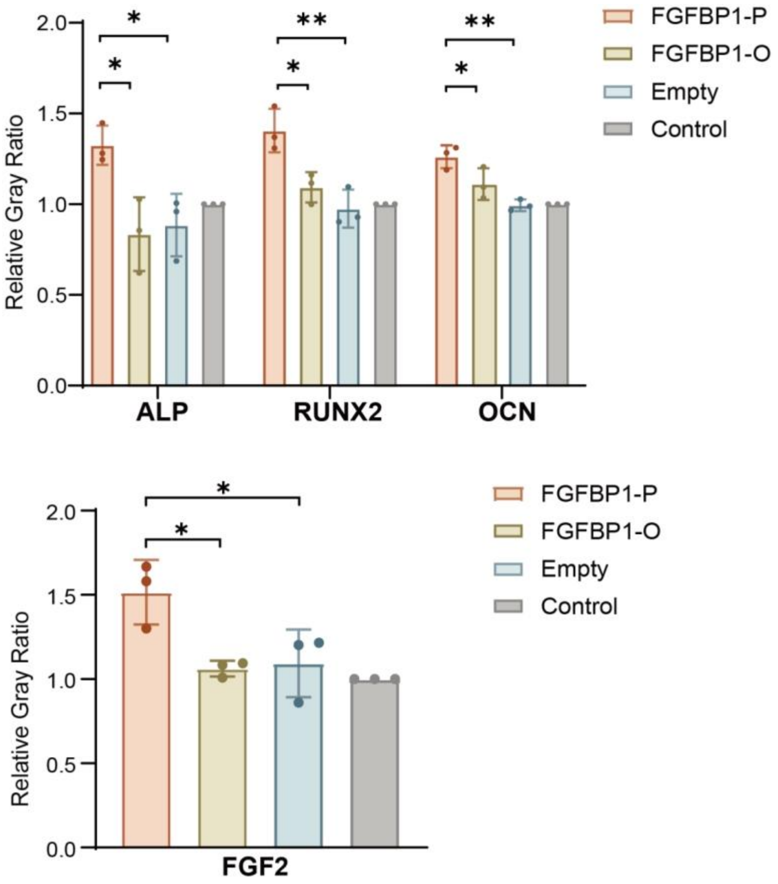

Fig.S32. The statistical comparison of relative expression levels of protein. The ALP, RUNX2, OCN protein expression significantly higher in FGFBP1-P compared to FGFBP1-O. $**P < 0.05$, $*P < 0.01$. The empty plasmid was used for normalization.

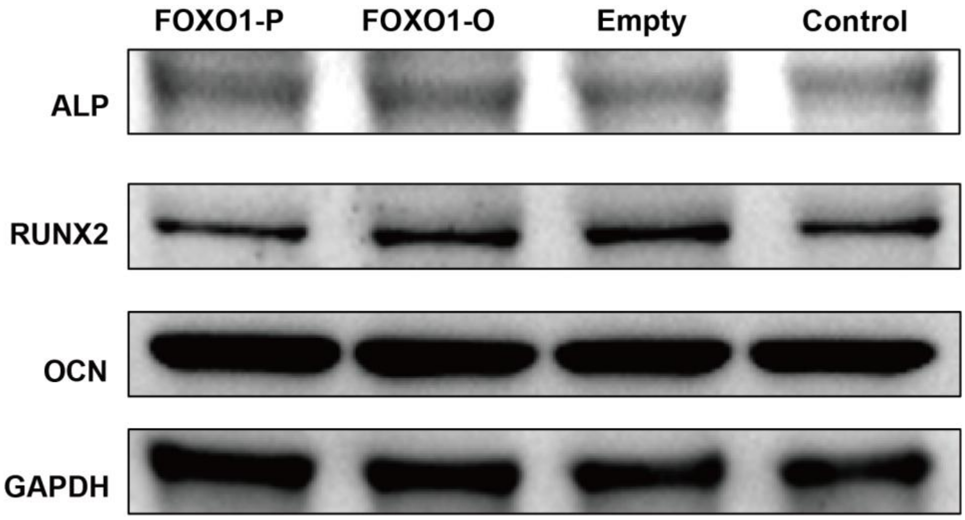

Fig.S33. The comparison of relative expression levels of protein. The protein expression levels of ALP, RUNX2, OCN between FOXO1-P and FOXO1-O in HJBMMSCs.

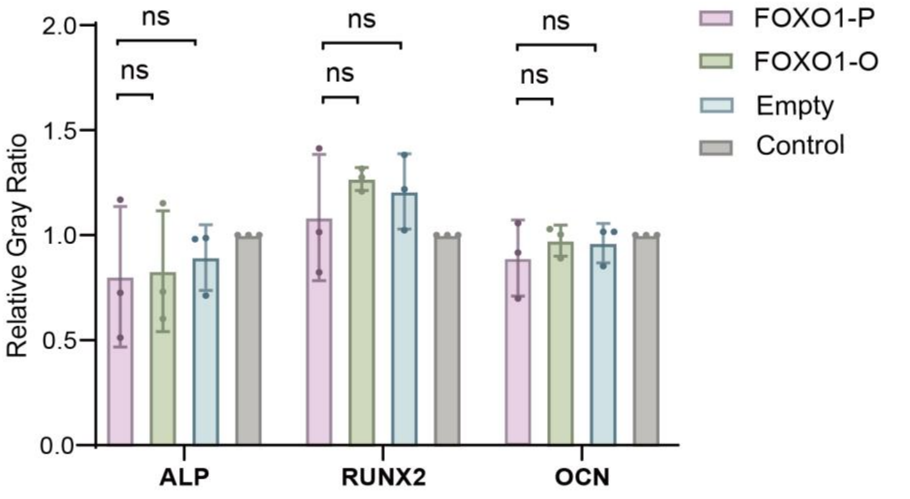

Fig.S34. The statistical comparison of relative expression levels of protein. The ALP, RUNX2, OCN protein expression no significantly higher in FOXO1-P compared to FOXO1-O. ns: not significant. The empty plasmid was used for normalization.

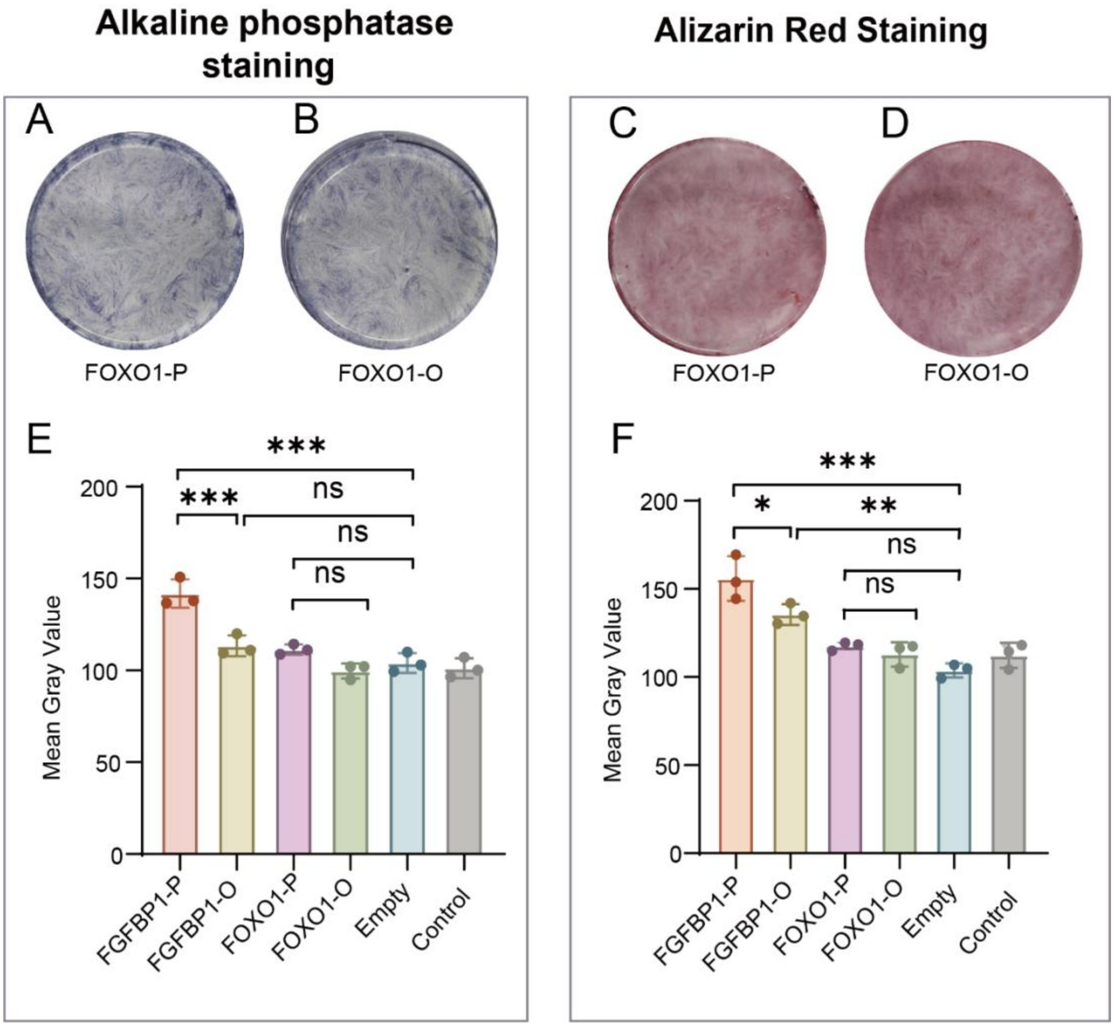

Fig.S35. The comparisons of osteogenic differentiation levels. (*A-D*) Osteogenic differentiation staining in HJBMMSCs transfected with FOXO1-P versus FOXO1-O. (*E-F*) The osteogenic differentiation capacity of FGFBP1-P significantly higher compared to FGFBP1-O, no significant difference was observed between FOXO1-P and FOXO1-O. ***$P < 0.001$, **$P < 0.01$, *$P < 0.05$, ns: not significant. The empty plasmid was used for normalization.

Table S1 The information of skull measurements and body size measurements from the TPG, *Semnopithecus* and *Trachypithecus*

Group	Species	Skull total length(mm)	Condylobasal length (mm)	Zygomatic width (mm)	Body length (cm)	Tail length (cm)
Semnopithecus	*Semnopithecus schistaceus*	141	113	108	69.8	99
Semnopithecus	*Semnopithecus ajax*	144	113	110	76.2	96.5
Semnopithecus	*Semnopithecus entellus*	130	106	106	64.7	107.9
Semnopithecus	*Semnopithecus priam*	123	99	96	64.2	95.2
Semnopithecus	*Semnopithecus hypoleucos*	120	91	92	68.5	109.2
T. pileatus group	*Trachypithecus pileatus*	116	92	88	69.5	99
T. pileatus group	*Trachypithecus geei*	110.5	88.1	89.1	72	90
T. pileatus group	*Trachypithecus shortridgei*	113	91	91	71.6	103.6
Trachypithecus	*Trachypithecus phayrei*	106	87	80	57.1	78.7
Trachypithecus	*Trachypithecus francoisi*	102.4	76.7	73.4	51	80
Trachypithecus	*Trachypithecus cristatus*	105.25	76.56	68.29	43	82
Trachypithecus	*Trachypithecus obscurus*	106	84	78	59.6	71.1

Table S2 Eigenvalues and percentage of variance explained by principal Components

	Elgenvalue	Percentage of variance	Cumulative percentage of variance
PC1	4.086	81.725	81.73
PC2	0.523	10.454	92.18
PC3	0.350	6.998	99.18
PC4	0.033	0.665	99.84
PC5	0.008	0.158	100

Table S3 Variable loadings on principal components

	PC1	PC2	PC3	PC4	PC5
Total skull length	0.937	-0.294	0.130	0.134	-0.016
Condylobasal length	0.956	-0.279	0.022	-0.064	0.063
Zygomatic width	0.988	-0.099	0.006	-0.099	-0.060
Body length	0.837	0.274	0.473	0.037	0.006
Tail length	0.785	0.523	0.330	0.003	0.010

Table S4 Varimax-Rotated Variable loadings on principal components

	RC1	RC2	h2#	u2##	com
Condylobasal length	0.94	0.27	0.96	0.044	1.2
Zygomatic width	0.89	0.43	0.98	0.018	1.4
Body length	0.71	0.51	0.77	0.233	1.8
Tail length	0.35	0.92	0.98	0.024	1.3

Note: # represents Communality; ## represents Uniqueness; com represents Complexity.

Table S5 Summary of sequencing data from MGISEQ-2000 platform for *T. shortridgei*

Raw Data（Gb）	Clean Data（Gb）	Read length（bp）	Total read number	Sequence Coverage (×)
248	230.76	150	769,213,760	77

Table S6 Data statistics of Next generations of *T. shortridgei*

ID	Total_bases（Gb）	Total_reads	Average_Length（bp）	N50_Length（bp）	Sequence coverage（×）
WHDNA20201021-1	57.43	3,745,105	15,334	23,520	19.21
WHDNA20201027-4	122,70	8,572,442	15,205	24,560	26.39
WHDNA20201027-5	73,32	5,120,336	15,567	24,245	20.51
WHDNA20201022-4	70.64	4,734,075	16,062	24,748	23.63
WHDNA20201031-1	111.14	6,651,703	17,256	26,926	37.17
Total	435.23	28,931,661			126.91

Table S7 Length of each chromosome after Hi-C assembly

Super-Scaffold ID	Length（bp）	Super-Scaffold ID	Length（bp）	Super-Scaffold ID	Length（bp）
Chr1	197267371	Chr9	134094747	Chr17	104158986
Chr2	190392948	Chr10	133888297	Chr18	95033500
Chr3	188800100	Chr11	132614771	Chr19	79529619
Chr4	178937293	Chr12	132490127	Chr20	79518610
Chr5	174961031	Chr13	131791775	Chr21	66748657
Chr6	172911798	Chr14	126046897	Chr22	36123164
Chr7	150476594	Chr15	117026265		
Chr8	141543993	Chr16	113852662		

Table S8 Final genomic information of *T. shortridgei*

	Length（bp）	Number
N50	134,094,747	9
N90	95,033,500	18
Longest	197,267,371	-
Average length	4,645,359.77	-
Total size	2,898,704,498	-

Table S9 *T. shortridgei* Genome completeness based on BUSCO annotations

Type	Number	Percentage （%）
Complete BUSCOs (C)	8857	96.0
Complete and single-copy BUSCOs (S)	8598	93.2
Complete and duplicated BUSCOs (D)	259	2.8
Fragmented BUSCOs (F)	106	1.1
Missing BUSCOs (M)	263	2.9
Total BUSCO groups searched	9226	

Table S10 The read mapping rate of the assembled genome using BWA

	Number	Ratio（%）
Mapped reads	1304497019	99.92
PE mapped reads	1290653326	99.02
Total Reads	1305520574	-

Table S11 Repeat elements prediction in the genome of *T. shortridgei*

Type	Repeat Size（bp）	% of genome
de novo	1,285,579,885	44.35
Trf	87,679,136	3.02
Repeatmasker	1,409,317,451	48.62
Proteinmask	475,866,235	16.42
Total	1,487,125,633	51.30

Table S12 Different types of repeat elements prediction in the genome of *T. shortridgei*

Type	Repbase TEs		TE proteins		*de novo*		Combined TEs	
	Length（bp）	% in genome	Length （bp）	% in genome	Length （bp）	% in genome	Length （bp）	% in genome
DNA	100,562,718	3.47	15,620,061	0.54	66,314,389	2.29	108,456,912	3.74
LINE	635,865,975	21.94	399,145,408	13.77	631,388,916	21.78	656,147,601	22.64
SINE	420,114,553	14.49	000,000	0.00	272,650,995	9.41	458,110,902	15.80
LTR	251,766,162	8.69	61,100,766	2.11	259,502,625	8.95	263,402,175	9.09
Unknown	1,008,043	0.03	000,000	0.00	9,996,813	0.34	1,008,043	0.03
Total	1,409,317,451	48.62	475,866,235	16.42	1,239,853,738	42.77	1,487,125,633	51.30

Table S13 Gene structure prediction in the genome of *T. shortridgei*

Gene set		Number	Average transcript length （bp）	Average CDS length （bp）	Average exon length （bp）	Average intron length （bp）	Average exons per gene
de novo	Augustus	44,153	36,212	1,281	215	7,020	5.98
	geneid	112,208	16,366	1,169	329	5,941	3.56
	SNAP	97,298	49,851	755	144	1,1597	7.23
Homology	Human	25,281	19,893	1,055	184	3,971	6.74
	Rhirox	25,320	25,685	1,387	196	3,991	7.09
	Trafra	24,121	28,439	1,420	185	4,043	7.68
EVM		40,878	29,243	1,399	236	5,658	9.92
Clean set*		24,761	41,362	1,560	183	5,274	8.55

Table S14 *D*-statistic test results

P1	P2	P3	Dstatistic	Z-score
T.francoisi	*T.shortridgei*	*S.entellus*	-0.0259	-6.825
T.crepusculus	*T.shortridgei*	*S.entellus*	-0.0707	-18.379
T.germaini	*T.shortridgei*	*S.entellus*	-0.0272	-7.593
T.crepusculus	*T.francoisi*	*T.shortridgei*	-0.0667	-14.866
T.germaini	*T.francoisi*	*T.shortridgei*	0.03	8.189
T.shortridgei	*T.francoisi*	*S.entellus*	0.0259	6.825
T.crepusculus	*T.francoisi*	*S.entellus*	-0.0723	-17.873
T.shortridgei	*T.germaini*	*S.entellus*	0.0272	7.593
T.crepusculus	*T.germaini*	*S.entellus*	-0.0611	-15.642
T.crepusculus	*T.germaini*	*T.francoisi*	-0.1821	-39.897

Table S15 Summary of D_{FOIL} analysis per 5-taxon phylogeny based on 10 kb windows

P1	P2	P3	P4	Species Pair	Introgressed windows	Total windows
T.germaini	*T.francoisi*	*T.shortridgei*	*S.entellus*	P1P2,P3	28595	84710
T.germaini	*T.francoisi*	*T.shortridgei*	*S.entellus*	P1P2,P4	1250	84710
T.germaini	*T.francoisi*	*T.shortridgei*	*S.entellus*	P1→P3	142	84710
T.germaini	*T.francoisi*	*T.shortridgei*	*S.entellus*	P1→P4	1	84710
T.germaini	*T.francoisi*	*T.shortridgei*	*S.entellus*	P2→P3	202	84710
T.germaini	*T.francoisi*	*T.shortridgei*	*S.entellus*	P2→P4	9	84710
T.germaini	*T.francoisi*	*T.shortridgei*	*S.entellus*	P3→P1	34	84710
T.germaini	*T.francoisi*	*T.shortridgei*	*S.entellus*	P3→P2	29	84710
T.germaini	*T.francoisi*	*T.shortridgei*	*S.entellus*	P4→P1	3	84710
T.germaini	*T.francoisi*	*T.shortridgei*	*S.entellus*	P4→P2	3	84710
T.crepusculus	*T.francoisi*	*T.shortridgei*	*S.entellus*	P1P2,P3	30572	96573
T.crepusculus	*T.francoisi*	*T.shortridgei*	*S.entellus*	P1P2,P4	1302	96573
T.crepusculus	*T.francoisi*	*T.shortridgei*	*S.entellus*	P1→P3	160	96573
T.crepusculus	*T.francoisi*	*T.shortridgei*	*S.entellus*	P1→P4	6	96573
T.crepusculus	*T.francoisi*	*T.shortridgei*	*S.entellus*	P2→P3	328	96573
T.crepusculus	*T.francoisi*	*T.shortridgei*	*S.entellus*	P2→P4	8	96573
T.crepusculus	*T.francoisi*	*T.shortridgei*	*S.entellus*	P3→P1	37	96573
T.crepusculus	*T.francoisi*	*T.shortridgei*	*S.entellus*	P3→P2	121	96573
T.crepusculus	*T.francoisi*	*T.shortridgei*	*S.entellus*	P4→P1	3	96573
T.crepusculus	*T.francoisi*	*T.shortridgei*	*S.entellus*	P4→P2	17	96573
T.crepusculus	*T.germaini*	*T.shortridgei*	*S.entellus*	P1P2,P3	27881	86783
T.crepusculus	*T.germaini*	*T.shortridgei*	*S.entellus*	P1P2,P4	1178	86783
T.crepusculus	*T.germaini*	*T.shortridgei*	*S.entellus*	P1→P3	182	86783

T.crepusculus	*T.germaini*	*T.shortridgei*	*S.entellus*	P1→P4	5	86783
T.crepusculus	*T.germaini*	*T.shortridgei*	*S.entellus*	P2→P3	228	86783
T.crepusculus	*T.germaini*	*T.shortridgei*	*S.entellus*	P2→P4	10	86783
T.crepusculus	*T.germaini*	*T.shortridgei*	*S.entellus*	P3→P1	27	86783
T.crepusculus	*T.germaini*	*T.shortridgei*	*S.entellus*	P3→P2	74	86783
T.crepusculus	*T.germaini*	*T.shortridgei*	*S.entellus*	P4→P1	1	86783
T.crepusculus	*T.germaini*	*T.shortridgei*	*S.entellus*	P4→P2	8	86783
T.crepusculus	*T.germaini*	*T.francoisi*	*T.shortridgei*	P1P2,P3	10213	88461
T.crepusculus	*T.germaini*	*T.francoisi*	*T.shortridgei*	P1P2,P4	383	88461
T.crepusculus	*T.germaini*	*T.francoisi*	*T.shortridgei*	P1→P3	168	88461
T.crepusculus	*T.germaini*	*T.francoisi*	*T.shortridgei*	P1→P4	3	88461
T.crepusculus	*T.germaini*	*T.francoisi*	*T.shortridgei*	P2→P3	495	88461
T.crepusculus	*T.germaini*	*T.francoisi*	*T.shortridgei*	P2→P4	11	88461
T.crepusculus	*T.germaini*	*T.francoisi*	*T.shortridgei*	P3→P1	38	88461
T.crepusculus	*T.germaini*	*T.francoisi*	*T.shortridgei*	P3→P2	264	88461
T.crepusculus	*T.germaini*	*T.francoisi*	*T.shortridgei*	P4→P1	4	88461
T.crepusculus	*T.germaini*	*T.francoisi*	*T.shortridgei*	P4→P2	7	88461

Table S16 Summary of D_{FOIL} analysis per 4-taxon phylogeny based on 10 kb windows

P1	P2	P3	Species Pair	Introgressed windows	Total windows
T.francoisi	*T.shortridgei*	*S.entellus*	P2,P3	3610	108333
T.francoisi	*T.shortridgei*	*S.entellus*	P1,P3	2613	108333
T.crepusculus	*T.shortridgei*	*S.entellus*	P2,P3	3418	97955
T.crepusculus	*T.shortridgei*	*S.entellus*	P1,P3	2057	97955
T.germaini	*T.shortridgei*	*S.entellus*	P2,P3	3083	96680
T.germaini	*T.shortridgei*	*S.entellus*	P1,P3	2138	96680

Table S17 Number of blocks with consecutive 10-Kbp introgressed windows between *T.shortridgei* and *S.entellus* based on per 4-taxon phylogeny

P1	P2	P3	Species Pair	Number of consecutives introgressed windows in a block	Number of phylogenies
T.francoisi	*T.shortridgei*	*S.entellus*	P2,P3	1	344
T.crepusculus	*T.shortridgei*	*S.entellus*	P2,P3	1	314
T.germaini	*T.shortridgei*	*S.entellus*	P2,P3	1	316
T.francoisi	*T.shortridgei*	*S.entellus*	P2,P3	2	68
T.crepusculus	*T.shortridgei*	*S.entellus*	P2,P3	2	57
T.germaini	*T.shortridgei*	*S.entellus*	P2,P3	2	52
T.francoisi	*T.shortridgei*	*S.entellus*	P2,P3	3	16
T.crepusculus	*T.shortridgei*	*S.entellus*	P2,P3	3	14

T.germaini	*T.shortridgei*	*S.entellus*	P2,P3	3	12
T.francoisi	*T.shortridgei*	*S.entellus*	P2,P3	4	6
T.crepusculus	*T.shortridgei*	*S.entellus*	P2,P3	4	4
T.germaini	*T.shortridgei*	*S.entellus*	P2,P3	4	3
T.francoisi	*T.shortridgei*	*S.entellus*	P2,P3	5	2
T.crepusculus	*T.shortridgei*	*S.entellus*	P2,P3	5	1
T.germaini	*T.shortridgei*	*S.entellus*	P2,P3	5	2

Table S18 Summary of D_{FOIL} analysis for per 5-taxon phylogeny based on 100 kb windows

P1	P2	P3	P4	Species Pair	Introgressed windows	Total windows
T.germaini	*T.francoisi*	*T.shortridgei*	*S.entellus*	P1P2,P3	4318	5865
T.germaini	*T.francoisi*	*T.shortridgei*	*S.entellus*	P1P2,P4	31	5865
T.germaini	*T.francoisi*	*T.shortridgei*	*S.entellus*	P1→P3	37	5865
T.germaini	*T.francoisi*	*T.shortridgei*	*S.entellus*	P2→P3	61	5865
T.crepusculus	*T.francoisi*	*T.shortridgei*	*S.entellus*	P1P2,P3	4800	6818
T.crepusculus	*T.francoisi*	*T.shortridgei*	*S.entellus*	P1P2,P4	29	6818
T.crepusculus	*T.francoisi*	*T.shortridgei*	*S.entellus*	P1→P3	47	6818
T.crepusculus	*T.francoisi*	*T.shortridgei*	*S.entellus*	P2→P4	1	6818
T.crepusculus	*T.francoisi*	*T.shortridgei*	*S.entellus*	P2→P3	92	6818
T.crepusculus	*T.francoisi*	*T.shortridgei*	*S.entellus*	P3→P2	6	6818
T.crepusculus	*T.germaini*	*T.shortridgei*	*S.entellus*	P1P2,P3	4012	5547
T.crepusculus	*T.germaini*	*T.shortridgei*	*S.entellus*	P1P2,P4	29	5547
T.crepusculus	*T.germaini*	*T.shortridgei*	*S.entellus*	P1→P3	39	5547
T.crepusculus	*T.germaini*	*T.shortridgei*	*S.entellus*	P1→P4	5	5547
T.crepusculus	*T.germaini*	*T.shortridgei*	*S.entellus*	P3→P2	3	5547
T.crepusculus	*T.germaini*	*T.shortridgei*	*S.entellus*	P4→P2	1	5547
T.crepusculus	*T.germaini*	*T.francoisi*	*T.shortridgei*	P1P2,P3	2232	5686
T.crepusculus	*T.germaini*	*T.francoisi*	*T.shortridgei*	P1P2,P4	3	5686
T.crepusculus	*T.germaini*	*T.francoisi*	*T.shortridgei*	P1→P3	43	5686
T.crepusculus	*T.germaini*	*T.francoisi*	*T.shortridgei*	P2→P3	233	5686
T.crepusculus	*T.germaini*	*T.francoisi*	*T.shortridgei*	P3→P1	5	5686
T.crepusculus	*T.germaini*	*T.francoisi*	*T.shortridgei*	P3→P2	31	5686
T.crepusculus	*T.germaini*	*T.francoisi*	*T.shortridgei*	P4→P2	1	5686

Table S19 Summary of D_{FOIL} analysis for per 4-taxon phylogeny based on 100 kb windows

P1	P2	P3	Species Pair	Introgressed windows	Total windows
T.francoisi	*T.shortridgei*	*S.entellus*	P2,P3	201	7962
T.francoisi	*T.shortridgei*	*S.entellus*	P1,P3	125	7962
T.crepusculus	*T.shortridgei*	*S.entellus*	P2,P3	180	6957
T.crepusculus	*T.shortridgei*	*S.entellus*	P1,P3	80	6957

T.germaini	*T.shortridgei*	*S.entellus*	P2,P3	118	5997
T.germaini	*T.shortridgei*	*S.entellus*	P1,P3	72	5997
T.germaini	*T.francoisi*	*T.shortridgei*	P2,P3	22	6131
T.germaini	*T.francoisi*	*T.shortridgei*	P1,P3	28	6131
T.germaini	*T.crepusculus*	*T.shortridgei*	P2,P3	11	5807
T.germaini	*T.crepusculus*	*T.shortridgei*	P1,P3	19	5807

Table S20 The QuIBL analysis results

triplet	outgroup	C1	C2	mixprop1	mixprop2	lambda2Dist	lambda1Dist	count	BIC2Dist	BIC1Dist	BICdiff	isSigIntro	isSigILS	IsMostCommon
Tsho-Tger-Sent	Tger	0	3.225245	0.754632	0.245368	0.000551	0.000947	1294	-15332.41	-15424.2	91.79	FALSE	TURE	FALSE
Tsho-Tger-Sent	Tsho	0	3.381061	0.794419	0.205581	0.000538	0.000887	1212	-14518.93	-14604.3	85.37	FALSE	TURE	FALSE
Tsho-Tger-Sent	Sent	0	2.947414	0.669463	0.330537	0.000791	0.001424	11594	-128298.30	-128778.65	480.35	FALSE	TURE	TURE
Tsho-Tfra-Sent	Tfra	0	3.256222	0.757592	0.242408	0.000574	0.000986	1279	-15051.24	-15140.98	89.74	FALSE	TURE	FALSE
Tsho-Tfra-Sent	Tsho	0	3.006554	0.853906	0.146094	0.000632	0.000934	1205	-14289.58	-14393.98	104.40	FALSE	TURE	FALSE
Tsho-Tfra-Sent	Sent	0	2.956782	0.67321	0.32679	0.000794	0.001427	11616	-128493.35	-128986.65	493.30	FALSE	TURE	TURE
Tsho-Tcre-Sent	Tcre	0	3.074099	0.773081	0.226919	0.000596	0.000986	1300	-15283.26	-15389.02	105.76	FALSE	TURE	FALSE
Tsho-Tcre-Sent	Tsho	0	3.387054	0.852484	0.147516	0.000612	0.000927	1214	-14431.25	-14521.67	90.42	FALSE	TURE	FALSE
Tsho-Tcre-Sent	Sent	0	2.962677	0.671849	0.328151	0.000790	0.001422	11586	-128238.67	-128721.03	482.36	FALSE	TURE	TURE
Tsho-Tfra-Tcre	Tfra	0	4.797668	0.947546	0.052454	0.000400	0.000400	1614	-22120.65	-22023.56	-97.09	TURE	FALSE	FALSE
Tsho-Tfra-Tcre	Tcre	0	5.157759	0.960002	0.039998	0.000458	0.000458	1651	-22153.05	-22075.08	-77.97	TURE	FALSE	FALSE
Tsho-Tfra-Tcre	Tsho	0	4.617934	0.926266	0.073734	0.000535	0.000738	10835	-134434.69	-134596.94	162.25	FALSE	TURE	TURE
Tsho-Tfra-Tger	Tfra	0	5.25536	0.957424	0.042576	0.000404	0.000404	1405	-19218.15	-19140.61	-77.54	TURE	FALSE	FALSE
Tsho-Tfra-Tger	Tger	0	5.546553	0.956816	0.043184	0.000426	0.000426	1379	-18733.83	-18637.64	-96.19	TURE	FALSE	FALSE
Tsho-Tfra-Tger	Tsho	0	4.070003	0.92268	0.07732	0.000602	0.000824	11316	-137685.72	-138085.49	399.77	FALSE	TURE	TURE
Tsho-Tcre-Tger	Tcre	0	5.38638	0.967198	0.032802	0.000487	0.000487	1125	-14941.83	-14902.12	-39.71	TURE	FALSE	FALSE
Tsho-Tcre-Tger	Tger	0	4.58971	0.950678	0.049322	0.000421	0.000421	1086	-14751.02	-14705.52	-45.50	TURE	FALSE	FALSE
Tsho-Tcre-Tger	Tsho	0	3.813008	0.933519	0.066481	0.000695	0.000920	11889	-141959.58	-142451.57	491.99	FALSE	TURE	TURE

Note: C1, C2: The time since two sister species became isolated from the third species for a triplet topology under the ILS-only and the ILS+introgression distribution model, respectively; mixprop1, mixprop2: The inferred mixing proroptions for this two model distribution model, respectively; lambda2Dist, lambda1Dist: The scaling factor to convert the input branch length unit into coalescent units for this two model, respectively; BIC1Dist, BIC2Dist: The Bayesian Information Criterion scores for this two model, respectively; Count: The total number of trees supporting a triplet topology; BICdiff: ΔBIC; isSigIntro: Is the ILS+introgression model by supported; isSigILS: is the topology can be explained by ILS-only; IsMostCommon: Is the topology

supported by the most trees phylogenetically correct. Tger represents *T. germaini*, Tfra represents *T. francoisi*, Tcre represents *T. crepusculus*, Tsho represents *T. shortridgei*, and Sent represents *S. entellus*.

Table S21 The proportion of ILS observed in WGAs and CDS inferred by CoalHMM in three combinations

	type0		type1		type2		type3	
	WGAs	CDS	WGAs	CDS	WGAs	CDS	WGAs	CDS
Combination 1	78.74%	96.10%	4.55%	2.59%	7.80%	0.61%	8.91%	0.71%
Combination 2	78.19%	96.17%	5.19%	2.53%	7.90%	0.64%	8.72%	0.66%
Combination 3	77.37%	94.99%	5.49%	3.46%	8.31%	0.79%	8.83%	0.76%

Table S22 GO enrichment of 77 ILS genes in the *T. shortridgei* (FDR < 0.1)

Description	ID	N_{ILSGs}	Background number	FDR
chemokine activity	GO:0008009	3	48	0.0061653
antimicrobial humoral immune response mediated by antimicrobial peptide	GO:0061844	3	62	0.0061653
chemokine-mediated signaling pathway	GO:0070098	3	67	0.0061653
MHC protein binding	GO:0042287	2	10	0.0061653
oligosaccharide binding	GO:0070492	2	11	0.0061653
neutrophil chemotaxis	GO:0030593	3	77	0.0061653
eosinophil chemotaxis	GO:0048245	2	17	0.0095385
peptidoglycan binding	GO:0042834	2	18	0.0095385
chemotaxis	GO:0006935	3	119	0.0128745
CCR chemokine receptor binding	GO:0048020	2	28	0.0177439
peptide antigen binding	GO:0042605	2	31	0.0193092
lymphocyte chemotaxis	GO:0048247	2	32	0.0193092
extracellular region	GO:0005576	8	1843	0.0221427
monocyte chemotaxis	GO:0002548	2	43	0.0293071
response to peptide hormone	GO:0043434	2	48	0.0308884
G protein-coupled receptor signaling pathway	GO:0007186	6	1132	0.0308884
protein K48-linked ubiquitination	GO:0070936	2	54	0.03407
signaling receptor activity	GO:0038023	3	214	0.03407
nuclear chromatin	GO:0000790	5	915	0.0597647
cellular response to interleukin-1	GO:0071347	2	80	0.0616105
transcription, DNA-templated	GO:0006351	2	87	0.0616105
signal transduction	GO:0007165	5	1013	0.0616105
cellular response to interferon-gamma	GO:0071346	2	95	0.0616105
cellular calcium ion homeostasis	GO:0006874	2	98	0.0616105
RNA polymerase II cis-regulatory region sequence-specific DNA binding	GO:0000978	4	656	0.0616105
ubiquitin ligase complex	GO:0000151	2	107	0.0616105
metanephric epithelium development	GO:0072207	1	5	0.0616105
DNA topoisomerase binding	GO:0044547	1	5	0.0616105
negative regulation of B cell differentiation	GO:0045578	1	5	0.0616105
metanephric S-shaped body morphogenesis	GO:0072284	1	5	0.0616105
extracellular space	GO:0005615	6	1572	0.0616105
negative regulation of cardiac muscle hypertrophy in response to stress	GO:1903243	1	6	0.0616105
nucleoside triphosphate catabolic process	GO:0009143	1	6	0.0616105
pronephros development	GO:0048793	1	6	0.0616105

negative regulation by host of viral genome replication	GO:0044828	1	6	0.0616105
lymphocyte proliferation	GO:0046651	1	6	0.0616105
otic vesicle development	GO:0071599	1	6	0.0616105
free ubiquitin chain polymerization	GO:0010994	1	6	0.0616105
netrin receptor activity	GO:0005042	1	6	0.0616105
regulation of metanephric nephron tubule epithelial cell differentiation	GO:0072307	1	6	0.0616105
cellular response to gonadotropin stimulus	GO:0071371	1	6	0.0616105
inflammatory response	GO:0006954	3	381	0.0616105
cellular response to tumor necrosis factor	GO:0071356	2	125	0.0616105
negative regulation of stress-activated MAPK cascade	GO:0032873	1	7	0.0616105
phospholipase activator activity	GO:0016004	1	7	0.0616105
negative regulation of keratinocyte differentiation	GO:0045617	1	7	0.0616105
cellular response to hyperoxia	GO:0071455	1	7	0.0616105
regulation of intracellular signal transduction	GO:1902531	1	7	0.0616105
positive regulation of fibroblast growth factor receptor signaling pathway	GO:0045743	1	7	0.0616105
mesenchymal to epithelial transition involved in metanephros morphogenesis	GO:0003337	1	7	0.0616105
negative regulation of cell population proliferation	GO:0008285	3	394	0.0616105
nucleoside-triphosphatase activity	GO:0017111	1	8	0.0616105
DNA binding	GO:0003677	5	1209	0.0616105
T cell receptor complex	GO:0042101	2	138	0.0616105
growth cone	GO:0030426	2	140	0.0616105
regulation of neural precursor cell proliferation	GO:2000177	1	9	0.0616105
positive regulation of blood vessel endothelial cell proliferation involved in sprouting angiogenesis	GO:1903589	1	9	0.0616105
sarcoplasmic reticulum lumen	GO:0033018	1	9	0.0616105
arginine binding	GO:0034618	1	9	0.0616105
cytolysis by host of symbiont cells	GO:0051838	1	9	0.0616105
response to UV-B	GO:0010224	1	9	0.0616105
immune response	GO:0006955	3	427	0.0616105
olfactory receptor activity	GO:0004984	3	427	0.0616105
detection of chemical stimulus involved in sensory perception of smell	GO:0050911	3	427	0.0616105
organ induction	GO:0001759	1	10	0.0616105
retinal rod cell development	GO:0046548	1	10	0.0616105
midgut development	GO:0007494	1	10	0.0616105
response to UV-C	GO:0010225	1	10	0.0616105
regulation of mitotic metaphase/anaphase transition	GO:0030071	1	10	0.0616105
meiotic spindle	GO:0072687	1	10	0.0616105
netrin-activated signaling pathway	GO:0038007	1	10	0.0616105
embryonic skeletal joint morphogenesis	GO:0060272	1	10	0.0616105
positive regulation of leukocyte migration	GO:0002687	1	11	0.0621327

mesonephros development	GO:0001823	1	11	0.0621327
exit from mitosis	GO:0010458	1	11	0.0621327
retinal cone cell development	GO:0046549	1	11	0.0621327
GTP biosynthetic process	GO:0006183	1	11	0.0621327
cellular response to cold	GO:0070417	1	11	0.0621327
growth factor activity	GO:0008083	2	162	0.0621327
urogenital system development	GO:0001655	1	12	0.0621327
GATOR2 complex	GO:0061700	1	12	0.0621327
enamel mineralization	GO:0070166	1	12	0.0621327
photoreceptor cell outer segment organization	GO:0035845	1	12	0.0621327
heparin binding	GO:0008201	2	163	0.0621327
DNA-binding transcription activator activity, RNA polymerase II-specific	GO:0001228	3	466	0.0636603
cell projection membrane	GO:0031253	1	13	0.0637954
positive regulation of keratinocyte proliferation	GO:0010838	1	13	0.0637954
purine ribonucleoside monophosphate biosynthetic process	GO:0009168	1	13	0.0637954
DNA-binding transcription factor activity, RNA polymerase II-specific	GO:0000981	4	874	0.0637954
T cell receptor signaling pathway	GO:0050852	2	174	0.0640813
positive regulation of ubiquitin protein ligase activity	GO:1904668	1	14	0.0640813
positive regulation of gluconeogenesis	GO:0045722	1	14	0.0640813
maintenance of protein location in nucleus	GO:0051457	1	14	0.0640813
protein binding	GO:0005515	20	11779	0.0640813
CXCR chemokine receptor binding	GO:0045236	1	15	0.0640813
basal part of cell	GO:0045178	1	15	0.0640813
gamma-tubulin complex	GO:0000930	1	15	0.0640813
sulfur compound metabolic process	GO:0006790	1	15	0.0640813
protein acetylation	GO:0006473	1	15	0.0640813
nucleobase-containing small molecule catabolic process	GO:0034656	1	15	0.0640813
regulation of epithelial cell proliferation	GO:0050678	1	15	0.0640813
cellular response to nitric oxide	GO:0071732	1	16	0.0644045
cellular response to chemokine	GO:1990869	1	16	0.0644045
positive regulation of DNA-templated transcription, elongation	GO:0032786	1	16	0.0644045
dynactin complex	GO:0005869	1	16	0.0644045
SUMO transferase activity	GO:0019789	1	16	0.0644045
cytosol	GO:0005829	11	5095	0.065244
regulation of mitotic spindle organization	GO:0060236	1	17	0.065244
DNA binding, bending	GO:0008301	1	17	0.065244
regulation of ARF protein signal transduction	GO:0032012	1	17	0.065244
four-way junction DNA binding	GO:0000400	1	17	0.065244
positive regulation of interleukin-17 production	GO:0032740	1	18	0.0654743
negative regulation of TORC1 signaling	GO:1904262	1	18	0.0654743
negative regulation of myeloid cell	GO:0045638	1	18	0.0654743

differentiation				
positive regulation of branching involved in ureteric bud morphogenesis	GO:0090190	1	18	0.0654743
response to fatty acid	GO:0070542	1	18	0.0654743
regulation of reactive oxygen species metabolic process	GO:2000377	1	18	0.0654743
positive regulation of monocyte chemotaxis	GO:0090026	1	19	0.0677814
ARF guanyl-nucleotide exchange factor activity	GO:0005086	1	19	0.0677814
positive regulation of ERK1 and ERK2 cascade	GO:0070374	2	207	0.0678131
nucleus	GO:0005634	11	5208	0.0678131
temperature homeostasis	GO:0001659	1	20	0.0678131
positive regulation of cell migration involved in sprouting angiogenesis	GO:0090050	1	20	0.0678131
cellular glucose homeostasis	GO:0001678	1	20	0.0678131
endoderm development	GO:0007492	1	20	0.0678131
sequence-specific double-stranded DNA binding	GO:1990837	3	553	0.0678131
positive regulation of transcription, DNA-templated	GO:0045893	3	556	0.0679563
thyroid gland development	GO:0030878	1	21	0.0679563
cytoplasmic dynein complex	GO:0005868	1	21	0.0679563
negative regulation of cardiac muscle cell apoptotic process	GO:0010667	1	21	0.0679563
positive regulation of ubiquitin-protein transferase activity	GO:0051443	1	21	0.0679563
regulation of immune response	GO:0050776	2	221	0.0685511
anaphase-promoting complex	GO:0005680	1	22	0.0685511
calcium ion homeostasis	GO:0055074	1	22	0.0685511
cytoplasm	GO:0005737	10	4624	0.0685511
cell-cell signaling	GO:0007267	2	224	0.0685511
positive regulation of dendrite extension	GO:1903861	1	23	0.0685511
fibroblast growth factor binding	GO:0017134	1	23	0.0685511
neuronal stem cell population maintenance	GO:0097150	1	23	0.0685511
transcription coactivator binding	GO:0001223	1	23	0.0685511
regulation of mitotic nuclear division	GO:0007088	1	23	0.0685511
leukocyte chemotaxis	GO:0030595	1	23	0.0685511
ubiquitin-like protein ligase binding	GO:0044389	1	24	0.0699514
endocrine pancreas development	GO:0031018	1	24	0.0699514
positive regulation of wound healing	GO:0090303	1	24	0.0699514
ubiquitin-protein transferase activity	GO:0004842	2	233	0.0704621
retina layer formation	GO:0010842	1	25	0.0712943
positive regulation of innate immune response	GO:0045089	1	25	0.0712943
proximal/distal pattern formation	GO:0009954	1	26	0.0735196
actin cytoskeleton	GO:0015629	2	245	0.0752413
cellular response to DNA damage stimulus	GO:0006974	2	248	0.0764359
neuronal cell body membrane	GO:0032809	1	28	0.0773837
intrinsic apoptotic signaling pathway in	GO:0042771	1	29	0.0785187

response to DNA damage by p53 class mediator				
protein K11-linked ubiquitination	GO:0070979	1	29	0.0785187
metanephros development	GO:0001656	1	29	0.0785187
dendrite membrane	GO:0032590	1	30	0.0805922
transcription regulatory region sequence-specific DNA binding	GO:0000976	2	263	0.0811441
MyD88-dependent toll-like receptor signaling pathway	GO:0002755	1	31	0.0811441
cellular response to dexamethasone stimulus	GO:0071549	1	31	0.0811441
growth factor binding	GO:0019838	1	31	0.0811441
protein polyubiquitination	GO:0000209	2	267	0.0821465
liver regeneration	GO:0097421	1	32	0.0821507
protein phosphatase 2A binding	GO:0051721	1	32	0.0821507
ventricular septum development	GO:0003281	1	33	0.0836057
Rab guanyl-nucleotide exchange factor activity	GO:0017112	1	33	0.0836057
protein localization to nucleus	GO:0034504	1	34	0.0850245
protein monoubiquitination	GO:0006513	1	34	0.0850245
positive regulation of transcription by RNA polymerase II	GO:0045944	4	1159	0.0850634
energy homeostasis	GO:0097009	1	35	0.0855271
actin filament bundle assembly	GO:0051017	1	35	0.0855271
cytokine-mediated signaling pathway	GO:0019221	2	282	0.0855271
cell surface receptor signaling pathway	GO:0007166	2	286	0.0867779
acute-phase response	GO:0006953	1	36	0.0867779
ubiquitin-dependent protein catabolic process	GO:0006511	2	292	0.0890322
nucleosomal DNA binding	GO:0031492	1	39	0.0921206
photoreceptor connecting cilium	GO:0032391	1	40	0.0933476
rRNA binding	GO:0019843	1	40	0.0933476
response to nutrient levels	GO:0031667	1	41	0.094241
blood vessel development	GO:0001568	1	41	0.094241
positive regulation of GTPase activity	GO:0043547	2	307	0.094241
cytoplasmic translation	GO:0002181	1	42	0.0947089
ubiquitin conjugating enzyme activity	GO:0061631	1	42	0.0947089
branching involved in ureteric bud morphogenesis	GO:0001658	1	42	0.0947089
eye development	GO:0001654	1	43	0.095851
intrinsic apoptotic signaling pathway in response to DNA damage	GO:0008630	1	43	0.095851
G protein-coupled receptor activity	GO:0004930	3	739	0.0960697
anatomical structure development	GO:0048856	1	45	0.0988926
regulation of transcription by RNA polymerase II	GO:0006357	3	751	0.0988926
transcription factor binding	GO:0008134	2	325	0.0994861
negative regulation of fat cell differentiation	GO:0045599	1	46	0.0996289

Note: N_{ILSGs} represent the number of ILS genes on the GO entry.

Table S23 KEGG pathways enrichment of 77 ILS genes in the *T. shortridgei* (FDR < 0.1)

Description	ID	N_{ILSGs}	Background number	FDR
Viral protein interaction with cytokine and cytokine receptor	hsa04062	3	100	0.009539
Chemokine signaling pathway	hsa04062	3	190	0.03046
Cytokine-cytokine receptor interaction	hsa04060	3	294	0.061611
Purine metabolism	hsa00230	2	130	0.061611
Olfactory transduction	hsa04740	3	448	0.062133
Transcriptional misregulation in cancer	hsa05202	2	186	0.064405
Thyroid cancer	hsa05216	1	37	0.088581

Note: N_{ILSGs} represent the number of ILS genes on the KEGG pathways.

6 **Table S24 GO enrichment of 25 ILS genes in the *T. shortridgei* (FDR < 0.1)**

Description	ID	N_{ILSGs}	Background number	FDR
interleukin-17 receptor activity	GO:0030368	1	6	0.0695865
nucleoside triphosphate catabolic process	GO:0009143	1	6	0.0695865
negative regulation of cardiac muscle hypertrophy in response to stress	GO:1903243	1	6	0.0695865
netrin receptor activity	GO:0005042	1	6	0.0695865
cellular response to hyperoxia	GO:0071455	1	7	0.0695865
negative regulation of keratinocyte differentiation	GO:0045617	1	7	0.0695865
positive regulation of fibroblast growth factor receptor signaling pathway	GO:0045743	1	7	0.0695865
negative regulation of stress-activated MAPK cascade	GO:0032873	1	7	0.0695865
nucleoside-triphosphatase activity	GO:0017111	1	8	0.0695865
cytolysis by host of symbiont cells	GO:0051838	1	9	0.0695865
regulation of neural precursor cell proliferation	GO:2000177	1	9	0.0695865
positive regulation of blood vessel endothelial cell proliferation involved in sprouting angiogenesis	GO:1903589	1	9	0.0695865
netrin-activated signaling pathway	GO:0038007	1	10	0.0695865
meiotic spindle	GO:0072687	1	10	0.0695865
MHC protein binding	GO:0042287	1	10	0.0695865
cellular response to cold	GO:0070417	1	11	0.0695865
oligosaccharide binding	GO:0070492	1	11	0.0695865
enamel mineralization	GO:0070166	1	12	0.0695865
cell projection membrane	GO:0031253	1	13	0.0695865
positive regulation of keratinocyte proliferation	GO:0010838	1	13	0.0695865
positive regulation of gluconeogenesis	GO:0045722	1	14	0.0695865
nucleobase-containing small molecule catabolic process	GO:0034656	1	15	0.0695865
protein acetylation	GO:0006473	1	15	0.0695865
cellular response to nitric oxide	GO:0071732	1	16	0.0695865
regulation of ARF protein signal transduction	GO:0032012	1	17	0.0695865
regulation of mitotic spindle organization	GO:0060236	1	17	0.0695865
eosinophil chemotaxis	GO:0048245	1	17	0.0695865
peptidoglycan binding	GO:0042834	1	18	0.0695865
positive regulation of interleukin-17 production	GO:0032740	1	18	0.0695865
response to fatty acid	GO:0070542	1	18	0.0695865
regulation of reactive oxygen species metabolic process	GO:2000377	1	18	0.0695865
cytokine-mediated signaling pathway	GO:0019221	2	282	0.0695865
ARF guanyl-nucleotide exchange factor activity	GO:0005086	1	19	0.0695865
positive regulation of monocyte chemotaxis	GO:0090026	1	19	0.0695865
temperature homeostasis	GO:0001659	1	20	0.0695865
positive regulation of cell migration involved in sprouting angiogenesis	GO:0090050	1	20	0.0695865
cellular glucose homeostasis	GO:0001678	1	20	0.0695865
integral component of membrane	GO:0016021	6	3643	0.0695874
fibroblast growth factor binding	GO:0017134	1	23	0.0695874
regulation of mitotic nuclear division	GO:0007088	1	23	0.0695874
neuronal stem cell population maintenance	GO:0097150	1	23	0.0695874
transcription coactivator binding	GO:0001223	1	23	0.0695874

endocrine pancreas development	GO:0031018	1	24	0.0695874
positive regulation of wound healing	GO:0090303	1	24	0.0695874
CCR chemokine receptor binding	GO:0048020	1	28	0.077558
MyD88-dependent toll-like receptor signaling pathway	GO:0002755	1	31	0.077558
peptide antigen binding	GO:0042605	1	31	0.077558
growth factor binding	GO:0019838	1	31	0.077558
cellular response to dexamethasone stimulus	GO:0071549	1	31	0.077558
extracellular region	GO:0005576	4	1843	0.077558
lymphocyte chemotaxis	GO:0048247	1	32	0.077558
protein phosphatase 2A binding	GO:0051721	1	32	0.077558
Rab guanyl-nucleotide exchange factor activity	GO:0017112	1	33	0.0783796
energy homeostasis	GO:0097009	1	35	0.0799297
actin filament bundle assembly	GO:0051017	1	35	0.0799297
acute-phase response	GO:0006953	1	36	0.0806615
rRNA binding	GO:0019843	1	40	0.0846551
blood vessel development	GO:0001568	1	41	0.0846551
detection of chemical stimulus involved in sensory perception of smell	GO:0050911	2	427	0.0846551
olfactory receptor activity	GO:0004984	2	427	0.0846551
cytoplasmic translation	GO:0002181	1	42	0.0846551
eye development	GO:0001654	1	43	0.0846551
monocyte chemotaxis	GO:0002548	1	43	0.0846551
G protein-coupled receptor signaling pathway	GO:0007186	3	1132	0.0846551
anatomical structure development	GO:0048856	1	45	0.0854424
negative regulation of fat cell differentiation	GO:0045599	1	46	0.0854424
chemokine activity	GO:0008009	1	48	0.0856759
response to peptide hormone	GO:0043434	1	48	0.0856759
ribosome	GO:0005840	1	49	0.0856759
establishment or maintenance of cell polarity	GO:0007163	1	49	0.0856759
protein targeting to membrane	GO:0006612	1	53	0.0903024
antimicrobial humoral response	GO:0019730	1	54	0.0903024
cleavage furrow	GO:0032154	1	54	0.0903024
cytosolic large ribosomal subunit	GO:0022625	1	56	0.0922892
positive regulation of autophagy	GO:0010508	1	58	0.0929966
regulation of protein localization	GO:0032880	1	59	0.0933355
antimicrobial humoral immune response mediated by antimicrobial peptide	GO:0061844	1	62	0.0954759
negative regulation of transcription, DNA-templated	GO:0045892	2	536	0.0975885
activation of protein kinase activity	GO:0032147	1	66	0.0975885
cellular response to hydrogen peroxide	GO:0070301	1	67	0.0975885
chemokine-mediated signaling pathway	GO:0070098	1	67	0.0975885
cytoplasm	GO:0005737	6	4624	0.0975885
microvillus	GO:0005902	1	70	0.0987319
fat cell differentiation	GO:0045444	1	70	0.0987319
cellular response to starvation	GO:0009267	1	72	0.0991791
filopodium	GO:0030175	1	72	0.0991791
positive regulation of protein catabolic process	GO:0045732	1	73	0.0993942

Note: N_{ILSGs} represent the number of ILS genes on the GO entry.

Table S25 KEGG pathways enrichment of 77 ILS genes in the *T. shortridgei* (FDR < 0.1)

Description	ID	N_{ILSGs}	Background number	FDR
Olfactory transduction	hsa04740	2	448	0.08544
Pyrimidine metabolism	hsa00240	1	57	0.09265
Longevity regulating pathway - multiple species	hsa04213	1	62	0.095476

Note: N_{ILSGs} represent the number of ILS genes on the GO entry.

Table S26 ILS Genes underwent mutations at the same amino acid sites between *T. shortridgei* and *S. entellus* and gene expression at skeleton systems. The "Gene ID" column represents the gene ids in *M. mulatta*

Gene ID	Gene symbol	Number of unique substitutions	Gene expression
ENSMMUT00000089733	IL17REL	8	none
ENSMMUT00000019814	OR5A1	7	others
ENSMMUT00000090817	TRBV12-3	7	none
ENSMMUT00000087383	MYL10	4	none
ENSMMUT00000087493	PSD4	4	others
ENSMMUT00000082964	REG3G	3	others
ENSMMUT00000104563	UNC5CL	3	skeleton (basioccipital bone)
ENSMMUT00000011403	FGFBP1	2	skeleton (axial skeleton; bone)
ENSMMUT00000003988	OR10G2	2	none
ENSMMUT00000019583	RPL9	2	none
ENSMMUT00000043379	FOXO1	1	skeletal muscle
ENSMMUT00000043200	BORA	1	skeleton (temporal bone)
ENSMMUT00000100399	ENTPD3	1	skeleton (axial skeleton)
ENSMMUT00000001697	SPINK14	1	none
ENSMMUT00000013425	GARIN1B	1	none
ENSMMUT00000013670	CCL1	1	others
ENSMMUT00000021630	FSCN3	1	others
ENSMMUT00000025616	TAF11L2	1	none
ENSMMUT00000057504	GARIN1A	1	none
ENSMMUT00000069091	PRRT4	1	none
ENSMMUT00000079894	RABGEF1	1	others
ENSMMUT00000091981	SIX6	1	others
ENSMMUT00000094808	C9orf85	1	none
ENSMMUT00000095687	TEX44	1	none
ENSMMUT00000100862	LINC00596	1	none

Note: none represent no detectable expression information was found in the database; other represent gene expression detected in organs outside the skeletal system and skeletal muscle.

Table S27 Fluorescent Quantitative Primer Information

Name	Primer（5' to 3'）
FgF2-F	GCTGTACTGCAAAAACGGGG
FgF2-R	AGCCAGGTAACGGTTAGCAC
ALP-F	CATAGTCGGTGCTGTGGTGAAGG
ALP-R	GGGCTCTTGCGTGGGTTTCG
Runx2-F	CCACACCTACCTGCCACCAC
Runx2-R	TCCTGACGAAGTGCCATAGTAGAG
Ocn-F	CCCAGGCGCTACCTGTATCAA
Ocn-F	GGTCAGCCAACTCGTCACAGTC

Reference

1. L. E. Harding, *Trachypithecus cristatus* (Primates: Cercopithecidae). *Mamm. Species* **42**, 149-165 (2010).
2. N. Rowe, M. Myers, All the worlds primates. (U.K, Pogonias Press 2016).
3. K. Arekar, A. Parigi, K. Karanth, Understanding the convoluted evolutionary history of the capped-golden langur lineage (Cercopithecidae: Colobinae)†. *J. Genet.* **100** (2021).
4. H. Khajuria, XII.—A new Langur (Primates: Colobidae) from Goalpara District, Assam. *Ann. Mag. Nat. Hist.* **9**, 86-88 (1956).
5. J. Lever, M. Krzywinski, N. Altman, Principal component analysis. *Nat. Methods* **14**, 641-642 (2017).
6. L. Ren, *et al.*, Genomic and chromosomal architectures underlying fertility maintenance in the testes of intergeneric homoploid hybrids. *Sci. China Life Sci.* **68**, 2379-2392 (2025).
7. J. H. Ward, Hierarchical Grouping to optimize an objective function. *J. Am. Stat. Assoc.* **58**, 236-244 (1963).
8. L. Scrucca, C. Fraley, T. B. Murphy, R. Adrian E., Model-Based Clustering, Classification, and Density Estimation Using mclust in R, 1st Ed. (Chapman and Hall/CRC, 2023).
9. Y. Shao, *et al.*, Phylogenomic analyses provide insights into primate evolution. *Science* **380**, 913-924 (2023).
10. S. Kurtz, A. Narechania, J. C. Stein, D. Ware, A new method to compute K-mer frequencies and its application to annotate large repetitive plant genomes. *BMC Genomics* **9**, 517 (2008).
11. J. Hu, *et al.*, NextDenovo: an efficient error correction and accurate assembly tool for noisy long reads. *Genome Biol.* **25**, 107 (2024).
12. J. Hu, J. Fan, Z. Sun, S. Liu, NextPolish: a fast and efficient genome polishing tool for long-read assembly. *Bioinformatics* **36**, 2253-2255 (2020).
13. B. J. Walker, *et al.*, Pilon: An integrated tool for comprehensive microbial variant detection and genome assembly improvement. *PLoS ONE* **9**, e112963 (2014).
14. N. C. Durand, *et al.*, Juicer Provides a One-Click system for analyzing loop-resolution Hi-C experiments. *Cell Syst.* **3**, 95-98 (2016).
15. O. Dudchenko, *et al.*, *De novo* assembly of the *Aedes aegypti* genome using Hi-C yields chromosome-length scaffolds. *Science* **356**, 92-95 (2017).
16. F. A. Simão, R. M. Waterhouse, P. Ioannidis, E. V. Kriventseva, E. M. Zdobnov, BUSCO: assessing genome assembly and annotation completeness with single-copy orthologs. *Bioinformatics* **31**, 3210-3212 (2015).
17. M. J. Chaisson, G. Tesler, Mapping single molecule sequencing reads using basic local alignment with successive refinement (BLASR): application and theory. *BMC Bioinformatics* **13**, 238 (2012).
18. B. J. Pope, K. Mahmood, C. Jung, P. Georgeson, D. J. Park, Single nucleotide-level mapping of DNA double-strand breaks in human HEK293T cells. *Genomics Data* **11**, 43-45 (2017).
19. M. Tarailo-Graovac, N. Chen, Using RepeatMasker to identify repetitive elements in genomic sequences. *Curr. Protoc. Bioinforma.* **25** (2009).

20. G. Benson, Tandem repeats finder: a program to analyze DNA sequences. *Nucleic Acids Res.* **27**, 573-580 (1999).
21. I. Korf, Gene finding in novel genomes. *BMC Bioinformatics* **5**, 59 (2004).
22. W. H. Majoros, M. Pertea, S. L. Salzberg, TigrScan and GlimmerHMM: two open source *ab initio* eukaryotic gene-finders. *Bioinformatics* **20**, 2878-2879 (2004).
23. M. Stanke, *et al.*, AUGUSTUS: ab initio prediction of alternative transcripts. *Nucleic Acids Res.* **34**, W435-W439 (2006).
24. W. J. Kent, BLAT--The BLAST-like alignment tool. *Genome Res*. 12, 656-664
25. B. J. Haas, *et al.*, Automated eukaryotic gene structure annotation using EVidenceModeler and the program to assemble spliced alignments. *Genome Biol.* **9**, R7 (2008).
26. S. M. Kiełbasa, R. Wan, K. Sato, P. Horton, M. C. Frith, Adaptive seeds tame genomic sequence comparison. *Genome Res.* **21**, 487-493 (2011).
27. M. Blanchette, *et al.*, Aligning multiple genomic sequences with the threaded blockset aligner. *Genome Res.* **14**, 708-715 (2004).
28. J. Y. Dutheil, S. Gaillard, E. H. Stukenbrock, MafFilter: a highly flexible and extensible multiple genome alignment files processor. *BMC Genomics* **15**, 53 (2014).
29. A. Stamatakis, RAxML version 8: a tool for phylogenetic analysis and post-analysis of large phylogenies. *Bioinformatics* **30**, 1312-1313 (2014).
30. M. J. Hubisz, K. S. Pollard, A. Siepel, PHAST and RPHAST: phylogenetic analysis with space/time models. *Brief. Bioinform.* **12**, 41-51 (2011).
31. A. Siepel, *et al.*, Evolutionarily conserved elements in vertebrate, insect, worm, and yeast genomes. *Genome Res.* **15**, 1034-1050 (2005).
32. N. Dierckxsens, P. Mardulyn, G. Smits, NOVOPlasty: *de novo* assembly of organelle genomes from whole genome data. *Nucleic Acids Res.* gkw955 (2016).
33. R. C. Edgar, MUSCLE: multiple sequence alignment with high accuracy and high throughput. *Nucleic Acids Res.* **32**, 1792-1797 (2004).
34. R. R. Bouckaert, DensiTree: making sense of sets of phylogenetic trees. *Bioinformatics* **26**, 1372-1373 (2010).
35. C. Zhang, M. Rabiee, E. Sayyari, S. Mirarab, ASTRAL-III: polynomial time species tree reconstruction from partially resolved gene trees. *BMC Bioinformatics* **19**, 153 (2018).
36. L. Liu, L. Yu, S. V. Edwards, A maximum pseudo-likelihood approach for estimating species trees under the coalescent model. *BMC Evol. Biol.* **10**, 302 (2010).
37. E. Sayyari, J. B. Whitfield, S. Mirarab, DiscoVista: Interpretable visualizations of gene tree discordance. *Mol. Phylogenet. Evol.* **122**, 110-115 (2018).
38. Z. Yang, PAML: a program package for phylogenetic analysis by maximum likelihood. *Bioinformatics* **13**, 555-556 (1997).
39. N. G. Jablonski, *et al.*, Mesopithecus pentelicus from Zhaotong, China, the easternmost representative of a widespread Miocene cercopithecoid species. *J. Hum. Evol.* **146**, 102851 (2020).
40. J. B. Rossie, C. C. Gilbert, A. Hill, Early cercopithecid monkeys from the Tugen Hills, Kenya. *Proc. Natl. Acad. Sci.* **110**, 5818-5822 (2013).
41. D. M. Alba, *et al.*, First record of Mesopithecus (Cercopithecidae, Colobinae) from the Miocene of the Iberian Peninsula. *J. Hum. Evol.* **88**, 1-14 (2015).

42. E. Heintz, M. Brunet, B. Battait, A cercopithecid primate from the late miocene of Molayan, Afghanistan, with remarks on Mesopithecus. *Int. J. Primatol.* **2**, 273-284 (1981).
43. H. Li, R. Durbin, Inference of human population history from individual whole-genome sequences. *Nature* **475**, 493-496 (2011).
44. Y. Jung, D. Han, BWA-MEME: BWA-MEM emulated with a machine learning approach. *Bioinformatics* **38**, 2404-2413 (2022).
45. H. Li, *et al.*, The sequence alignment/map format and SAMtools. *Bioinformatics* **25**, 2078–2079 (2009).
46. C. Solís-Lemus, P. Bastide, C. Ané, PhyloNetworks: A package for phylogenetic networks. *Mol. Biol. Evol.* **34**, 3292-3298 (2017).
47. N. Patterson, *et al.*, Ancient admixture in human history. *Genetics* **192**, 1065-1093 (2012).
48. R. E. Green, *et al.*, A Draft sequence of the neandertal genome. *Science* **328**, 710-722 (2010).
49. J. B. Pease, M. W. Hahn, Detection and polarization of introgression in a five-taxon phylogeny. *Syst. Biol.* **64**, 651-662 (2015).
50. K. Węcek, *et al.*, Complex admixture preceded and followed the extinction of wisent in the wild. *Mol. Biol. Evol.* msw254 (2016).
51. N. B. Edelman, *et al.*, Genomic architecture and introgression shape a butterfly radiation. *Science* **366**, 594-599 (2019).
52. A. Hobolth, J. Y. Dutheil, J. Hawks, M. H. Schierup, T. Mailund, Incomplete lineage sorting patterns among human, chimpanzee, and orangutan suggest recent orangutan speciation and widespread selection. *Genome Res.* **21**, 349-356 (2011).
53. C. Xie, *et al.*, KOBAS 2.0: a web server for annotation and identification of enriched pathways and diseases. *Nucleic Acids Res.* **39**, W316-W322 (2011).
54. J. L. King, T. H. Jukes, Most evolutionary change in proteins may be due to neutral mutations and genetic drift. **164** (1969).
55. C. M. Smith, *et al.*, The mouse Gene Expression Database (GXD): 2019 update.
56. S. El-Gebali, *et al.*, The Pfam protein families database in 2019. *Nucleic Acids Res.* **47**, D427-D432 (2019).
57. J. Jumper, *et al.*, Highly accurate protein structure prediction with AlphaFold. *Nature* **596**, 583-589 (2021).
